# Top-Down Proteomics with Mass Spectrometry Imaging: A Pilot Study towards Discovery of Biomarkers for Neurodevelopmental Disorders

**DOI:** 10.1371/journal.pone.0092831

**Published:** 2014-04-07

**Authors:** Hui Ye, Rakesh Mandal, Adam Catherman, Paul M. Thomas, Neil L. Kelleher, Chrysanthy Ikonomidou, Lingjun Li

**Affiliations:** 1 State Key Laboratory of Natural Medicines, Key Lab of Drug Metabolism and Pharmacokinetics, China Pharmaceutical University, Nanjing, PR China; 2 Department of Pharmaceutical Analysis, China Pharmaceutical University, Nanjing, PR China; 3 School of Pharmacy, University of Wisconsin Madison, Madison, Wisconsin, United States of America; 4 Department of Neurology, University of Wisconsin Madison, Madison, Wisconsin, United States of America; 5 Proteomics Center of Excellence, Northwestern University, Evanston, Illinois, United States of America; 6 Department of Chemistry, University of Wisconsin Madison, Madison, Wisconsin, United States of America; Ludwig-Maximilians-Universität München, Germany

## Abstract

In the developing mammalian brain, inhibition of NMDA receptor can induce widespread neuroapoptosis, inhibit neurogenesis and cause impairment of learning and memory. Although some mechanistic insights into adverse neurological actions of these NMDA receptor antagonists exist, our understanding of the full spectrum of developmental events affected by early exposure to these chemical agents in the brain is still limited. Here we attempt to gain insights into the impact of pharmacologically induced excitatory/inhibitory imbalance in infancy on the brain proteome using mass spectrometric imaging (MSI). Our goal was to study changes in protein expression in postnatal day 10 (P10) rat brains following neonatal exposure to the NMDA receptor antagonist dizocilpine (MK801). Analysis of rat brains exposed to vehicle or MK801 and comparison of their MALDI MS images revealed differential relative abundances of several proteins. We then identified these markers such as ubiquitin, purkinje cell protein 4 (PEP-19), cytochrome c oxidase subunits and calmodulin, by a combination of reversed-phase (RP) HPLC fractionation and top-down tandem MS platform. More in-depth large scale study along with validation experiments will be carried out in the future. Overall, our findings indicate that a brief neonatal exposure to a compound that alters excitatory/inhibitory balance in the brain has a long term effect on protein expression patterns during subsequent development, highlighting the utility of MALDI-MSI as a discovery tool for potential biomarkers.

## Introduction

Sedatives, anaesthetics and anticonvulsants, such as ketamine, nitrous oxide (laughing gas), propofol, sevofluran, benzodiazepines and barbiturates are used frequently in obstetric and pediatric medicine. Neurological abnormalities associated with developmental exposure to such drugs and to environmental toxins, such as ethanol, have been reported. It has been suggested that disturbances in neurotransmitter signalling during critical periods can redirect subsequent development. Detrimental long-term effects observed in humans range from mild neurobehavioral disturbances (hyperactivity/attention deficit and learning disabilities) to severe mental retardation and autism spectrum disorders (ASD) [1], [2]. Interestingly, a potential unifying explanation for the pathogenesis of ASD is the disruption of excitatory/inhibitory circuit balance during critical periods of development [3]. Excitatory/inhibitory neurotransmission balance is important for single neurons to acquire multiple functional properties through an experience-dependent maturation process, which occurs during critical periods of brain development.

Previously we provided evidence that excitation/inhibition imbalance in early development can cause long lasting changes of the brain proteome [4]. In addition, compounds which inhibit NMDA receptors or activate γ-aminobutyric acid A (GABA_A_) receptors and cause excitation/inhibition imbalance, can induce widespread neuroapoptosis in the developing rodent brain, inhibit neurogenesis [5] and cause impairment of learning and memory [6]. Interestingly, early disruption of excitatory/inhibitory balance in infant mice (P0–P7) via blockade of NMDA receptors or activation of GABA_A_ receptors caused very similar acute and long-term changes of the cerebral proteome [4]. Changes of the cortex proteome one day (P7), one week (P14) and four weeks (P35) following treatment at P6 suggest that short period suppression of synaptic neurotransmission during critical periods of brain development causes long lasting dysregulation of proteins associated with apoptosis, oxidative stress, inflammation, proliferation and neuronal circuit formation [4]. These observations support the hypothesis that early disruption of excitatory/inhibitory balance in the brain interferes with many subsequent critical developmental events.

The integration of gel electrophoresis and liquid chromatography coupled to MS enables more comprehensive characterization of complex proteomes to detect disease biomarkers [7], [8], [9], [10] or evaluate the response to stress or to the exposure of compounds of interest [11], [12], [13], [14]. Previous studies of Kaindl et al. examined the whole cortex proteome after treatment with MK801 in infancy using this conventional approach [4], yet more precise localization of the detected protein expression changes has not been conducted. Moreover, our previous observation of MK801-treated rat brains visualized by terminal deoxynucleotidyl transferase–mediated dUTP nick-end labeling (TUNEL) exhibited a marked increase of neuron apoptosis in specific brain regions other than cortex, dictating the demand for a global examination of the entire brain to discover putative protein biomarkers related to the apoptotic process. Conventional immunohistochemical (IHC) staining allows for obtaining high resolution distribution images of targeted proteins. However, a significant limitation to this method is the need for labeling, which means that the target molecules must be known prior to the experiment. Alternatively, mass spectrometric imaging (MSI) has evolved as a powerful tool based on matrix-assisted laser desorption/ionization mass spectrometry (MALDI-MS). This technique allows for the determination of the mass and location of biomolecules directly from a tissue sample [15], [16], [17], [18]. MALDI MSI has enormous advantages over conventional protein imaging techniques in that not only it is label-free but also it enables simultaneous mapping of numerous molecules in tissue samples with great sensitivity and chemical specificity. MALDI MSI has proven to be a valuable technology with numerous applications in localizing proteins [19], [20], [21], [22], examining lipid distributions [23], [24], mapping neuropeptides [25], [26] and imaging drugs or metabolites [27] at both organ and cellular levels by varying the experimental conditions. Since no prior knowledge of molecular identities is required for MSI applications, it has become a useful biomarker discovery tool to compare analyte expression pattern changes by analyzing multiplexed data sets. For example, Minerva et al. evaluated the capability of MALDI-MS to study peptide expression patterns in the mouse pancreas under normal and pathological conditions and identified a peptide marker, the C-peptide of insulin, which was dramatically up-regulated in obese mice [28]. Sköld et al. observed a significant decrease of a large neuron-specific peptide, PEP-19, in a mouse model of Parkinson's disease (PD) using MALDI-MSI [29]. Hanrieder et al. quantified relative abundances of striatal neuropeptides in a rat model of L-DOPA-induced dyskinesia (LID) and revealed several upregulated dynorphins and enkephalins in the dorsolateral striatum of high-dyskinetic rats [30]. Two prodynorphin derived peptides, dynorphin B and α-neoendorphin, were positively associated with LID severity. Li and Hummon further explored the application of MALDI-MSI to image proteins from 3D colon carcinoma cell cultures and visualized cytochrome C and histone H4 in the cell spheroid [31]. In clinical settings, MALDI MSI has successfully enabled discovery of tissue biomarkers for diseases such as myxoid sarcomas [32], adenocarcinoma [33], gastric cancer [34], hypertension [35], metastatic melanoma [36] and prostate cancer [20]. These findings underscore the critical role MALDI-MSI plays in the study of peptide/protein dynamics in the context of human diseases. With MSI, a better understanding of the molecular pathology of various diseases such as cancer and neurodegenerative diseases can be obtained.

Although the discrimination of protein expression patterns of tissues harvested from disease and normal animals could be accomplished by MALDI-MSI, it remains to be a bottleneck to identify proteins via MALDI-MS alone due to limited fragmentation obtained from the singly charged high-mass species except fairly abundant ones [37]. Bottom-up MS of tryptic-digested proteins via *in situ* enzymatic digestion [38] or in-solution tissue extract digestion [39] is commonly attempted to interpret peaks with protein identities. Nevertheless, matching bottom-up identified peptides with intact proteins, especially those with isoforms, detected by MALDI-MSI is significantly challenging. Alternatively, recent advances in top-down MS and various dissociation methods facilitate the process by offering capability to fragment and ultimately identify intact proteins. Recent studies have shown that tissue extraction, fractionation followed by top-down tandem MS allows for direct identifications of intact proteins [19], [40], [41], [42]. For example, Meistermann unambiguously identified transthyretin (Ser^28^-Gln^146^) as a potential toxicity biomarker in kidneys of rats that were administered the nephrotoxin gentamicin [41]. Lagarrigue identified seven potential biomarkers of childhood absence epilepsy as Synapsin-1 fragments [43]. Stella et al and Schey et al. both localized and assigned identities of lens proteins and their truncated forms using a combined MALDI MSI and top-down proteomics approach, a task difficult to complete via a bottom-up approach [44], [45].

Herein, we employed MALDI-MS profiling to investigate region specific changes in the brain proteome following blockade of NMDA receptors in infancy and employed MALDI-MSI to localize these differentiating proteins with relatively high spatial resolution. In the present study, we first identified 22 potential protein biomarkers using this combined strategy. We then extracted proteins from tissues followed by RP HPLC fractionation and top-down sequencing by high resolution tandem MS for confident protein identification.

## Materials and Methods

### Chemicals and materials

Methanol, acetonitrile, formic acid and gelatin were purchased from Fisher Scientific (Pittsburgh, PA). All water used in this study was Milli-Q water from a Millipore filtration system (Bedford, MA).

### Animal Experiments

All animal procedures were reviewed and approved by the Institutional Animal Care and Use Committee (IACUC) of the Graduate School of the University of Wisconsin-Madison. Animal care facilities and procedures are yearly inspected and accredited by AAALAC (A3368-01). Wistar rats were injected i.p. with the NMDA receptors antagonist dizocilpine ((+)MK801; Tocris, Bristol, UK) 1 mg/kg i.p. on P2 and P4. An equal number of sex-matched littermates that received vehicle (normal saline) served as controls. Rats were terminally anesthetized with 5% isoflurane, transcardially perfused under deep anesthesia with cold saline solution for 5 min at room temperature and immediately decapitated on P10 (N = 4 per group).

### Sample Preparation for MALDI-MS

Rat brains were dissected out freshly and cut along the midline to halves. Then the hemispheres were immediately embedded into gelatin solution (100 mg/ml) and snap-frozen in −80°C freezer for further processing. The time between decapitation and freezing of the tissue did not exceed 5 min. Sagittal sections were acquired at a thickness of 12 µm on a cryostat (HM525, Thermo Fisher Scientific, Waltham, MA) and four sections from each animal were deposited onto indium tin oxide (ITO) coated conductive glass slides (Delta Technologies, Loveland, CO) as technical replicates. Tissue sections were immersed in 70% ethanol for 1 min and another 95% ethanol bath for 1 min for protein fixation and lipid removal. The sections were then allowed to dry for 30 min under vacuum. For profiling purpose, matrix deposition was conducted manually with a pipette directly on tissue sections. 300 nL of freshly prepared 20 mg/ml sinapinic acid (Sigma Aldrich, St. Louis, MO) dissolved in 50% acetonitrile, 0.1% formic acid were sequentially deposited onto 14 regions of a brain section. For imaging purposes, 20 mg/ml sinapinic acid dissolved in 50% acetonitrile, 0.1% formic acid was sprayed onto a brain section homogenously using an airbrush held 35 cm from the tissue. Five coats were applied and the spray duration for each coat was 30 s with 1 min dry time between each cycle.

### MALDI-MS Profiling and Imaging

MALDI-MS profiling spectra of the manually spotted tissue sections and imaging data of intact brain sections were acquired on an Autoflex III MALDI-TOF/TOF mass spectrometer (Bruker Daltonics, Billerica, MA) equipped with a 200 Hz smart beam laser. The following parameters were adopted in the positive linear mode at a mass range of 3–25 kDa for spectral acquisition with a delayed extraction of 50 ns: ion source 1 voltage 20.00 kV, ion source 2 voltage 18.55 kV, lens voltage 6.80 kV and pulsed ion extraction 130 ns. Protein calibration standard I (Bruker Daltonics, Billerica, MA) was used to externally calibrate the instrument before data acquisition. Briefly, 2000 consecutive laser shots were accumulated for each deposited matrix droplet. The profiling spectra were smoothed and baseline subtracted using flexAnalysis (Bruker Daltonics, Billerica, MA). As for imaging, automated MSI acquisitions of the brain sections were controlled by flexImaging software (Bruker Daltonics, Billerica, MA). Array of spectra was collected at 200 µm intervals in both x and y dimensions, and each spectrum consists of 200 laser shots.

### MALDI-MS Data Analysis

The MATLAB-supported software ClinProTools 2.2 (Bruker Daltonics, Billerica, MA) enables baseline subtraction, normalization, peak calibration, spectral alignment and statistical analyses of the resulting profiling spectra. The profiling spectra were divided as the vehicle-treated and the MK801-treated class and loaded into the software following the generation of relevant XML files with a ClinProtSpectraImport generator. The opened XML files were then prepared by recalibration, average peak list calculation, and peak calculation. By averaging, a single representative spectrum composed of 14 discrete spots from each section was generated for each class, which was used in all of the statistical analyses. The profiling spectra from the two classes were also compared region-wise for evaluating the sensitivity of cells in various regions in response to the MK801 treatment. Four serial sections were collected and profiled for four animals, with their intensities averaged for each group. PCA plot and loading plot were generated for each comparison group (N = 4). Peak statistics exported the mass, mass deviation, averaged intensities, the *P* value of Anderson-Darling test (PAD), the *P* value of the Wilcoxon test/Kruskal-Wallis test (PWKW) and *P* value of the t test/ANOVA (PTTA) of the individual peaks, whereas PTTA was used throughout this study. Models of control and treated groups were generated using available algorithm, genetic algorithm, where the minimum number of peaks was set at 10 and the maximum set as “default”. The recognition capability and cross validation were calculated to 98.50% and 91.50%, indicating the reliability and accuracy of the generated model.

For image processing, the Bruker proprietary software flexImaging 3.0 (Bruker Daltonics, Billerica, MA) was utilized to generate MS images and overlay the optical image with the MS images for good visualization. One section from each sacrificed animal was collected (N = 4) and MS images were acquired from sections collected from animals of two classes. MSI data were also imported into ClinProTools to analyze differences in abundance of proteins in the two animal groups. For comparison, specific regions of interest (ROI) were selected manually and the spectra resulting from the ROI were averaged and compared in ClinProTools. PCA plots and loading plots were generated for each comparison group.

### Liquid-phase Protein Extraction and Fractionation

Hemisphere of rat brain was manually homogenized in acidified methanol (1∶9∶90 acetic acid/water/methanol) at a 1∶5 ratio (tissue/buffer, v/v) on ice. The extract was centrifuged at 16,100 rcf for 10 min in an Eppendorf 5415 D microcentrifuge (Brinkman Instruments), and the resulting supernatant was collected followed by vacuum drying. The mixture was then resuspended in 30 µL of water containing 0.1% formic acid and separated on a 2.1×100 mm Phenomenex Kinetex 2.6 µm particle C18 reversed phase (RP) HPLC column using a 60 min gradient with a flow rate at 0.3 mL/min. Fractions were collected every three minutes and concentrated in a Savant SC 110 Speedvac concentrator (Thermo Electron Corporation, West Palm Beach, FL). The intact protein fractions were reconstituted in 0.1% formic acid and screened by MS profiling using MALDI-TOF/TOF via on-plate mixing with sinapinic acid. The spots were analyzed according to previous instrumentation settings and matched to *m/z* values obtained with *in situ* tissue profiling.

### On-line Top-down MS/MS using Nano-LC-ESI-LTQ-Orbitrap Elite

The fractions that contained peaks of interest were further analyzed by on-line top-down MS on an Ultimate 3000 RSLCnano system coupled to an Orbitrap Elite mass spectrometer (Thermo Fisher Scientific, Bremen, Germany). Fractions of interest (0.5 µL) were injected onto a 2 cm, 150 µm i.d. PLRP-S (d_p_ 5 µm, pore size 1000 Å) trap column. A 10 cm, 75 µm i.d. PLRP-S column was used for separation. The mobile phases used were: deionized water with 0.1% formic acid (A); acetonitrile with 0.1% formic acid (B). The gradient was delivered at 300 nL/min starting at 5% B and rose to 10% B at 7 min, 50% B at 50 min, and 85% B at 58 min. The mass spectrometer was operated in a data-dependent mode, performing higher-energy C-trap collision induced dissociation (HCD)-MS^2^ (scan 1), conventional collision induced dissociation (CID)-MS^2^ (scan 2) and electron transfer dissociation (ETD)-MS^2^ (scan 3) on each of the Top 3 precursors (selected by intact mass) in a FT-MS precursor scan. More details on mass spectrometric acquisition parameters can be found in Method S1 in **File S1**.

Data were deisotoped with Xtract using the cRAWler algorithm (ThermoFisher, Bremen, Germany) and searched with a custom 168-core ProSightPC 3.0 cluster (ThermoFisher, Bremen, Germany) using an iterative search tree. The detailed information on search tree can be found in **Method S1 in File S1**. ProSightPC Warehouses were created against the UniProt 2011_04 build filtered for *Rattus norvegicus* (TaxId: 10116). The “simple” rat database was created with all base sequences (signal peptide cleavage products, alternative splice forms, etc) and N-terminal acetylation applied. The simple database has 33,998 base sequences and 109,973 modified forms. The “complex” rat database contains all of the information in the simple database in addition to the annotated post-translational modifications in UniProt. The complex database had 40,990 base sequences and 3,616,723 modified forms. All retrieved proteins are considered as positive identification if: (a) an expect score (E value) less than 10^−5^, and (b) mass error (Δm) less than 5 ppm between the calculated and expected intact masses [46]. The protein *m/z* observed on MALDI-MS, entry name, accession number, protein identity, calculated mass deconvoluted from the *m/z* observed on LTQ Orbitrap, expected mass, average mass, post translational modifications (PTMs) present in the sequence and E value of the search results are all listed in **Table 1** **–** **2** and **Table S1, S2 in File S1**. The fragment ions assignments for identified proteins were summarized in **File S2 (top-down MSMS fragmentation)**, whereas the raw MS/MS spectra were recorded in **File S3 (top-down MSMS spectra)**.

**Table 1:** List of differentially-expressed proteins in rat brain sections observed on MALDI-TOF/TOF and identified by mass matching.

*m/z* on MALDI-MS	Entry	Accession	Protein Identity	Calc'd Mass (Da)	Exp'd Mass (Da)	Δ Mass (ppm)	Average Mass (Da)	PTTA	Ratio (MK/Veh)
**6718**	PCP4_RAT	P63055	*PEP-19*	6714.25	6714.26	−1	6718.19	<0.001	1.8
**11367**			Unknown					<0.001	1.8
**8566**	RS27A_RAT	P62982	*Ubiquitin*	8559.61	8559.62	−1	8564.76	<0.001	1.6
**5634**			Acyl-CoA-binding protein					<0.001	1.5
**4964**	TYB10_RAT	P63312	*Thymosin β-4*	4960.48	4960.49	−2	4963.45	<0.001	1.4
**9939**	ACBP_RAT	P11030	*Acyl-CoA-binding protein*	9932.12	9932.12	0	9938.19	<0.001	1.4
**9979**	D3ZD09_RAT	D3ZD09	*Cytochrome c oxidase subunit 6b*	9971.80	9971.82	−2	9978.17	<0.001	1.4
**5486**	B2RYT3_RAT	B2RYT3	*Cytochrome c oxidase subunit 7c*	5481.87	5481.87	0	5485.40	<0.001	1.3
**8368**	CX6C2_RAT	P11951	Cytochrome c oxidase subunit 6C					<0.001	1.2
**10283**	TIM9_RAT	Q9WV97	***Mitochondria import inner membrane translocase***	10276.08	10276.09	−1	10282.66	<0.05	1.2
**9193**	TIM8B_RAT	P62078	***Adenylate cyclase type 10***	9187.44	9187.45	−1	9193.22	<0.01	1.2
**9727**	ACBP_RAT	P11030	Acyl-CoA-binding protein					<0.001	1.1
**8040**	UCRI_RAT	P20788	***Cytochrome b-c1 complex subunit***	8035.38	8035.38	0	8040.30	<0.05	1.1
**4936**	TYB10_RAT	P63312	*Thymosin beta-10*	4933.51	4933.52	−2	4936.47	<0.05	1.1
**3430**	ATP5J_RAT	P21571	***ATP synthase-coupling factor 6***	3426.89	3426.90	−3	3428.94	<0.05	0.9
**16792**	CALM_RAT	P62161	*Calmodulin*	16779.82	16779.81	1	16790.32	<0.01	0.9
**3717**	Q5U2U9_RAT	Q5U2U9	***CCR4-NOT transcription complex, subunit 8***	3713.87	3713.88	−3	3716.25	<0.01	0.9
**3891**	PCSK1_RAT	Q9QXU9	***ProSAAS***	3888.04	3888.04	0	3890.32	<0.05	0.9
**4800**	SCG2_RAT	P10362	***Secretogranin-2***	4796.37	4796.37	0	4799.25	<0.01	0.9
**4854**	VGF_RAT	P20156	***Neurosecretory protein VGF***	4850.41	4850.41	0	4853.24	<0.05	0.9
**15825**	SODC_RAT	P07632	***Superoxide dismutase [Cu-Zn]***	15810.82	15810.75	4	15820.33	<0.05	0.8
**11348**			Unknown					<0.001	0.6

• The italicized protein sequences have been verified by top-down tandem mass spectrometry. The rest of the identity assignments are solely based on mass matching. The proteins that can be identified by neither approach were shown as unknown.

• The bolded sequences represent the proteins that were reported from rat brain in a MALDI-MS-based platform for the first time.

The proteins that were further identified by top-down MS/MS were highlighted in italics and annotated with UniProt entry, accession number, calculated mass based on the m/z observed on Orbitrap Elite, expected mass based on the matched protein sequence and the mass difference between the calculated mass and expected mass. All MALDI-MS profiling spectra have been processed by ClinProTools and summarized here by “peak statistic” function. PTTA represents p-value of t-test (2 classes); Ratio represents the relative abundance changes of these proteins in the brain sections of rats treated with MK801 and vehicle, calculated based on the average intensity of the ion observed in the MK801-treated class over the vehicle-treated class.

**Table 2:** List of proteins that displayed consistent abundance levels in MK801-treated and vehicle-treated rat brain sections via MALDI-MS.

*m/z* on MALDI-MS	Entry	Accession	Protein Identity	Calc'd Mass (Da)	Exp'd Mass (Da)	Δ Mass (ppm)	Average Mass (Da)	PTM	E value
3327	VIP_RAT	P01283	VIP peptides	3323.75	3323.76	−3	3325.80	C-term amidation	8.3E-14
3437	7B2_RAT	P27682	Neuroendocrine protein 7B 2	3433.78	3433.79	−3	3435.87		7.2E-39
3464	NPY_RAT	P07808	Pro-Neuropeptide Y	3460.66	3460.66	0	3462.82		1.6E-18
3593	7B2_RAT	P27682	Neuroendocrine protein 7B 2	3589.89	3589.89	0	3592.06		2.5E-60
3653	SCG2_RAT	P10362	Secretogranin 2	3649.80	3649.80	0	3651.94		3.8E-67
3676	VGF_RAT	P20156	Neurosecretory protein VGF	3672.77	3672.78	−3	3674.90		8.0E-08
3871	VGF_RAT	P20156	Neurosecretory protein VGF	3867.86	3867.87	−3	3870.12		2.9E-37
4273	NPY_RAT	P07808	Proneuropeptide Y	4269.08	4269.08	0	4271.69	C-term amidation	3.6E-42
4331	NPY_RAT	P07808	Proneuropeptide Y	4327.08	4327.09	−2	4329.73		1.6E-42
4522	TYB10_RAT	P63312	Thymosin beta-10	4517.33	4517.33	0	4520.04	N-term acetylation	2.2E-70
4738	TYB10_RAT	P63312	Thymosin beta-10	4733.40	4733.41	−2	4736.23	N-term acetylation	1.7E-89
4871	SCG2_RAT	P10362	Secretogranin-2	4867.40	4867.41	−2	4870.33		1.2E-77
7114	FILUV9_RAT	FILUV9	Uncharacterized	7108.52	7108.51	1	7112.62		4.9E-42
8468	UBB_RAT	P0CG51	Polyubiquilin	8461.56	8461.57	−1	8466.65	Oxidation	2.9E-35
8928	ATP5J_RAT	P21571	ATP synthase coupling factor 6	8921.55	8921.55	0	8927.06		1.7E-52
10918	DLRB1_RAT	P62628	Dynein light chain roadblock-type 1	10909.71	10909.72	−1	10916.41	N-term acetylation, oxidation	6.2E-36
12204	ARP19_RAT	Q712U5	cAMP-regulated phosphorylation 19	12196.24	12196.26	−2	12203.67	N-term acetylation	2.3E-13
14428	Q6MGC4_RAT	Q6MGC4	H2-K region expressed gene 2, rat orthologue	14417.76	14417.73	2	14426.26	N-term acetylation, water adduct	3.1E-24

These proteins were all identified by means of top-down tandem MS sequencing on a nanoLC-ESI-LTQ-Orbitrap Elite system.

## Results

**Figure 1:**
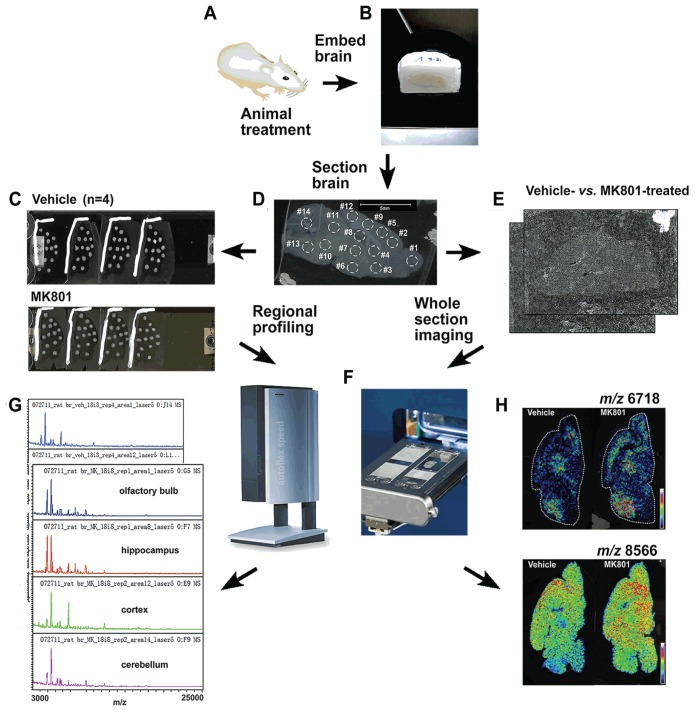
An overall strategy for mapping proteins in brain tissues by combining MS profiling and imaging. The animals treated with MK801 were sacrificed and handled pairwise (A). The brains were removed and embedded in gelatin (B) followed by sectioning (C). Four sections from each brain were used and 14 regions as annotated in (C) from each section were profiled (D). An entire section was imaged from one brain (E). Four pairs of brains (vehicle *vs.* MK801) were profiled and imaged (N = 4), respectively, on a MALDI-TOF/TOF instrument (Bruker autoflex III) (F). The profiling spectra were compared pairwise for each specific region (G), and the imaging data were compared as well (H).

In this study, we used a combined profiling and imaging strategy as illustrated in **Figure 1** to identify the differentially expressed proteins in the vehicle and MK801 treated rat brains. Profiling involves manually depositing matrix onto the regions of interest of the brain section and analyzing the section region by region, whereas imaging requires the whole tissue section to be coated with a homogenous matrix layer and scanned according to a predefined array.

### *In situ* Profiling of the Vehicle- and MK801-treated Infant Rat Brains

**Figure 2:**
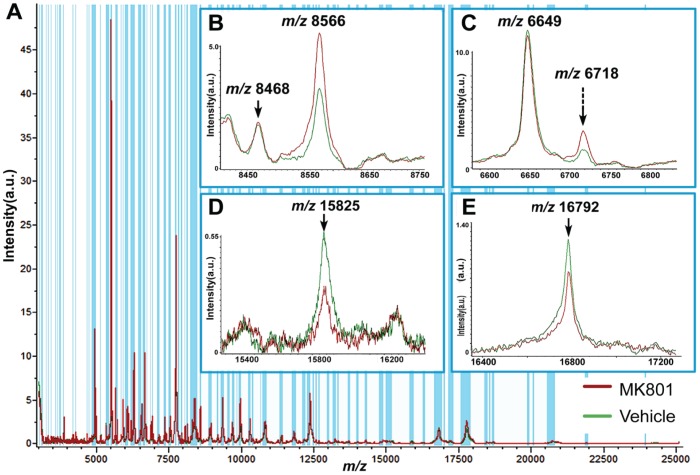
Regional profiling of the rat brain sections treated with vehicle and MK801. (A) Representative overall profiling spectra averaged from 14 regions of 4 serial sections from each animal (N = 4, two classes, i.e. vehicle-treated and MK801-treated rats as displayed in green and red, respectively). (B)–(E) are the zoomed-in spectra of several ions displaying differential expression levels in the two groups. The blue shades indicate the peaks picked by ClinProTools (signal to noise threshold higher than 5 in the average spectra) and included for statistical comparison between the two classes.

For high-throughput profiling, four sections from each of the four pairs of sex-matched P10 infant rat brains that had undergone treatment with saline as the control group (N = 4) and MK801 as the treated group (N = 4) were compared. Each section was manually profiled with fourteen spots representing the corresponding major anatomical structures in a rat brain sagittal section. Specifically, the fourteen major regions were divided based on rat brain atlas (www.brainmaps.org) as designated in **Fig. 1C**, including olfactory bulb as region #1, accumbens nucleus (Acb) as region #3, cortex as region #2, 5, 9 and 12, corpus callosum (cc) as region #4, hypothalamus (Hypo) as region #6, thalamus (TH) as region #7, hippocampus (Hippo) as region #8, medulla as region #10, colliculi as region #11, pons as region #13 and cerebellum as region #14. Spectra produced from profiling the fourteen brain regions clearly differentiate the anatomical regions as previously reported. More importantly, the MS profiles of proteins from each of the regions were compared between infant rat brains treated with vehicle and MK801 with statistical support provided by ClinProTools. The differences of peaks were evaluated by *m/z*, signal intensity and p value. Although most of the masses found in the two classes resemble each other, the overlaid representative spectra from the two classes displayed differential abundance levels of several peaks in the control as the green trace and the treated samples as the red trace shown in **Fig. 2A**. The enlarged spectra are displayed in **Fig. 2B–E**. **Fig. 2B** displays the peaks at *m/z* 8468 and 8566. In this panel, the former peak exhibited relatively the same abundance between the two groups, whereas the latter was significantly upregulated in the averaged spectra acquired from MK801-treated rat brains. **Figure 2C** shows another peak at *m/z* 6718, the abundance of which was also greatly increased in the MK801 treated group, nevertheless, the peak at *m/z* 6649 was detected at almost the same intensity in the two groups. In contrast with **Fig. 2B and 2C**, **Fig. 2D** and **Fig. 2E** exhibit two ions at *m/z* 15825 and *m/z* 16792 that were both down-regulated in the MK801-treated group. Technical replicates within each animal group were averaged and the mean values of each group were used to perform statistical test. More proteins differentially expressed in the control compared with the treated samples are summarized in **Table 1** with the information of observed *m/z*, PTTA between the two groups and their relative abundance ratio. The low *P* values obtained from PTTA suggest that the observed differences of the individual peaks' abundances are statistically significant and are not due to coincidence. This result demonstrates that the peaks as detailed in Table 1 can be used to differentiate the control from the treated group. Based on previous MALDI-MS literature [29], [41], [47] and database search in UniProt, several peaks that exhibited differential abundance levels after MK801 treatment were mass-matched to proteins with a mass error tolerance of ±4 Da. For example, the proteins displayed higher abundances in the treated group were assigned as ubiquitin at *m/z* 8566 as shown in **Figure 2B** and PEP-19 at *m/z* 6718 as displayed in **Figure 2C**, whereas the protein at *m/z* 6649 that displayed the same abundance level following the treatment was assigned as cytochrome c oxidase subunit 6a-L as in **Fig. 2C**. In contrast, the protein that exhibited decreased abundance in the MK801-treated rat brains was identified as calmodulin with *m/z* 16792 in **Fig. 2E**. Although MALDI-MS has demonstrated its capability of providing accurate *m/z* of the protein species present in rat brain sections for identification, the identities of the molecules that have not been reported, such as the peaks at *m/z* 8468 and 15825 in **Fig. 2B and 2D**, could not be assigned due to the lack of sequence-specific fragmentation information. A comprehensive strategy that combines MALDI-MS with high resolution top-down MS/MS sequencing was employed for protein identification and will be discussed in the following section.

### Mapping the Spatial Distribution of Peaks of Differential Expression Patterns from the Vehicle- and MK801-treated Infant Rat Brains

**Figure 3:**
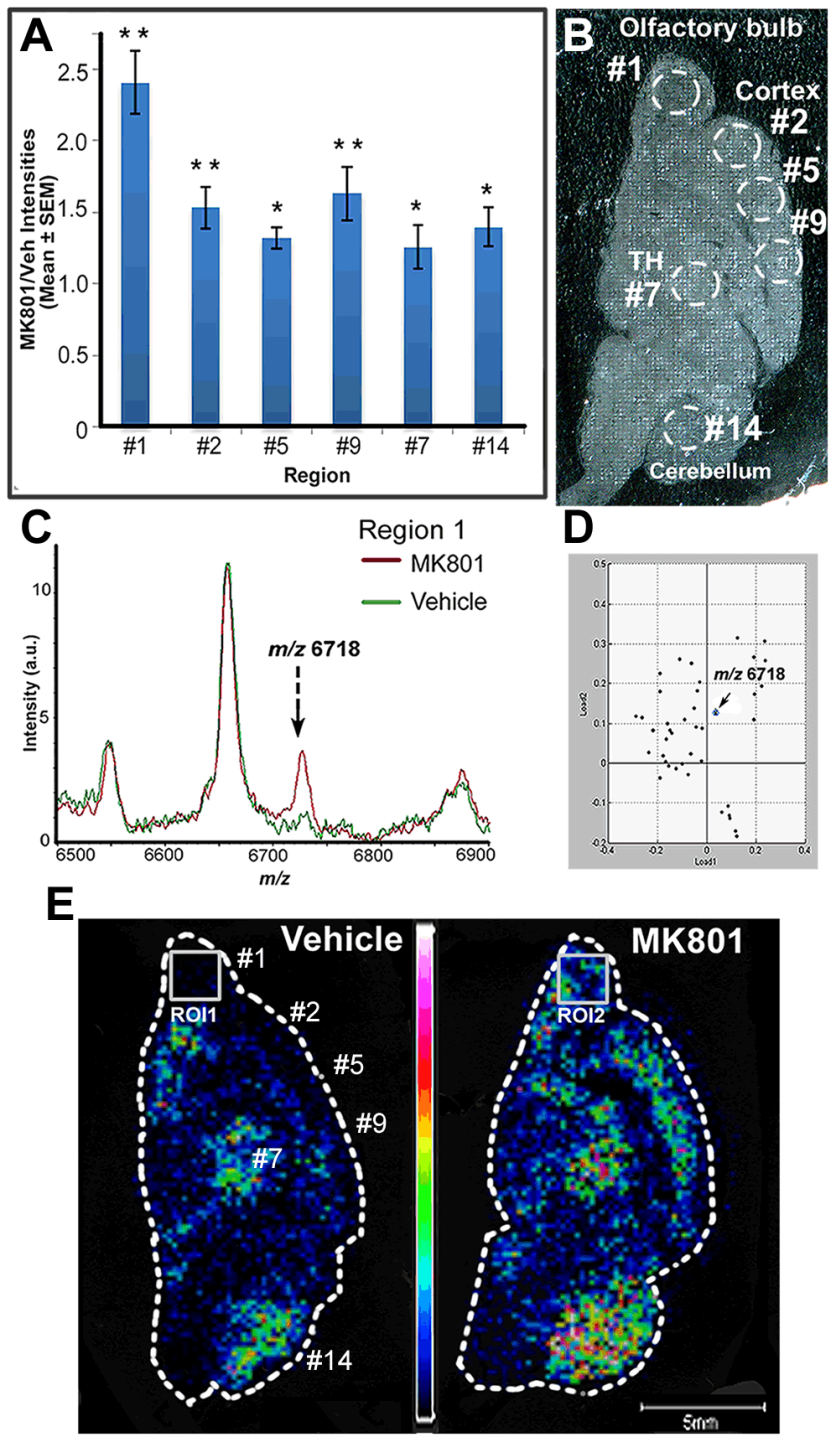
Elevated level of the *m/z* 6718 ion quantified by the strategy combining MS profiling and imaging. A) The ratios of the intensities of the ion at *m/z* 6718 in the profiling spectra of specific regions, regions #1, #2, #5, #9, #7 and #14, where the ion was concentrated. The averaged intensities and p-values were calculated by ClinProTools and manually confirmed. *, p<0.05; **, p<0.01, unpaired student's t-test. (B) An optical image of a rat brain section washed after MSI experiments. (C) The *m/z* 6718 ion was up-regulated in the averaged MSI spectrum of the olfactory bulbs, ROI 1 and ROI2, of the rat brain section treated with vehicle and MK801 as shown in (E). (D) A loading plot of ROI 1 and ROI 2 analyzed by ClinProTools. The *m/z* 6718 ion was shown to contribute significantly in differentiating the two ROIs in Load 2. (E) MS images of the ions at *m/z* 6718 for the vehicle and the MK801-treated rat brain sections. The *m/z* 6718 ion was more concentrated in the olfactory bulb, cortex, thalamus (TH) and cerebellum regions in the MK801-treated section compared to the vehicle-treated one.

Although the profiling strategy provides high throughput information regarding regional differences, MSI allows for mapping of the ions of interest in the entire rat brain section with much higher spatial resolution than regional profiling. Overall, the average spectra for the imaged brain sections of MK801 treated rats exhibited great similarities to those of vehicle-treated rats, agreeing well with the average profiling spectra. Nevertheless, the differential features highlighted in the profiling approach were confirmed through MSI using one rat brain section from each group as depicted in **Fig. 1E**. Additionally, statistical software also aids in validating the potential biomarkers that displayed differential expression levels in the vehicle and treated groups. For example, the profiling spectra showed that the ion at *m/z* 6718 exhibited significant up-regulation by 1.8 fold in the MK801-treated group compared to the vehicle-treated one as in **Table 1**. More specifically, regions #1, #2, #5, #9, #7 and #14 displayed the most drastic changes after the treatment (**Fig. 3A**). **Figure 3B** is an optical image of a rat brain section that underwent MSI experiments. As highlighted in **Figure 3B**, region #2, #5 and # 9 correspond to the frontal and cingulate cortex, and region #1, #7 and #14 are registered to the olfactory bulb, TH and cerebellum, respectively. Among all the regions, olfactory bulb as region #1 exhibited the most prominent changes with a 2.5 fold increase comparing to roughly 1.5 fold as shown by other regions. This observation was confirmed by imaging data as shown in **Figure 3C**. **Figure 3C** shows two MS spectra averaged specifically from ROI 1 and ROI 2 in **Figure 3E**, which correspond to the olfactory bulb regions from the vehicle and the MK801 treated rat brain sections, respectively. The peak at *m/z* 6718 was elevated to roughly 3 fold from the MK801-treated spectrum in comparison to that of a vehicle-treated one, consistent with the profiling results. Moreover, the loading plot shown in **Figure 3D** suggests that the *m/z* 6718 peak contributes significantly to the distinction of the two ROIs, especially in load 2, providing statistical evidence to the differentiating role of this ion. **Figure 3E** displays more straightforward proof by showing the MS images of the *m/z* 6718 ion obtained from the vehicle and MK801 treated section. The difference in color suggested that the *m/z* 6718 ion was significantly more concentrated in the olfactory bulb of the MK801 treated section. The cortex also exhibited explicit difference in the vehicle- and the MK801-treated group. Moreover, the abundance of the *m/z* 6718 ion in TH and cerebellum were also increased as indicated by the MS images. The differential expression levels of *m/z* 6718 in the vehicle and MK801-treated rat brains were reproducibly proven by the integration of profiling and imaging data, demonstrating consistency of our combined strategy.

**Figure 4:**
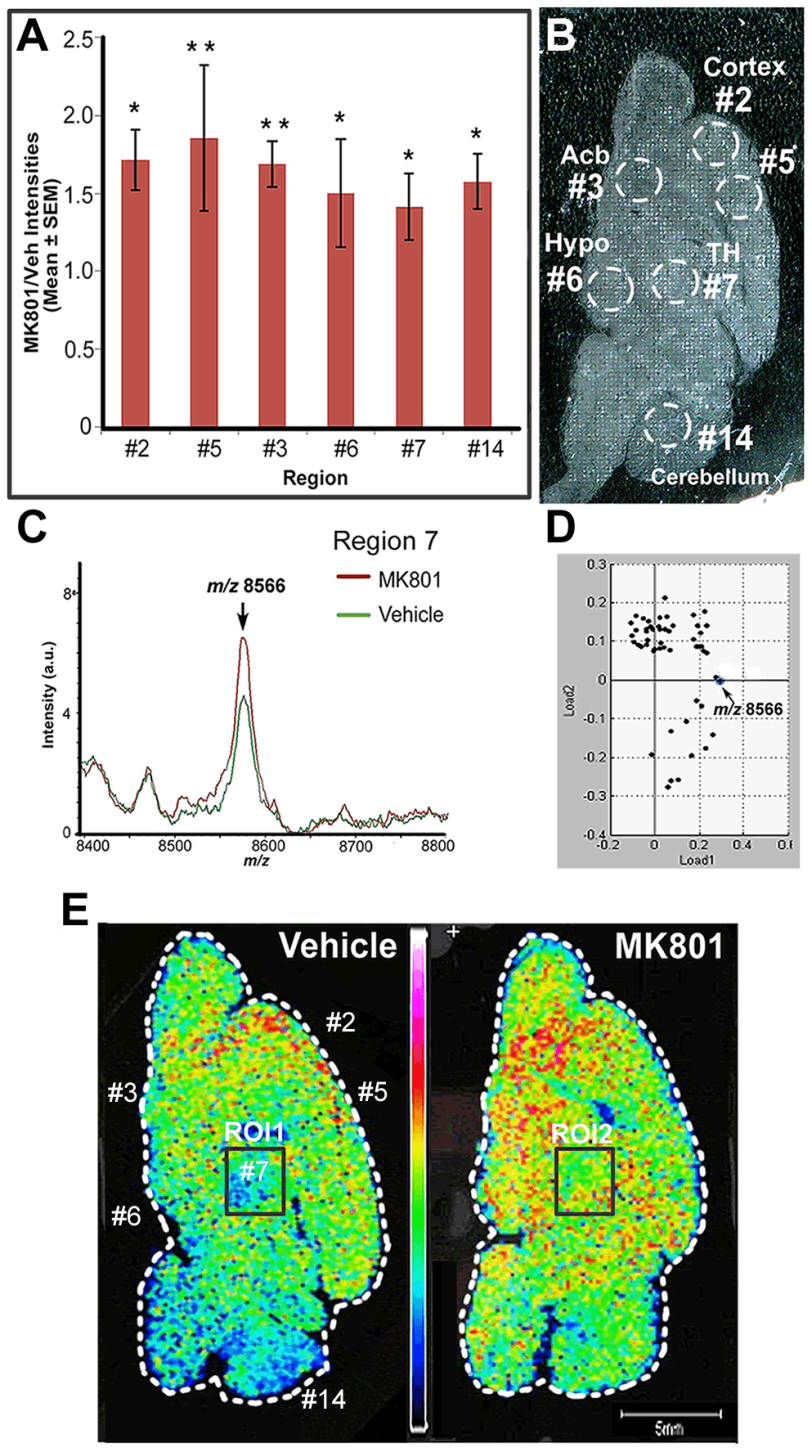
Elevated level of the *m/z* 8566 ion quantified by the strategy combining MS profiling and imaging. A) The ratios of the intensities of the ion at *m/z* 8566 in the profiling spectra of specific regions, regions #2, #5, #3, #6, #7 and #14, where the ion was concentrated. *, p<0.05; **, p<0.01, unpaired student's t-test. (B) An optical image of a rat brain section washed after MSI experiments. (C) The *m/z* 8566 ion was up-regulated in the averaged MSI spectrum of the thalamus (TH) regions, ROI 1 and ROI2, of the rat brain section treated with vehicle and MK801 as shown in (E). (D) A loading plot of ROI 1 and ROI 2 analyzed by ClinProTools. The *m/z* 8566 ion was shown to contribute significantly in differentiating the two ROIs in Load 1. (E) MS images of the ions at *m/z* 8566 for the vehicle and the MK801-treated rat brain sections. The *m/z* 8566 ion was more concentrated in the frontal cortex, accumbens nucleus (Acb), thalamus (TH) and cerebellum regions in the MK801-treated section compared to the vehicle-treated one.

Other than the *m/z* 6718 ion, the abundance of the peak at *m/z* 8566 in the MK801-treated group was elevated by 1.6 fold compared to the vehicle-treated one. Profiling spectra of regions #2, #5, #3, #6, #7 and #14 displayed the most distinguishable changes in expression levels of the *m/z* 8566 ion as shown in **Fig. 4A**. As annotated in **Figure 4B**, regions #2 and #5 are registered as cortex; regions #7 and #14 correspond to TH and cerebellum, respectively; and regions #3 and #6 are assigned as Acb and Hypo. Accordingly, the intensity of the *m/z* 8566 ion in the MS spectra of the TH regions from the MK801-treated rat was elevated by around 1.4 fold comparing to the vehicle-treated one as indicated in **Figure 4C**, which agreed well with the increase displayed in **Figure 4A**. The spectra were averaged specifically from the TH regions of the vehicle and MK801-treated rat brain sections, which were designated as ROI 1 and ROI 2, respectively. **Figure 4D** shows the statistical loading plot of the two ROIs, indicating that the *m/z* 8566 ion contributes most significantly to differentiate the TH regions of the two groups in Load 1. The statistical evidence was further supported by the MS images displayed in **Figure 4E**. Acb as region #3, Hypo as region #6 and cortex as region #2 and #5 all showed significant increase in relative abundance of the *m/z* 8566 ion in the image obtained from the MK801-treated section compared with the vehicle-treated section, as indicated by the color scale in **Figure 4E**. TH as region #7 and cerebellum as region #14 also exhibited noticeable increase of concentration in the MK801-treated section compared with the vehicle one. In summary, the utilization of the MALDI-MS profiling and imaging techniques was exemplified by the differentiating peaks at *m/z* 6718 and 8566, and have been applied to discern the proteins that changed significantly following MK801 treatment due to the reproducibility and consistency of the combined strategy.

### Protein Identification

**Figure 5:**
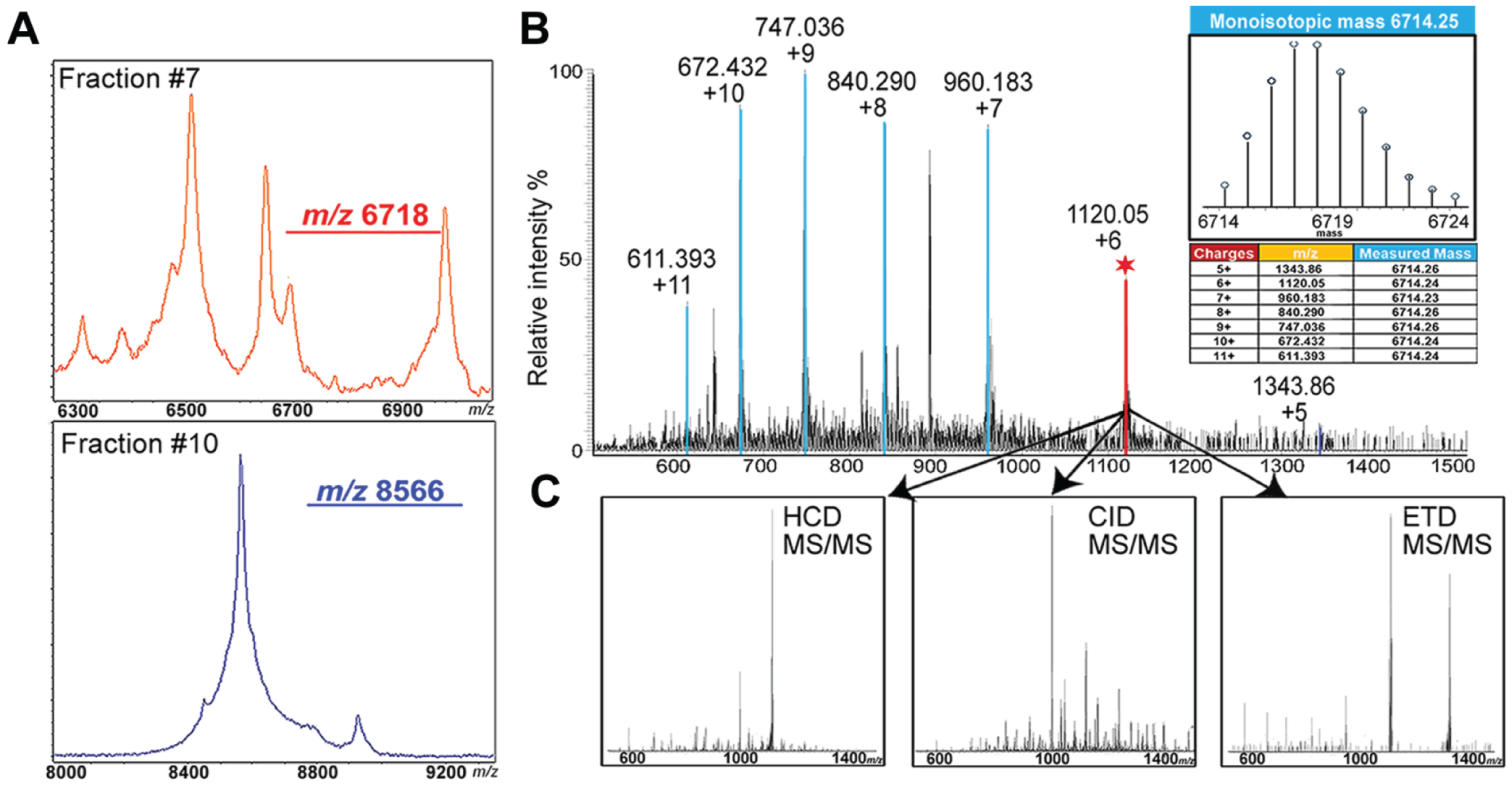
The workflow to identify the proteins that displayed changes in the MK801-treated samples. (A) MALDI-MS screening spectra of the HPLC fractions that contained the concentrated proteins of interest at *m/z* 6718 and 8566. (B) Representative MS spectrum acquired from a HPLC fraction containing the *m/z* 6718 ion on an ESI-Orbitrap Elite system, which provides ultra-high mass resolution and accuracy. The insert is the deconvoluted spectrum averaged from several multiply charged ion forms as highlighted in blue and red. (C) HCD, CID and ETD tandem MS fragmentation spectra of the particular ion at *m/z* 1120.05 (z = 6) highlighted in red.

**Figure 6:**
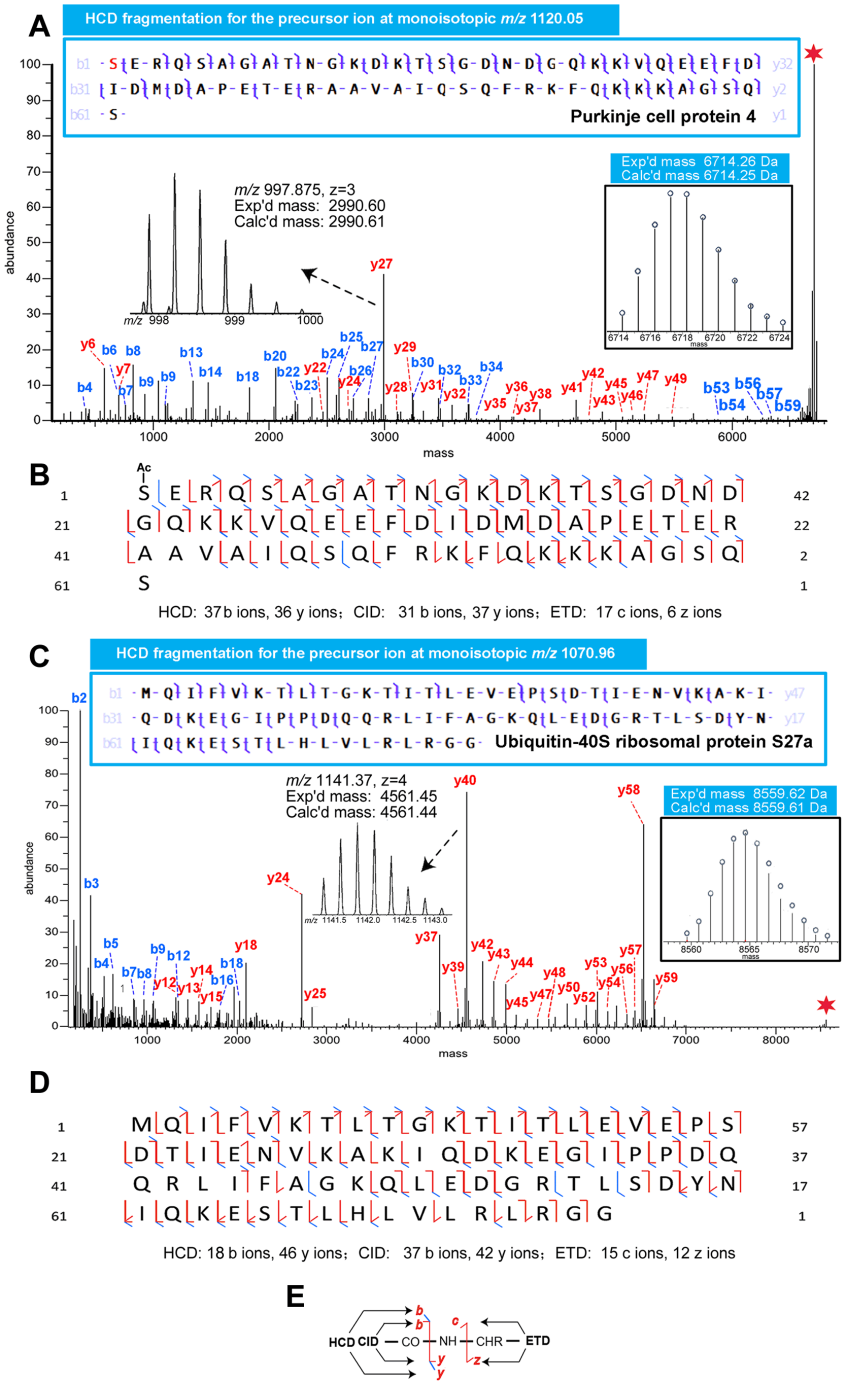
Fragmentation spectra and maps of the *m/z* 6718 and 8566 ions by HCD, CID and ETD. (A) HCD fragmentation spectrum of the *m/z* 6718 ion at monoisotopic *m/z* 1120.05 (z = 6). The ion at *m/z* 1120.05 (z = 6) was assigned as PEP-19 based on accurate mass and fragmentation pattern obtained on Orbitrap Elite. The boxed insert on the right is the deconvoluted spectrum of the *m/z* 1120.05 (z = 6) ion. The original HCD tandem MS spectrum containing the y_27_ fragment ion is also zoomed-in as an insert on the left. (B) Fragmentation map of PEP-19 by three fragmentation techniques, HCD, CID and ETD. (C) HCD fragmentation spectrum of the *m/z* 8566 ion at monoisotopic *m/z* 1070.96 (z = 8). The ion at *m/z* 1070.96 (z = 8) was assigned as ubiquitin based on accurate mass and fragmentation pattern obtained on Orbitrap Elite. The boxed insert on the right is the deconvoluted spectrum of the *m/z* 1070.96 (z = 8) ion. The original HCD tandem MS spectrum containing the y_40_ ion is also enlarged as an insert on the left. (D) Fragmentation map of Ubiquitin by HCD, CID and ETD. The number of the fragment ions produced by each of the fragmentation techniques is listed below the sequence. (E) An illustration of how the fragment ions of HCD (b, y ions), CID (b, y ions) and ETD (c, z ions) are produced and annotated in the fragmentation map is shown in (B) and (D).

Since knowledge about the identity and chemical form of the molecular species of interest is essential to investigate their biological roles, efforts were focused on identifying the peaks that display significantly different expression levels after MK801 treatment. Although the discrimination could be made by combining the imaging and the profiling techniques utilizing MALDI-TOF/TOF, it is significantly more difficult to identify the potential proteins of interest via MALDI-MS alone due to limited fragmentation obtainable from the singly charged high-mass ions produced by MALDI-MS. The initial step of identification was fulfilled by the conventional fractionation, which helped eliminating ion suppression effects, reducing sample complexity and thus increasing the dynamic range of the analysis. The rat brains were pooled and fractionated in RP HPLC to concentrate the targeted proteins and reduce the complexity arising from high abundance proteins of tissues. All fractions were then screened on MALDI-TOF/TOF with the aim to ascertain if the proteins of interest observed on MALDI-MS from the brain sections were extracted by tissue homogenization and purified by crude fractionation. As shown in **Fig. 5A**, the peaks of *m/z* 6718 and 8566 were detected at high abundance in fraction #7 and #10, respectively. The fractions containing the targeted proteins selected via MSI were then analyzed on a nanoLC-ESI-Orbitrap Elite system. The accurate masses provided by this high-end instrument were then deconvoluted, and the averaged masses were compared to the masses detected on MALDI-TOF/TOF for mass-matching. **Fig. 5B** shows a representative MS spectrum, which contains several multiply charged ions, including *m/z* 611.392 (z = 11), *m/z* 672.432 (z = 10), *m/z* 747.036 (z = 9), *m/z* 840.290 (z = 8), *m/z* 960.183 (z = 7), *m/z* 1120.05 (z = 6) and *m/z* 1343.86 (z = 5). After spectrum deconvolution performed by software Xtract 3.0, these ions were all attributed to the molecule with monoisotopic mass at 6714.25 Da. Moreover, the well-resolved isotopic distribution in the insert of **Fig. 5B** suggests its neutral average mass at 6717 Da, which could be matched with the singly-charged *m/z* 6718 peak generated by MALDI-MS. The mass discrepancy between the peaks observed on TOF/TOF and Orbitrap further demonstrates the different resolution provided by two instruments. Followed by mass-matching with the targeted proteins, the selected ions were then subjected to top-down high resolution tandem MS, using a complementary suite of fragmentation techniques: HCD, CID and ETD. **Fig. 5C** displays the multiple fragmentation spectra arising from the *m/z* 1120.05 (z = 6) ion using these various techniques. The rich sequence-specific fragment ions produced by the complementary fragmentation techniques were used to search the rat protein database with ProSightPC 3.0. Based on the ultra-high mass resolution (120,000 in MS^1^ and 60,000 in MS^2^ at *m/z* 400) and accuracy (mass error smaller than 5 ppm) together with the high quality MS/MS spectra, we identified the peak at *m/z* 6718 on the MALDI-TOF/TOF as N-term acetylated PEP-19 and *m/z* 8566 as ubiquitin-40S ribosomal protein S27a (Ubiquitin). The best fragmentation spectra corresponding to the two ions are displayed in **Fig. 6A and 6C**, validating the previous identifications assigned based on masses. The insert in **Fig. 6A** is the deconvoluted spectrum of the precursor ion at *m/z* 1120.05, showing its monoisotopic mass of 6714.25 Da. The original tandem MS spectrum is enlarged to exhibit the most abundant fragment ion y_27_ at *m/z* 997.875, which demonstrates the high mass accuracy and resolution of the Orbitrap Elite system we employed in this study. In the inserts of **Fig. 6C**, the precursor ion at *m/z* 1070.96 with a monoisotopic mass of 8559.61 Da gave rise to an abundant ion y_40_ at *m/z* 1141.37. The deconvoluted mass of this ion is calculated as 4561.45 Da, exhibiting merely a 0.01 Da mass error from the theoretical value. The high-quality tandem MS spectra led to high-confidence protein identification, facilitating future mechanistic studies on these proteins and related disease.

Inevitably, the same protein forms of high abundance were repeatedly selected for fragmentation within a single run. Therefore, increased cleavage site and improved fragmentation coverage were observed for all of the three fragmentation techniques. For example, the ion shown in **Fig. 6B** corresponds to acetylated PEP-19, identified with an E-value of 1.5E-77. Among all the fragmentation methods, HCD provided the most complete coverage for PEP-19 and enabled accurate localization of the acetylation site at the Serine of N-terminal; CID delivered comparable fragment ions in comparison to CID; whereas ETD produced ∼1/3 of the number of the HCD fragment ions. As for ubiquitin, CID produced the most efficient fragmentation covering majority of the possible fragmentation sites, and CID produced slightly fewer fragment ions as displayed in **Fig. 6D**. Nevertheless, ETD again displayed merely ∼1/3 of the fragment ions obtained from either HCD or CID. **Fig. 6E** illustrates the product ions generated by CID, HCD and ETD. ETD is a radical driven fragmentation technique and cleaves at N-Cα bond, which is distinct from those of HCD and CID. Therefore, ETD is complementary to HCD and CID fragmentation methods. This has been validated by comparing the fragmentation patterns produced by the three fragmentation techniques as shown in **Fig. 6B and 6D**. The utilization of all three fragmentation techniques significantly improves the coverage of the cleavage sites from the targeted proteins. The differentiating proteins displayed by MALDI-MS and identified by top-down tandem MS sequencing were listed in **Table 1** with details presented in **Table S1**, whereas the proteins that displayed relatively the same abundance among groups via MALDI-MS yet identified by top-down sequencing were summarized in **Table 2**. A number of proteins, such as mitochondria import inner membrane translocase at *m/z* 10283, adenylate cyclases type 10 at *m/z* 9193, polyubiquitin at *m/z* 8468 and superoxide dismutase [Cu-Zn] at *m/z* 15825 were reported from the rat brain in a MALDI-MS setting for the first time. The identification of these proteins could provide valuable *m/z* information for other researchers that rely on MALDI-MSI to discover protein biomarkers. A full list of identified proteins and corresponding fragment ions' annotation based on high resolution top-down MS sequencing are detailed in **Table S1, S2 in File S1** and **File S2(top-down MSMS fragmentation)**.

## Discussion

### A Combined Profiling and Imaging Strategy for Biomarker Discovery and Screening

Our MALDI MS-based strategy combines MS profiling and imaging techniques with the goal to screen for changes in the distribution and relative abundance of proteins in the brains of rats that had experienced early developmental exposure to the NMDA antagonist MK801 on postnatal days 2 and 4. The profiling approach, which consists of manually depositing matrix on predefined substructures on a rat brain section, clearly highlights its advantages of speed, robustness and high reproducibility. Nevertheless, profiling also has the limitations that low spatial resolution is obtained for each region of interest. Alternatively, laser capture microdissection (LCM) technique is capable of isolating specific cell populations of interest from target tissue specimens, and has been combined with MSI for biomarker discovery by comparing spectra obtained from disease cells and normal cells [48], [49]. Nonetheless, the administration of NMDA antagonists triggered cell apoptosis of great heterogeneity in developing brains as visualized by TUNEL [6]. Therefore, the LCM-captured cells might not be representative of all of the regions that might be perturbed during MK-801 treatment. Furthermore, molecular information of the un-profiled regions is lost. Complementary to rapid profiling by MS, the imaging approach delivers high spatial resolution MS images, allowing for further investigation of structural detail of the entire rat brain section. The utilization of software ClinProTools aids in raw data processing and statistical analysis to filter the spectral features that displayed significant changes in the MK801 and vehicle treated samples. Relying on the combined profiling and imaging strategy, elevated levels of ubiquitin, thymosin-β 4, PEP-19, acyl-CoA-binding protein subunits, cytochrome c oxidase subunits, mitochondria import inner membrane translocase and adenylate cyclase type 10 were detected in the MK801-treated rat brains when compared to the vehicle-treated ones in the profiling spectra of selected brain regions. Moreover, MALDI-MSI affirmed increased abundance of these proteins by delivering high spatial resolution and high throughput MS images directly from the rat brain sections obtained from rats subjected to MK801 treatment. On the other hand, proteins that show decreased relative abundance levels in the MK801 treated rats were also observed, such as ATP synthase-coupling factor 6, ProSAAS, secretogranin, calmodulin and Superoxide dismutase [Cu-Zn]. Intriguingly, the observation of these discriminating peaks in the MK801- and vehicle-treated rats might suggest involvement of the respective proteins in the pathological mechanisms of observed neurological deficits or in reparative processes. In contrast, the detection of proteins that displayed the same expression levels in the MK801 treated rats might imply that proteins from the treated subjects function normally as in the control group.

The spatial distribution of these differentially expressed proteins, determined by MS profiling and imaging techniques, corroborated with previous literature reporting massive apoptosis in the frontal and cingulate cortex following MK801 treatment of infant rats [6]. As shown in **Figure 3E** and **4E**, significant differences could be observed in frontal and cingulate cortex for both ions, indicating that NMDA receptor blockade induced by MK801 during development might have led to severe neuronal loss in this region and triggered significant response of brain tissue in the form of protein expression level changes. Selective vulnerability of brain regions to MK801-induced imbalance could be attributed to a high level of NMDA receptors on neuronal populations within those regions [50]. The significant difference observed in the Acb region could also be attributed to a high concentration of NMDA receptors in Acb within the basal ganglia. However, receptor density cannot solely account for neuronal vulnerability. The NMDA receptor density together with additional factors, such as stage in synaptogenesis during treatment and the subunit composition of the NMDA receptor complex, combinatorially determine the degree of neuronal sensitivity [6]. This probably explains the significant changes observed in other regions, such as thalamus, cerebellum and olfactory bulb as shown in **Figure 3E** and **4E**.

### MALDI-MS Combined with High Resolution Top-down Tandem MS for Protein Identification

The subsequent step after biomarker screening is to unequivocally identify the protein targets of interest. We employed an MSI approach in combination with top-down tandem MS identification for this purpose. In a top-down proteomics experiment, protein identification is performed through intact mass measurement followed by tandem MS sequencing with high resolution high mass accuracy FTMS-based platform. In comparison to bottom-up tandem MS sequencing, top-down tandem MS approach directly analyzes intact proteins [51] and best preserves the biological information observed by MALDI-MS [52]. It avoids the tedious mass correlation between intact protein masses observed on MSI and digested peptide masses detected by ESI-MS/MS. Moreover, it provides unique advantages in assessing protein modifications, such as PTMs and sequence variants, present *in vivo* with no prior knowledge [53], [54]. The high-end instrument employed in this study offers superior mass accuracy in MS and MS/MS [55], which is highly advantageous for precise characterization of PTMs, and ultra high mass resolution, exemplified by the well-resolved whole protein isotopic distribution as shown in **Fig. 6A and 6C**. Combining with a standard fractionation step, we further purified the proteins of interest and unambiguously determined their identities by top-down tandem MS sequencing. Most of the protein targets observed by MALDI-MS were verified by the top-down approach and reliably mapped with full sequence coverage and PTMs. Other proteins were detected and identified, and are shown in the **Table S1, S2 in File S1**. The innovative protein identification strategy applied in this study, by combining the advantages of MALDI-MSI and top-down high resolution tandem MS, enables the identification of potential protein biomarkers for neuropsychiatric syndromes which result from excitatory/inhibitory neurotransmission imbalance in early development.

The ion signal at *m/z* 16792 in this study was identified as calmodulin and was also found to be N-terminally acetylated. In contrast to those upregulated proteins, MK801 treatment decreased the relative intensity of calmodulin in the rat brain compared to the vehicle-treated rats. Calmodulin is a major Ca^2+^-binding protein in cells, where it complexes with Ca^2+^ and activates several types of intracellular enzymes, including kinases, phosphatases and adenyl cyclases [56]. In its Ca^2+^ free form, calmodulin can also regulate the function of various proteins, like actin-binding and cytoskeletal proteins [57], making it essential in the regulation of cell structure. Failure of neuronal calcium homeostasis has been associated with cytotoxic events in the aging process and the pathogenesis of neurodegenerative diseases [58], since Ca^2+^/calmodulin signaling is involved in controlling processes such as muscle contraction, neurotransmitter release, transcriptional regulation, and cell death [59], [60]. More intriguingly, calmodulin has also been linked to the regulation of ion channels coupled to NMDA receptors [56].

Several intracellular peptides that contained an IQ motif were bestowed a unique ability to bind calcium-poor calmodulin, such as neuromodulin, neurogranin and PEP-19 [56]. PEP-19 can modulate Ca^2+^/calmodulin homeostasis by altering the calcium-binding dynamics of free calmodulin and calmodulin bound to other target proteins. Interestingly, expression level changes of PEP-19 have been linked to several neurodegenerative conditions, such as Parkinson's disease [29], Alzheimer's disease [61] and Huntington's disease [32].javascript:void(0); Among the biological function studies of PEP-19, a study showing that PEP-19 can inhibit apoptotic cell death due to UV irradiation or treatment with staurosporine is of special interest [56]. Hence, the upregulation of PEP-19 in the MK801 treated samples could contribute to neuronal reparative processes.

Other than Ca^2+^/calmodulin signaling, the ubiquitin pathway might also be involved in the molecular pathology of NMDA receptor antagonist neurotoxicity in developing rat brains given the increased abundance of ubiquitin observed in the MK801-treated group. Aberrations in the ubiquitin system have been linked with neurodegenerative diseases, such as Parkinson's disease [62], Alzheimer's disease [61], [63], Huntington's disease [64], Down's Syndrome [65] or progressive supranuclear palsy [66]. Recently, ubiquitin gene networks expressed within the central nervous system have been postulated to contribute to the genetic susceptibility to ASD [67]. Previous literature also suggested that accumulation of ubiquitin could potentially result in dominant inhibition of the ubiquitin–proteasome system, leading to insufficient degradation of toxic proteins with neuropathological consequences.

The abundance of thymosin beta-4 was also elevated in rat brains subjected to MK801 treatment. Thymosin beta 4 (Tβ_4_) detected at *m/z* 4964 with N-term acetylation is a major actin monomer-sequestering molecule in mammalian cells. It has various biological functions, including regulation of inflammatory chemokines and cytokines, cellular migration, blood vessel formation and apoptosis [68]. It has been shown to prevent cell death from infections in the eye [69], loss of blood supply in the heart after myocardial infarction [70] and toxins in nerve cells [71]. For example, administration of thymosin beta has been shown to prevent the loss of hippocampal neurons after kainic acid treatment [71]. The significance of elevated abundance of Tβ_4_ following MK801 treatment is unknown. It may be speculated that there is possible involvement of Tβ_4_ in reparative processes, but its value as a potential therapeutic target ought to be evaluated in future research.

In this study, our results also revealed an intriguing increase of three cytochrome c oxidase (COX) subunits, i.e. the smallest subunit 7c at *m/z* 5486, the subunit 6c at *m/z* 8368 and the largest subunit 6b at *m/z* 9979, in the MK801 treated group, although the subunit 6c was not confirmed by top-down MS/MS spectra. The COX subunits reported here are mitochondria respiratory chain (MRC) components, involved in electron transfer to oxygen [33]. COX is known to modulate apoptosis [72], [73]. Previous studies have shown that an induction of MRC proteins like COX precede apoptosis induced by multiple stimuli. Specifically, increased expression of COX mRNAs and proteins were observed in apoptotic model systems such as Jurkat cells treated with camptothecin [74], breast cells treated with teniposide [74] and apoptotic epithelial cancer cells induced by chemical BMD188 [75]. The increased synthesis of MRC proteins, possibly representative of a more global mitochondrial activation response, is suggested to be responsible for subsequent disruption of MRC functions and ultimately cell death in response to apoptotic stimuli [72]. The up-regulation of COX subunits detected here could possibly be explained by this mechanism.

**In conclusion**, we have demonstrated that MALDI-MSI is capable of mapping the spatial distribution and relative abundance changes of multiple proteins simultaneously and directly from rat brain sections. The profiling strategy is fast, robust, and highly reproducible for producing proteome “snapshots” of the anatomical regions of interest in the infant rat brain sections. Complementary to this, the MSI technology enables more detailed molecular mapping with enhanced spatial resolution while increasing instrument time as a compromise. A combination of the two techniques allowed localization and assessment of the relative abundances of the biomolecules expressed in the MK801-treated and control samples with relatively high spatial resolution and reproducibility. The subsequent high mass spectral resolution top-down tandem MS approach allowed protein identification through accurate mass measurement of intact proteins followed by complementary fragmentation for tandem MS analysis. This hybrid approach showcases the unique abilities of MSI in the discovery of potential disease biomarkers. Relying on such a MSI-based platform, we have observed 22 proteins that exhibited differential relative abundance level changes upon NMDA receptor antagonist treatment such as PEP-19, ubiquitin, calmodulin, COX subunits. These potential biomarkers of excitatory/inhibitory neurotransmission imbalance-induced neurodevelopmental disorders reported in this pilot study are involved in Ca^2+^/calmodulin homeostasis, ubiquitin–proteasome system and mitochondria activation, possibly playing significant roles in brain neuron apoptosis or reparative processes. Collectively, this study opens up new avenues for future applications in the field of biomarker discovery and for mechanistic investigations delineating pathophysiology of neurodevelopmental disorders.

## Supporting Information

File S1 Method S1, On-line Top-down MS/MS on Nano-LC-ESI-LTQ-Orbitrap Elite. Table S1, Details of differentially-expressed rat brain proteins observed on MALDI-TOF/TOF and identified by top-down MS/MS sequencing. Table S2, Details of proteins identified merely by top-down MS/MS sequencing on nanoLC-ESI-LTQ-Orbitrap Elite.
(DOC)

File S2 **Top-down MSMS fragmentation.** Fragment ions assignments of all the proteins identified by top-down MS/MS sequencing listed in the order as in **Table 1**/**Table S1**, **Table 2** and **Table S2**.
(XLS)

File S3 **Top-down MSMS spectra.** Raw top-down MS/MS spectra and fragmentation maps of all the proteins identified on nanoLC-ESI-LTQ-Orbitrap Elite listed in the order as they are shown in **Table 1**/**Table S1**, **Table 2** and **Table S2**.
(PDF)
