# Supplementary material for: Top-Down Proteomics with Mass Spectrometry Imaging: A Pilot Study towards Discovery of Biomarkers for Neurodevelopmental Disorders
Source: PLoS One. 2014 Apr 7;9(4):e92831. doi: 10.1371/journal.pone.0092831 (PMC3978070; doi:10.1371/journal.pone.0092831)
Supplement: File S1 — Method S1, On-line Top-down MS/MS on Nano-LC-ESI-LTQ-Orbitrap Elite. Table S1, Details of differentially-expressed rat brain proteins observed on MALDI-TOF/TOF and identified by top-down MS/MS sequencing. Table S2, Details of proteins identified merely by top-down MS/MS sequencing on nanoLC-ESI-LTQ-Orbitrap Elite. (DOC) [file pone.0092831.s001.doc]

**Supporting Information for**

**Top-down proteomics with mass spectrometry imaging: a pilot study towards discovery of biomarkers for neurodevelopmental disorders**

Hui Ye^1,2,3^, Rakesh Mandal^4^, Adam Catherman^5^, Paul M. Thomas^5^, Neil L. Kelleher^5^, Chrysanthy Ikonomidou^4*^, Lingjun Li^3,6*^

^1^State Key Laboratory of Natural Medicines, Key Lab of Drug Metabolism and Pharmacokinetics, ^2^Department of Pharmaceutical Analysis, China Pharmaceutical University, Nanjing 210009, PR China,

^3^School of Pharmacy, ^4^Department of Neurology, and ^6^Department of Chemistry, University of Wisconsin Madison, WI 53705, USA, ^5^Proteomics Center of Excellence,

Northwestern University, Evanston, Illinois 60208, USA

^*^corresponding authors

***Correspondences:** Professor Lingjun Li, School of Pharmacy and Department of Chemistry, University of Wisconsin–Madison, 777 Highland Avenue, Madison, Wisconsin 53705-2222, E-mail: [lli@pharmacy.wisc.edu](mailto:lli@pharmacy.wisc.edu); Phone: +1-608-265-8491; Fax: +1-608-262-5345

Professor Chrysanthy Ikonomidou, Department of Neurology and Waisman Center, University of Wisconsin-Madison, 1500 Highland Avenue, Madison, Wisconsin 53705, USA, Email: [ikonomidou@neurology.wisc.edu](mailto:ikonomidou@neurology.wisc.edu); Phone: +1-608-263-5421; Fax: +1-608-263-0412

**SUPPLEMENTARY METHOD S1**

**On-line Top-down MS/MS on Nano-LC-ESI-LTQ-Orbitrap Elite**

The mass spectrometer was operated in a data-dependent mode, performing higher-energy collision induced dissociation (HCD)-MS^2^ (scan 1), collision induced dissociation (CID)-MS^2^ (scan 2) and electron transfer dissociation (ETD)-MS^2^ (scan 3) on each of the Top 3 precursors (selected by intact mass) in a FT-MS precursor scan. The specific conditions for the three fragmentation methods are as follows: HCD: Isolation width: 15 *m/z*, normalized collision energy: 30, activation time: 0.1 ms; CID: Isolation width: 15 *m/z*, normalized collision energy: 41, activation q: 0.4, activation time: 100 ms; ETD: Isolation width: 15 *m/z*, normalized collision energy: 35, activation q: 0.25, activation time: 100 ms.

Data were deisotoped with Xtract using the cRAWler algorithm (ThermoFisher, Bremen, Germany) and searched with a custom 168-core ProSightPC 3.0 cluster using an iterative search tree. The data was first searched in an “absolute mass” method, in which the mass tolerance for precursor ions was set at 200 Da and that for fragment ions was 10 ppm with ∆ m mode on. A minimum match of 5 fragment ions was required. Then a “biomarker” search was performed, in which the mass tolerances for precursor ions and fragment ions are both set at 10 ppm with ∆ m mode off. A minimum match of 4 fragment ions was required.

**SUPPORTING TABLES**

**Table S1.** Details of differentially-expressed rat brain proteins observed on MALDI-TOF/TOF and identified by top-down MS/MS sequencing.

| ***m/z* on MALDI-MS** | **Entry** | **Accession** | **Protein Identity** | **Calc'd Mass (Da)** | **Exp'd Mass (Da)** | **∆ Mass (ppm)** | **Average Mass (Da)** | **PTM** | **E value** |
| --- | --- | --- | --- | --- | --- | --- | --- | --- | --- |
| 6718 | PCP4_RAT | P63055 | PEP-19 | 6714.25 | 6714.26 | -1 | 6718.19 | N-term acetylation | 1.5E-77 |
| 8566 | RS27A_RAT | P62982 | Ubiquitin | 8559.61 | 8559.62 | -1 | 8564.76 |  | 1.8E-37 |
| 4964 | TYB10_RAT | P63312 | Thymosin β-4 | 4960.48 | 4960.49 | -2 | 4963.45 | N-term acetylation | 5.0E-77 |
| 9939 | ACBP_RAT | P11030 | Acyl-CoA-binding protein | 9932.12 | 9932.12 | 0 | 9938.19 | N-term acetylation | 1.2E-44 |
| 9979 | D3ZD09_RAT | D3ZD09 | cytochrome c oxidase polypeptide VI b | 9971.80 | 9971.82 | -2 | 9978.17 | N-term acetylation, 2*disulfide bonds | 1.8E-41 |
| 5486 | B2RYT3_RAT | B2RYT3 | Cytochrome c oxidase subunit 7c | 5481.87 | 5481.87 | 0 | 5485.40 |  | 1.5E-61 |
| 10283 | TIM9_RAT | Q9WV97 | Mitochondria import inner membrane translocase | 10276.08 | 10276.09 | -1 | 10282.66 | N-term acetylation, 2*disulfide bonds | 8.0E-24 |
| 9193 | TIM8B_RAT | P62078 | Adenylate cyclase type 10 | 9187.44 | 9187.45 | -1 | 9193.22 | N-term acetylation, 2*disulfide bonds | 2.3E-25 |
| 8040 | UCRI_RAT | P20788 | Cytochrome b-c1 complex subunit | 8035.38 | 8035.38 | 0 | 8040.30 | N-term acetylation | 4.4E-39 |
| 4936 | TYB10_RAT | P63312 | Thymosin beta-10 | 4933.51 | 4933.52 | -2 | 4936.47 | N-term acetylation | 7.5E-87 |
| 3430 | ATP5J_RAT | P21571 | ATP synthase-coupling factor 6 | 3426.89 | 3426.90 | -3 | 3428.94 |  | 4.3E-13 |
| 16792 | CALM_RAT | P62161 | Calmodulin | 16779.82 | 16779.81 | 1 | 16790.32 | N-term acetylation, unfixed acetylation | 1.3E-10 |
| 3717 | Q5U2U9_RAT | Q5U2U9 | CCR4-NOT transcription complex, subunit 8 | 3713.87 | 3713.88 | -3 | 3716.25 |  | 7.9E-06 |
| 3891 | PCSK1_RAT | Q9QXU9 | ProSAAS | 3888.04 | 3888.04 | 0 | 3890.32 |  | 1.5E-33 |
| 4800 | SCG2_RAT | P10362 | Secretogranin-2 | 4796.37 | 4796.37 | 0 | 4799.25 |  | 5.8E-72 |
| 4854 | VGF_RAT | P20156 | Neurosecretory protein VGF | 4850.41 | 4850.41 | 0 | 4853.24 |  | 1.4E-24 |
| 15825 | SODC_RAT | P07632 | Superoxide dismutase [Cu-Zn] | 15810.82 | 15810.75 | 4 | 15820.33 | N-term acetylation, disulfide bond | 3.2E-21 |

**Table S2.** Details of proteins identified merely by nanoLC-ESI-LTQ-Orbitrap Elite. The protein identities are sorted in the order of their monoisotopic masses. The resulting MS/MS spectra and fragment assignment can be found in the ppt. file and excel file included as **Supplementary Data**.

| **Entry** | **Accession** | **Protein Identity** | **Calc'd Mass (Da)** | | **Exp'd**  **Mass (Da)** | | **∆ Mass (ppm)** | | | **Average**  **Mass (Da)** | | **PTM** | **E value** | |  |
| --- | --- | --- | --- | --- | --- | --- | --- | --- | --- | --- | --- | --- | --- | --- | --- |
| CMGA_RAT | P10354 | Chromagranin-A | | 3174.66 | | 3174.66 | | 0 | 3176.5 | | C-term amidation | | | 6.8E-34 | |
| CCKN_RAT | P01355 | Cholecysokinin | | 3392.80 | | 3392.81 | | -3 | 3394.87 | |  | | | 2.5E-13 | |
| F1LP33_RAT | F1LP33 | Oxysterol-binding protein | | 3679.89 | | 3679.87 | | 5 | 3682.12 | |  | | | 1.2E-06 | |
| KCRU_RAT | P25809 | Creatine kinase U-type | | 3811.23 | | 3811.23 | | 0 | 3813.51 | | N-term acetylation | | | 4.2E-57 | |
| MDHM_RAT | P04636 | Malate dehydrogenase | | 3832.09 | | 3832.09 | | 0 | 3834.55 | |  | | | 7.0E-50 | |
| D4AAI2_RAT | D4AAI2 | Histocompatibility (minor) HA-1, isoform CRA_b | | 3975.99 | | 3975.99 | | 0 | 3978.30 | |  | | | 8.2E-05 | |
| D3ZMX3_RAT | D3ZMX3 | Neutralized-like (drosophila), isoform CRA_b | | 4008.92 | | 4008.91 | | 2 | 4011.38 | |  | | | 3.3E-07 | |
| TMM35_RAT | Q6JAM9 | transmembrane protein 35 | | 4036.15 | | 4036.16 | | -2 | 4038.52 | |  | | | 1.2E-36 | |
| NPY_RAT | P07808 | Proneuropeptide Y | | 4285.07 | | 4285.08 | | -2 | 4287.69 | | Oxidation, C-term amidation | | | 6.2E-34 | |
| CART_RAT | P49192 | Cocaine- and amphetamine- regulated transcript protein | | 4384.02 | | 4384.03 | | -2 | 4387.13 | | 3*Disulfide bonds | | | 1.3E-11 | |
| TYB10_RAT | P63312 | Thymosin beta-10 | | 4531.34 | | 4531.35 | | -2 | 4534.07 | | N-term acetylation, C-term methylation | | | 1.7E-69 | |
| NCAM1_RAT | P13596 | Neural cell adhesion molecule 1 | | 4548.23 | | 4548.23 | | 0 | 4550.86 | |  | | | 7.5E-41 | |
| TYB4_RAT | P62329 | Thymosin beta-4 | | 4616.35 | | 4616.35 | | 0 | 4619.13 | | N-term acetylation | | | 5.0E-62 | |
| TYB10_RAT | P63312 | Thymosin beta-10 | | 4620.36 | | 4620.36 | | 0 | 4623.12 | |  | | | 9.4E-13 | |
| SCG2_RAT | P10362 | Secretogranin-2 | | 4683.28 | | 4683.29 | | -2 | 4683.29 | |  | | | 2.1E-70 | |
| TYB4_RAT | P62329 | Thymosin beta-4 | | 4744.4 | | 4744.41 | | -2 | 4747.26 | | N-term acetylation | | | 3.1E-68 | |
| TYB10_RAT | P63312 | Thymosin beta-10 | | 4747.42 | | 4747.42 | | 0 | 4750.26 | | N-term acetylation, C-term/R methylation | | | 6.9E-72 | |
| TYB10_RAT | P63312 | Thymosin beta-4 | | 4758.42 | | 4758.43 | | -2 | 4761.28 | | N-term acetylation, C-term methylation | | | 1.2E-81 | |
| D3ZWN0_RAT | D3ZWN0 | Protein Plekha6 | | 4774.41 | | 4774.42 | | -2 | 4777.19 | |  | | | 1.6E-14 | |
| TYB10_RAT | P63312 | Thymosin beta-4 | | 4976.47 | | 4976.48 | | -2 | 4979.45 | | N-term acetylation, oxidation | | | 7.7E-86 | |
| F1LUV9_RAT | F1LUV9 | Uncharacterized | | 5241.65 | | 5241.65 | | 0 | 5244.69 | |  | | | 3.0E-39 | |
| NEUM_RAT | P07936 | Neuromodulin | | 5261.39 | | 5261.40 | | -2 | 5264.38 | |  | | | 5.7E-86 | |
| CCKN_RAT | P01355 | CCK | | 5434.99 | | 5435.00 | | -2 | 5438.25 | |  | | | 7.7E-44 | |
| D4AA63_RAT | D4AA63 | Ubiquitin | | 5895.01 | | 5895.01 | | 0 | 5898.55 | |  | | | 1.0E-55 | |
| ATP5J_RAT | P21571 | ATP synthase-coupling factor 6 | | 6024.88 | | 6024.89 | | -2 | 6028.65 | |  | | | 4.0E-15 | |
| PCP4_RAT | P63055 | PEP-19 | | 6730.24 | | 6730.25 | | -1 | 6734.19 | | N-term acetylation, oxidation | | | 4.4E-32 | |
| NEUM_RAT | P07936 | Neuromodulin | | 7963.66 | | 7963.70 | | -5 | 7968.25 | |  | | | 1.7E-25 | |
| F1LNN9_RAT | F1LNN9 | Uncharacterized protein | | 8155.96 | | 8155.94 | | 2 | 8160.64 | |  | | | 3.9E-28 | |
| RS27A_RAT | P62982 | Ubiquintin-40S ribosomal protein S27a | | 8445.57 | | 8445.57 | | 0 | 8450.65 | |  | | | 2.1E-43 | |
| RS27A_RAT | P62982 | Ubiquintin-40S ribosomal protein S27a | | 8459.59 | | 8459.59 | | 0 | 8464.68 | | C-terminal/R methylation | | | 2.7E-32 | |
| NEDD8_RAT | Q71UE8 | NEDD8 | | 8554.67 | | 8554.67 | | 0 |  | |  | | | 2.1E-34 | |
| RS27A_RAT | P62982 | Ubiquintin-40S ribosomal protein S27a | | 8575.60 | | 8575.61 | | -1 | 8580.75 | | Oxidation | | | 2.0E-48 | |
| UFM1_RAT | Q5BJP3 | Ubiquitin-fold modifier 1 | | 8832.80 | | 8832.80 | | 0 | 8838.1 | | N-term acetylation | | | 1.1E-41 | |
| ATP5J_RAT | P21571 | ATP synthase-coupling factor 6 | | 8937.52 | | 8937.55 | | -3 | 8943.06 | | Oxidation | | | 1.5E-37 | |
| ACBP_RAT | P11030 | Acyl-CoA-binding protein | | 9762.01 | | 9762.02 | | -1 | 9767.99 | | N-term acetylation | | | 9.4E-13 | |
| DLRB1_RAT | P62628 | Dynein light chain roadblock-type 1 | | 10893.73 | | 10893.73 | | 0 | 10893.73 | | N-term acetylation | | | 2.0E-53 | |
