# Supplementary material for: Top-Down Proteomics with Mass Spectrometry Imaging: A Pilot Study towards Discovery of Biomarkers for Neurodevelopmental Disorders
Source: PLoS One. 2014 Apr 7;9(4):e92831. doi: 10.1371/journal.pone.0092831 (PMC3978070; doi:10.1371/journal.pone.0092831)

Supplemental information of the proteins identified via MALDI-TOF/TOF and nanoLC-ESI-LTQ-Orbitrap ELITE as included in **Table 1 (Table S1)** and **Table 2**. The protein information and resulting MS/MS spectra are shown in the fashion as below:

Average mass as observed on MALDI-MS	Entry	Accession	Protein description	Calc'd mass (Da)	Exp'd mass (Da)	Mass difference (ppm)	PTM	E-value

Protein sequence, coverage and assigned PTMs

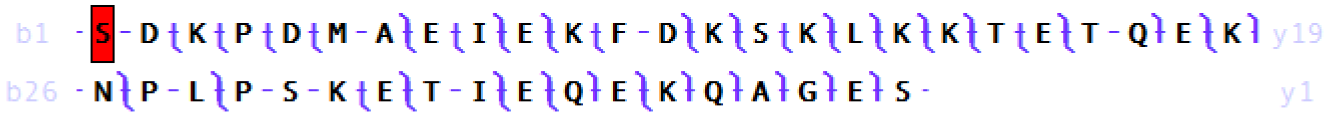

The MS/MS spectrum that corresponds to the sequence assignment is shown. The resulting fragment ions are listed in the excel sheets.

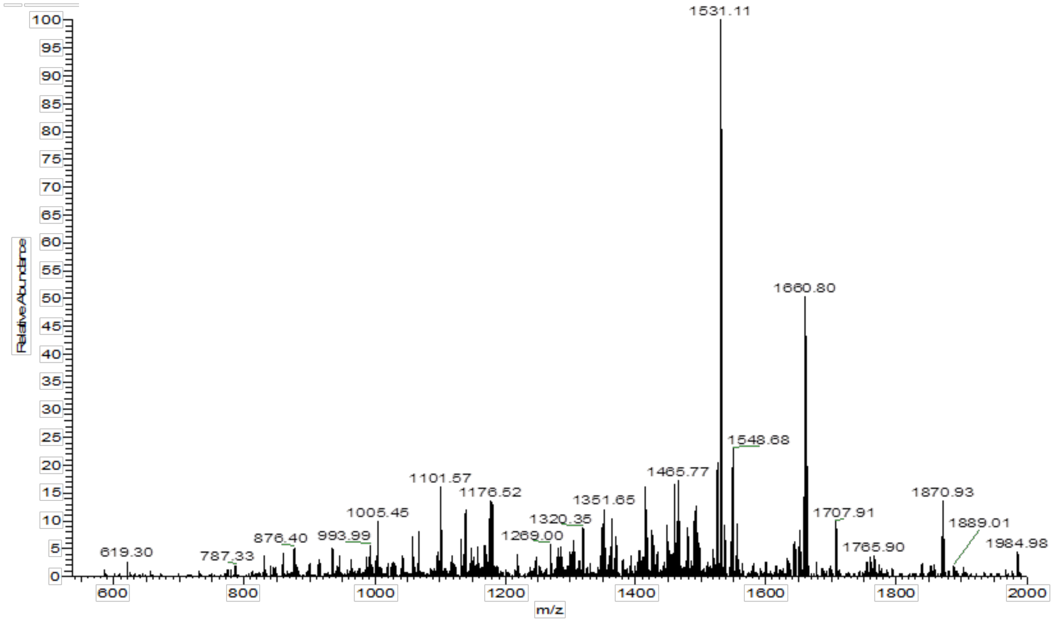

Table 1

4964	TYB10_RAT	P63312	Thymosin β-4	4960.48	4960.49	-2	N-term Acetylation	5.0E-77

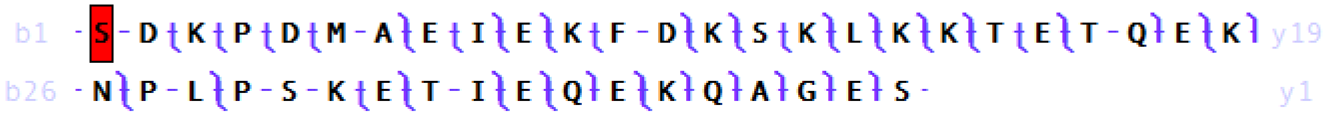

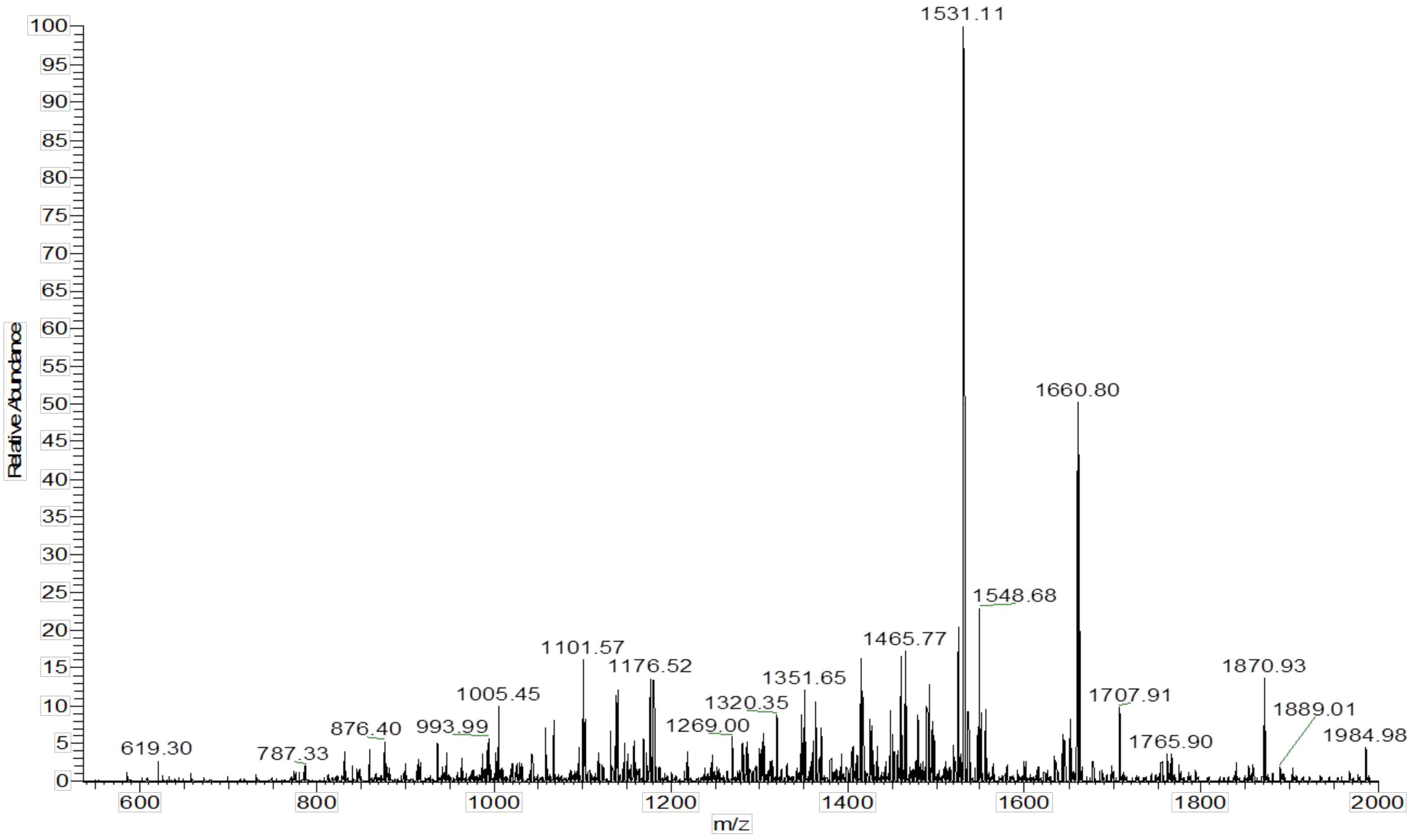

Table 1

9939	ACBP_RAT	P11030	Acyl-CoA-binding protein	9932.12	9932.12	0	N-term acetylation	1.2E-44

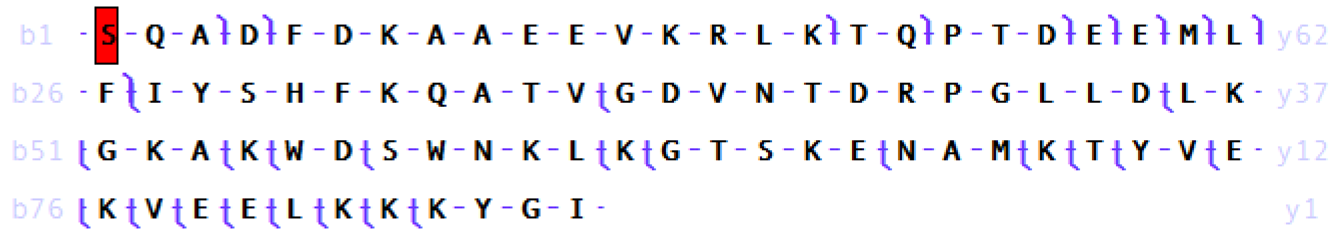

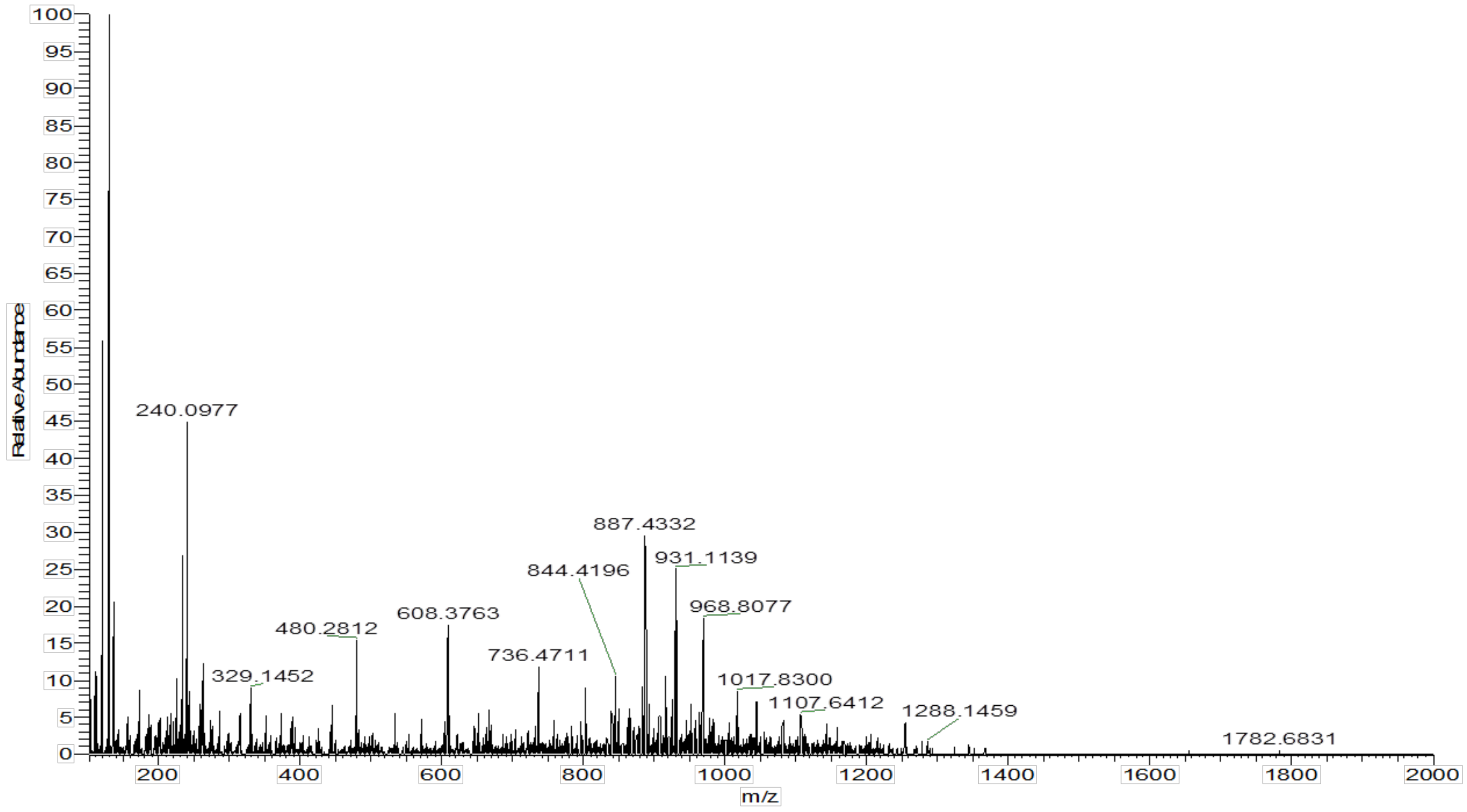

Table 1

9979	D3ZD09_ RAT	D3ZD09	cytochrome c oxidase polypeptide VI b	9971.80	9971.82	-2	N-term acetylation, 2 disulfide bonds	1.8E-41

b1 - A - E - D I - K - T K I K - N - Y K T A P - F - D S - R - F - P - N Q - N - Q - y61
b26 - T - K - N - C - W - Q - N - Y - L - D - F - H - R - C - E - K - A - M - T - A - K - G - G - D - V - y36
b51 - S - V - C - E - W - Y - R - R - V - Y - K - S - L - C - P - V - S - W - V S A W D D R - y11
b76 - I - A - E G - T F P G K - I - y1

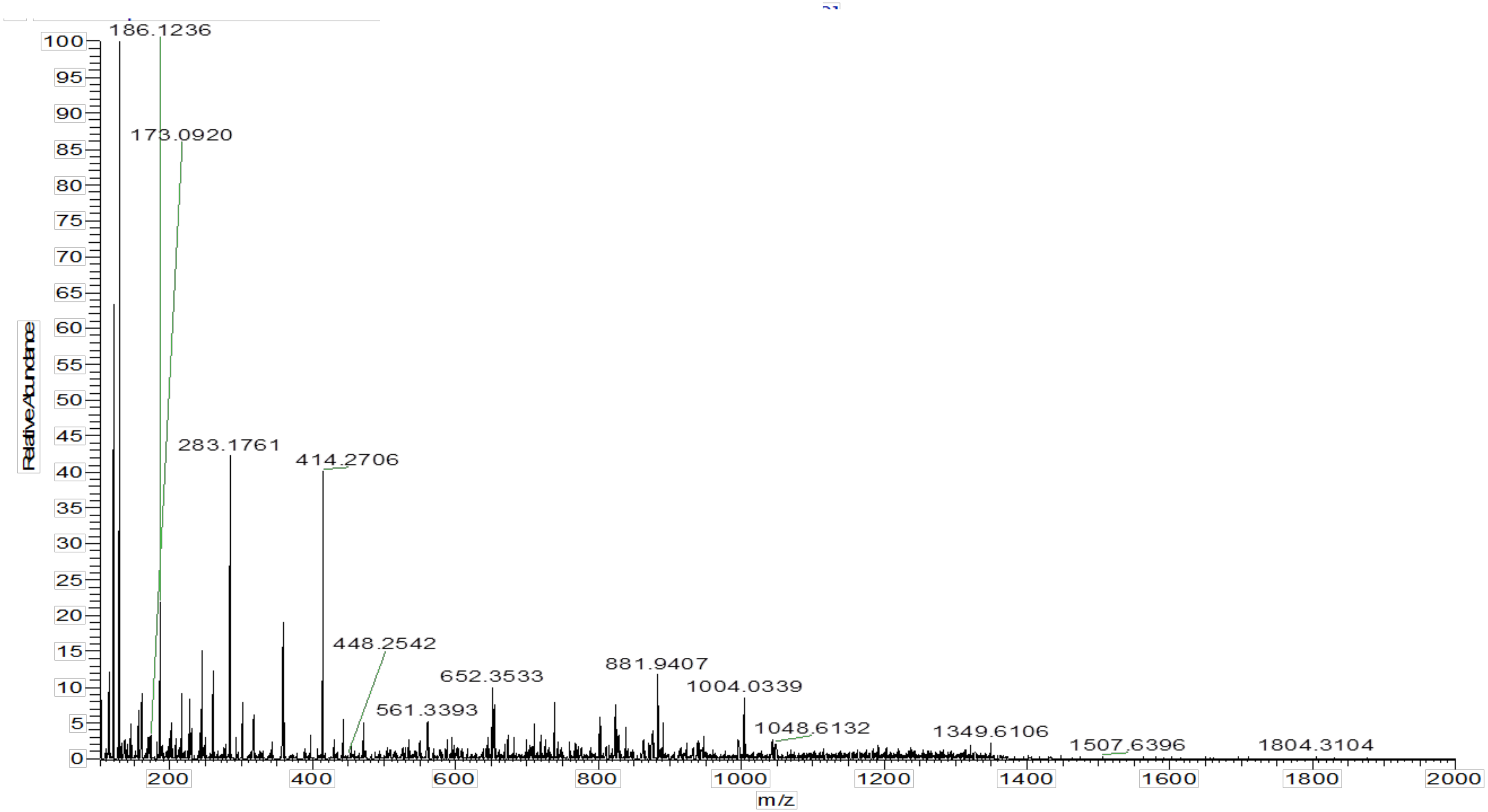

Table 1

5486	B2RYT3_RAT	B2RYT3	Cox7c protein	5481.87	5481.87	0		1.5E-61

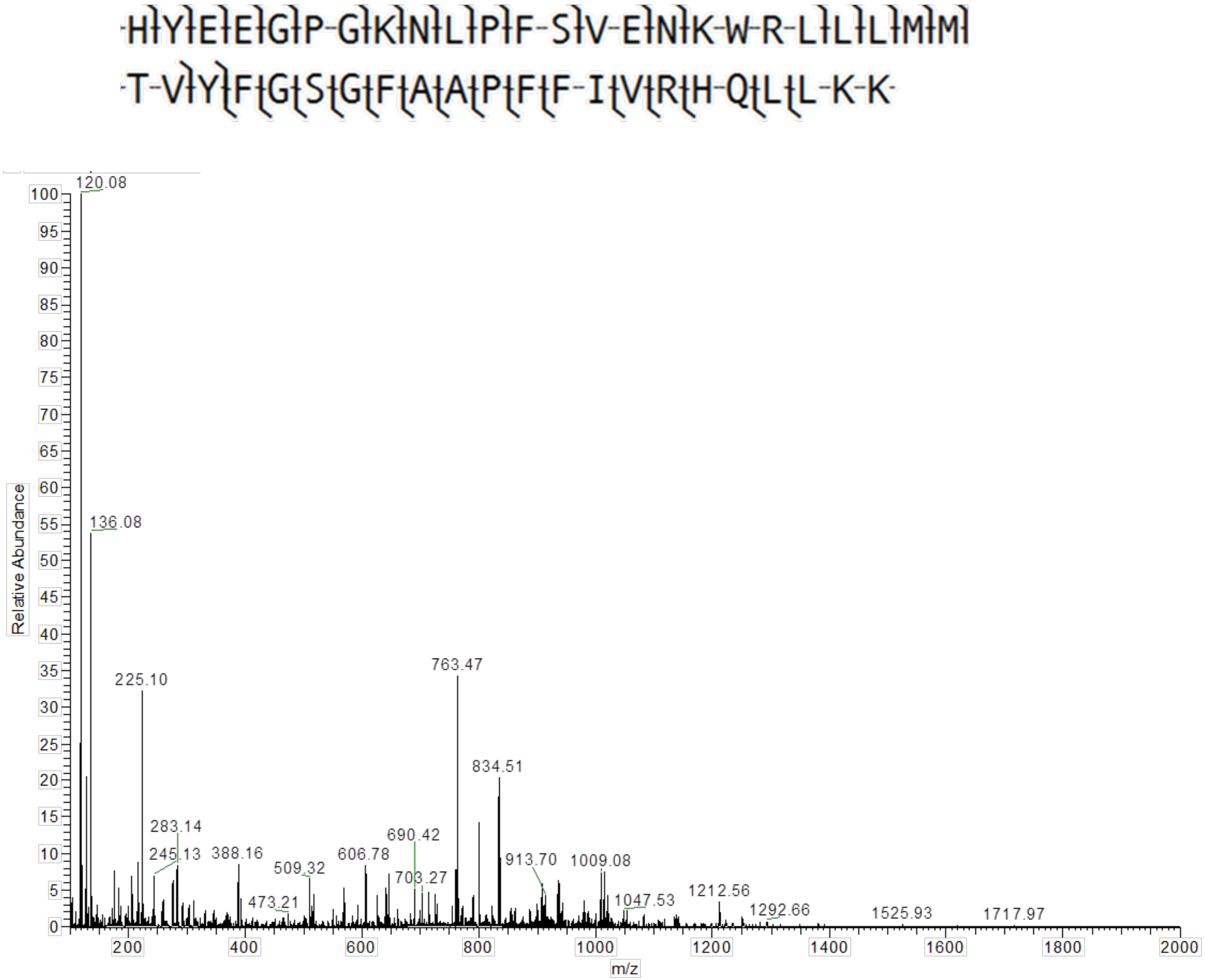

Table 1

10283	TIM9_RAT	Q9WV97	Mitochondria import inner membrane translocase	10276.08	10276.09	-1	N-term acetylation, 2 disulfide bonds	8.0E-24

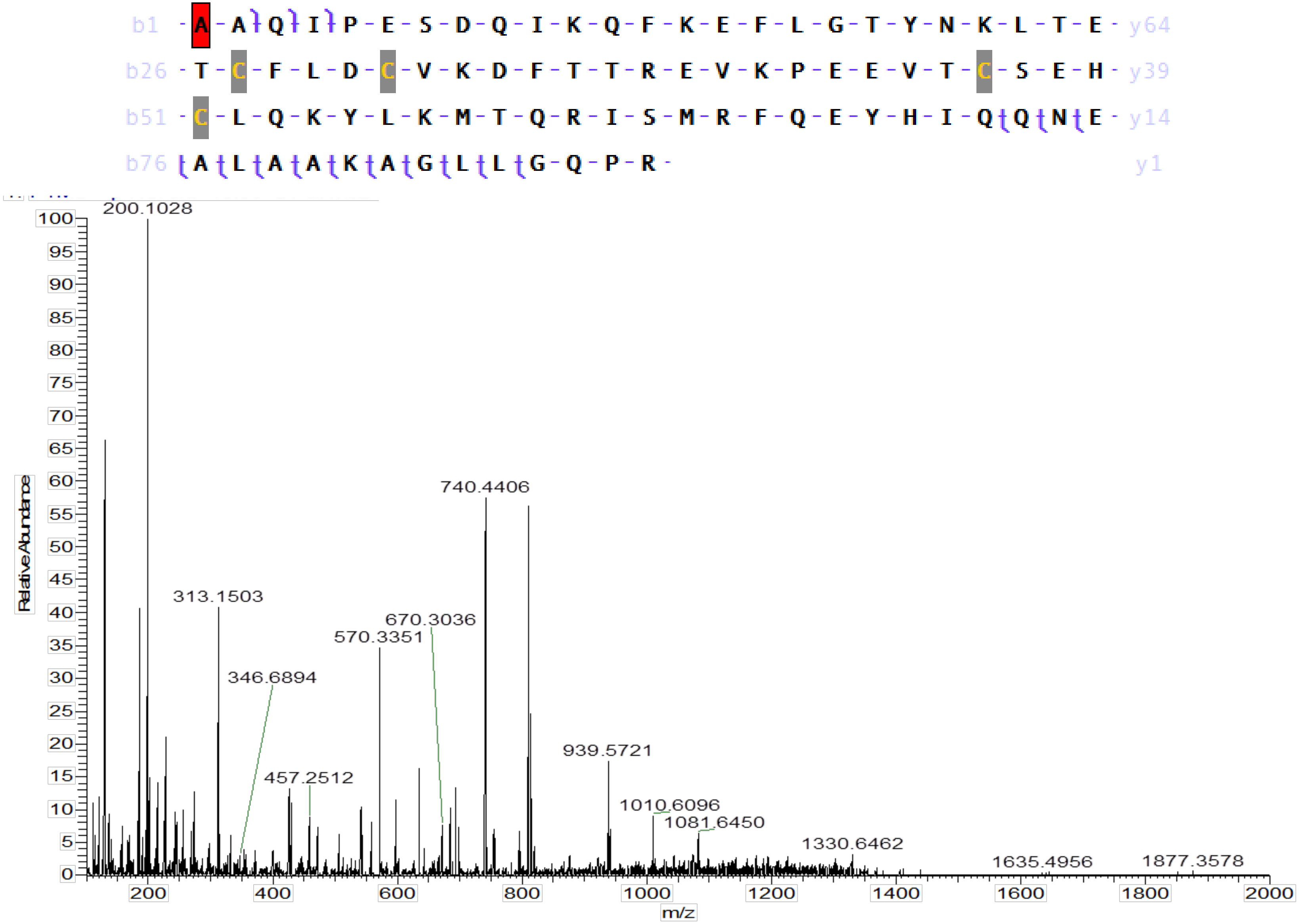

Table 1

9193	TIM8B_RAT	P62078	Adenylate cyclase type 10	9187.44	9187.45	-1	N-term acetylation, 2 disulfide bonds	2.3E-25

b1 - A - E - L - G - E - A - D - E - A - E - L - Q - R - L - V - A - A - E - Q - Q - K - A - Q - F - T - y58
b26 - A - Q - V - H - H - F - M - E - L - C - W - D - K - C - V - E - K - P - G - S - R - L - D - S - R - y33
b51 - T - E - N - C - L - S - S - C - V - D - R - F - I - D - T - T - L - A - I - T - G - R - F - A - Q - y8
b76 - I - V - O - K - G - G - O - y1

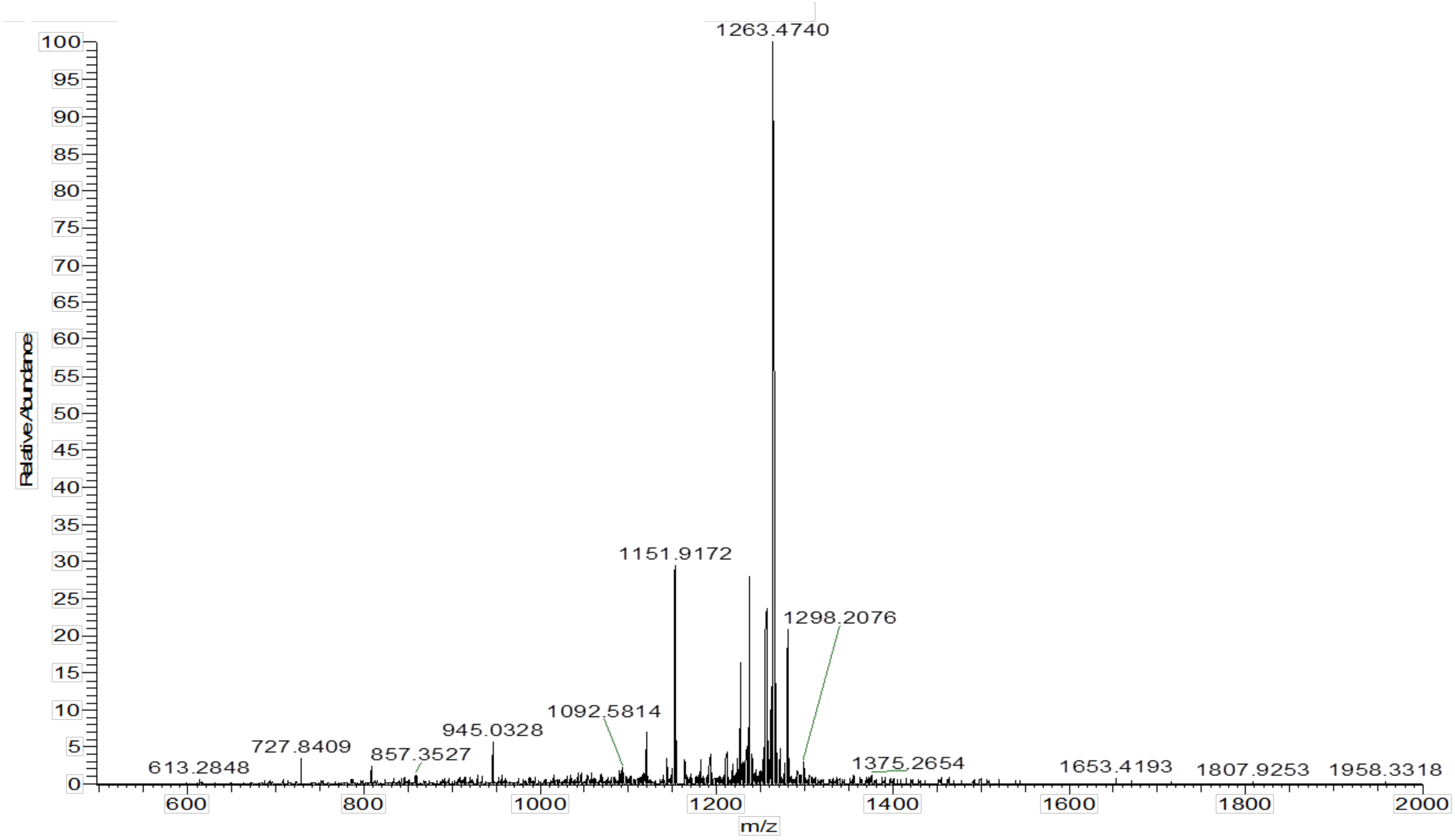

Table 1

8040	UCRI_RAT	P20788	Cytochrome b-c1 complex subunit	8035.38	8035.38	0	N-term acetylation	4.4E-39

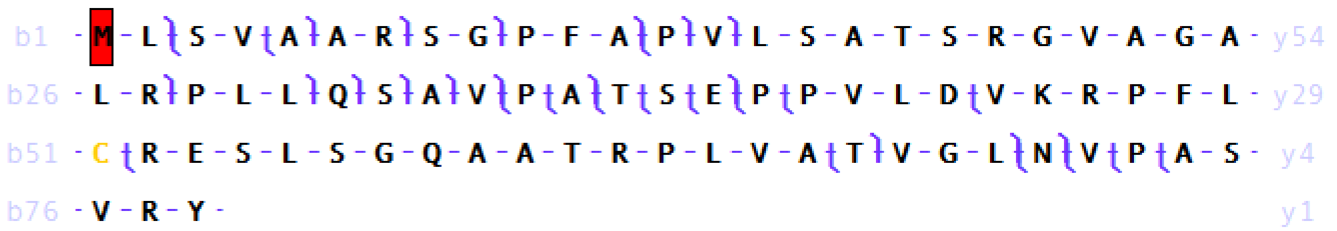

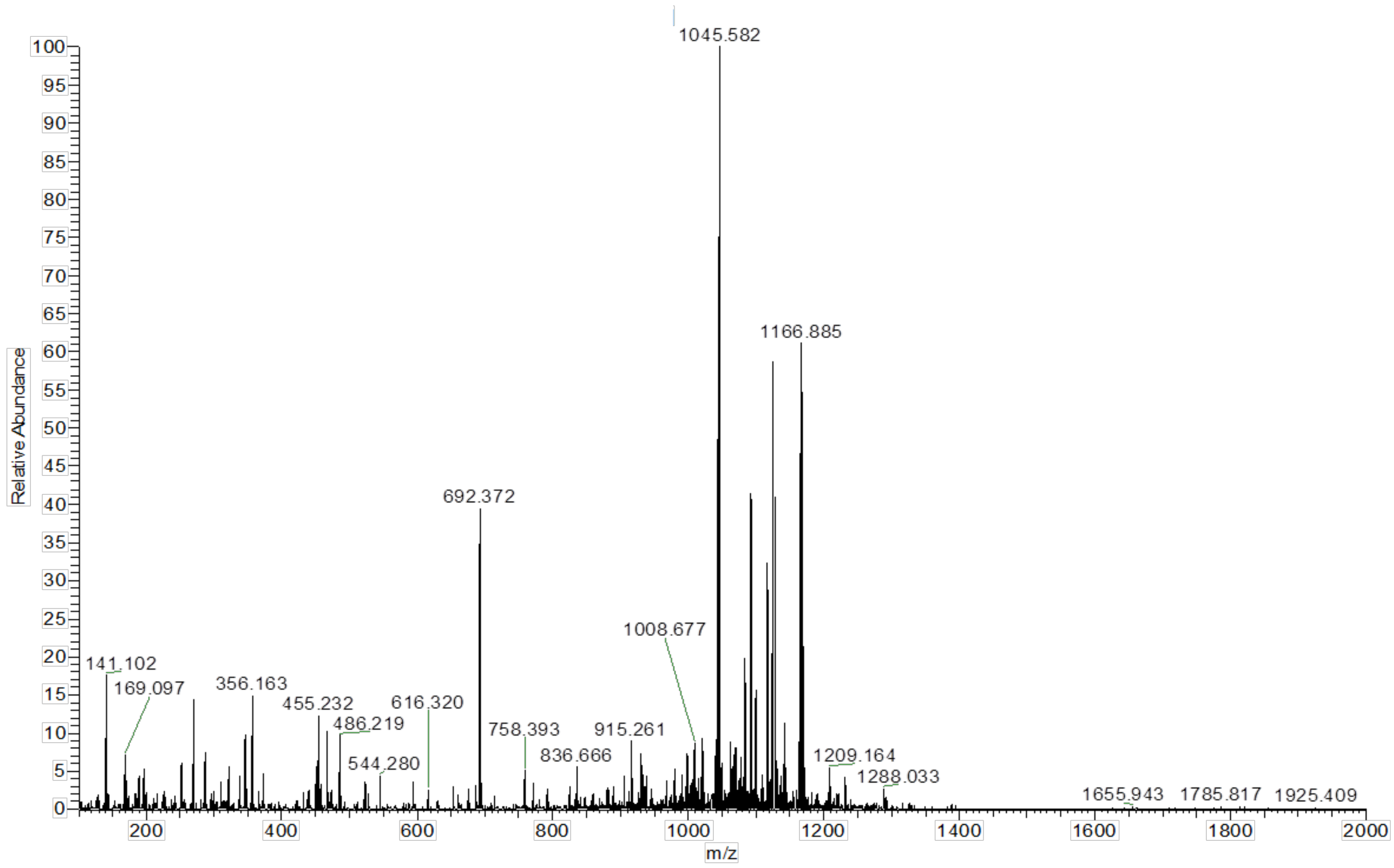

Table 1

4936	TYB10_RAT	P63312	Thymosin beta-10	4933.51	4933.52	-2	N-term acetylation	7.5E-87

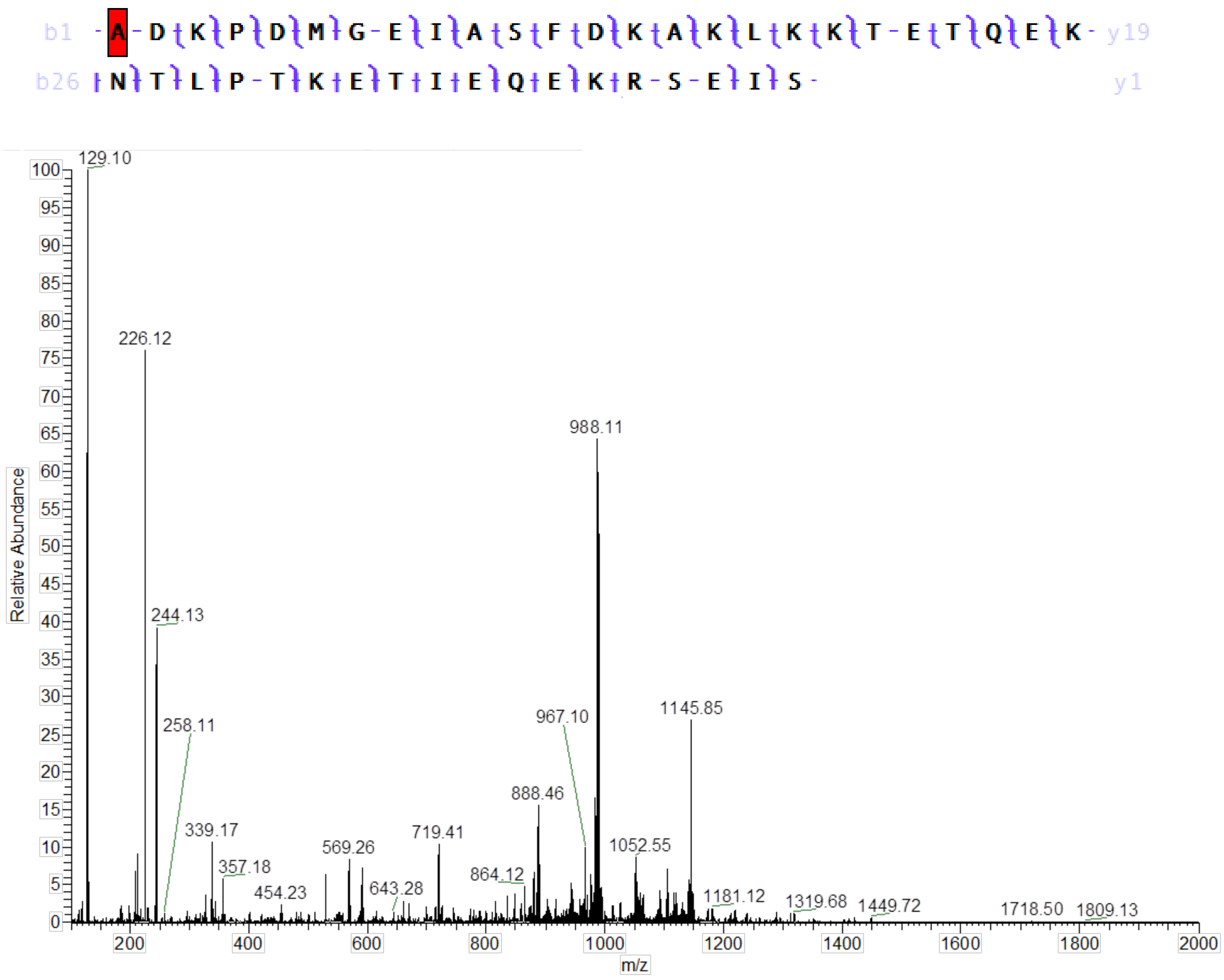

Table 1

3430	ATP5J_RAT	P21571	ATP synthase-coupling factor 6	3426.89	3426.90	-3		4.3E-13

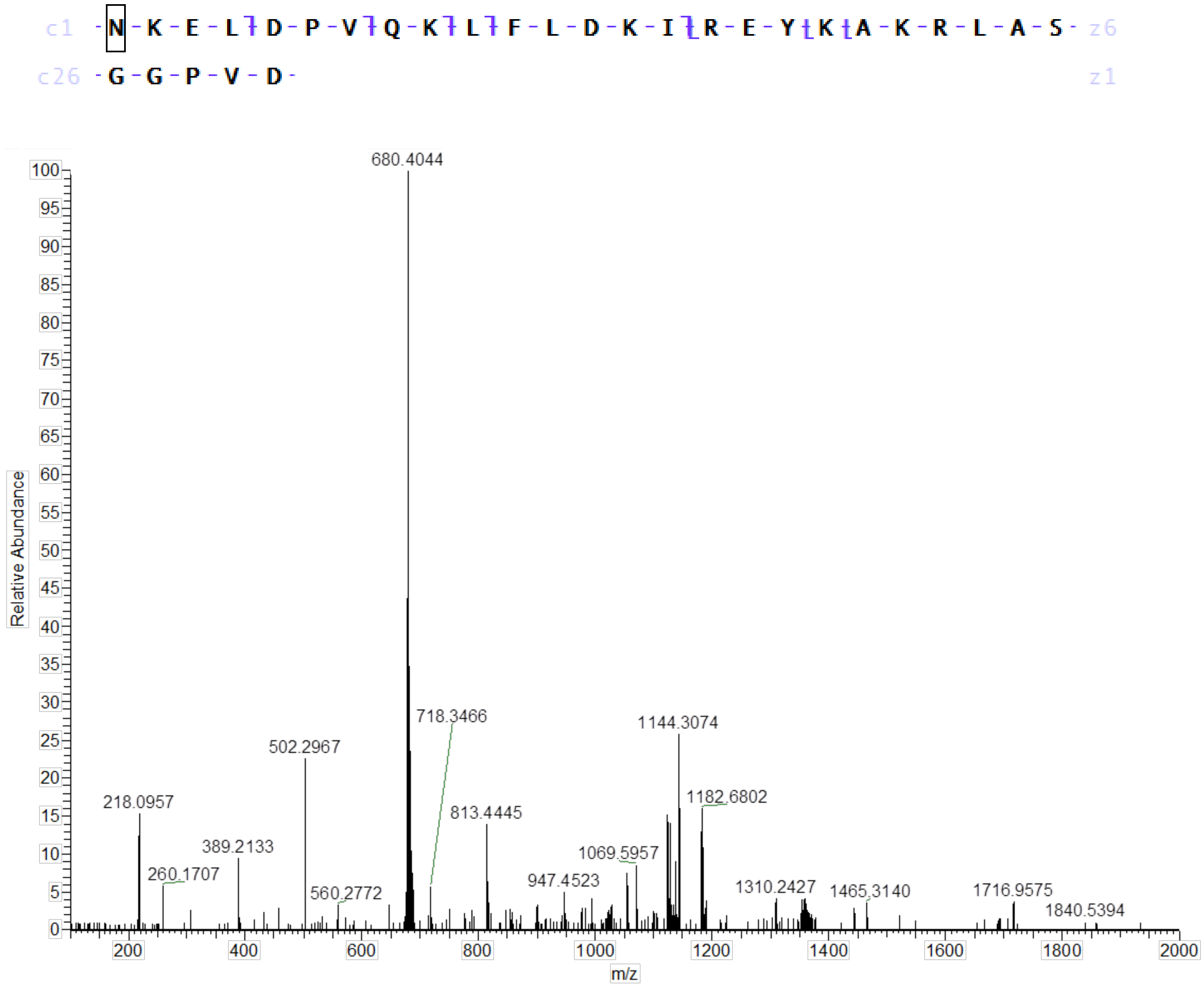

Table 1

16792	CALM_RAT	P62161	Calmodulin	16779.82	16779.81	1	N-term acetylation, unfixed acetylation	1.3E-10

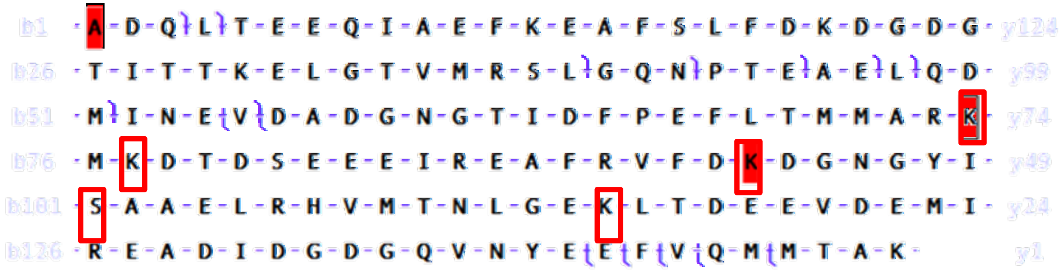

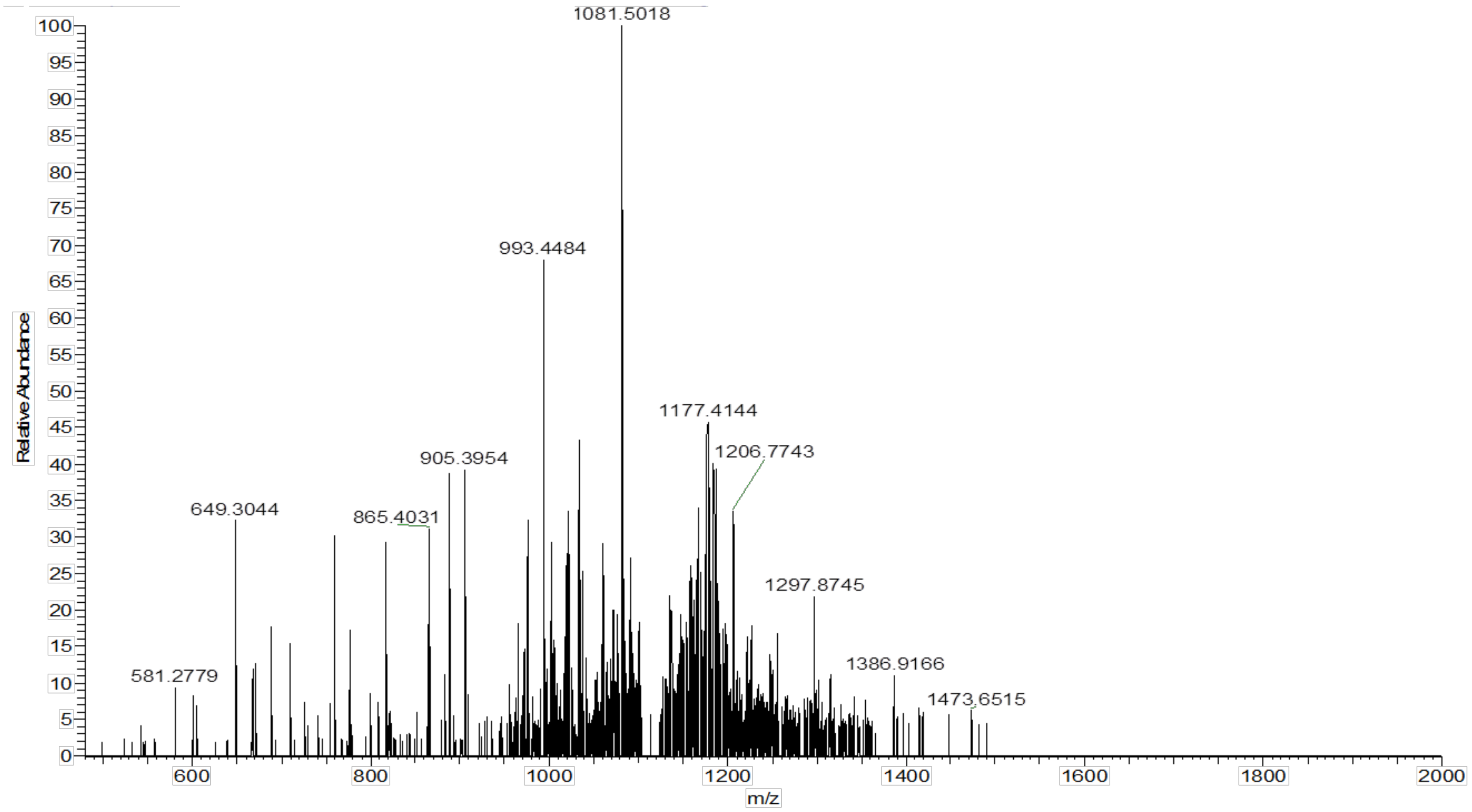

Table 1

3717	Q5U2U9_RAT	Q5U2U9	CCR4-NOT transcription complex, subunit 8	3713.87	3713.88	-3		7.9E-06

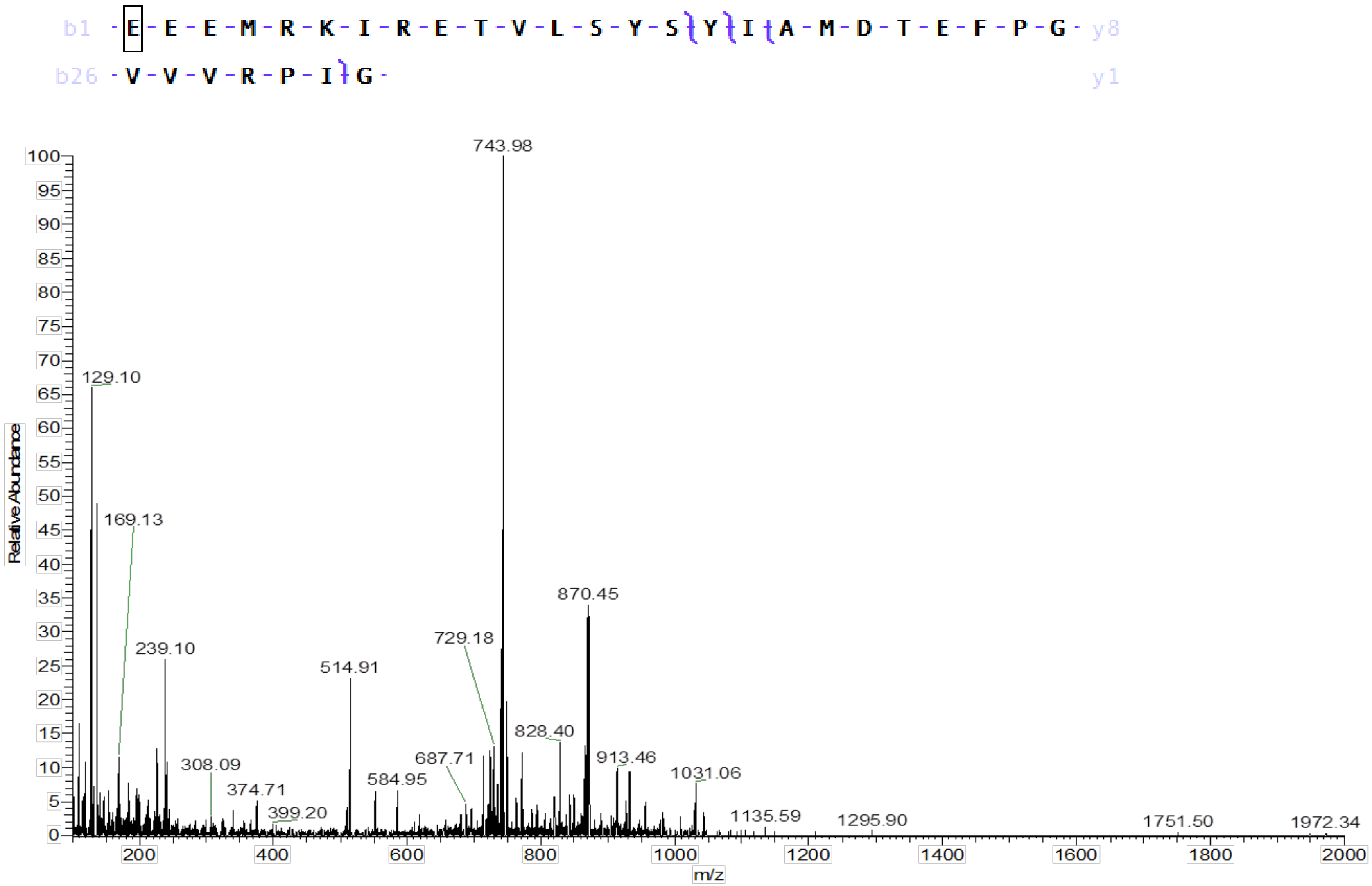

Table 1

3891	PCSK1_RAT	Q9QXU9	ProSAAS	3888.04	3888.04	0		1.5E-33

b1 - A A D E T P D V D P - E L - L - R Y - L - L - G - R I - L - T G - S - S - y12

b26 - E P - E A A P - A - P - R - R L - y1

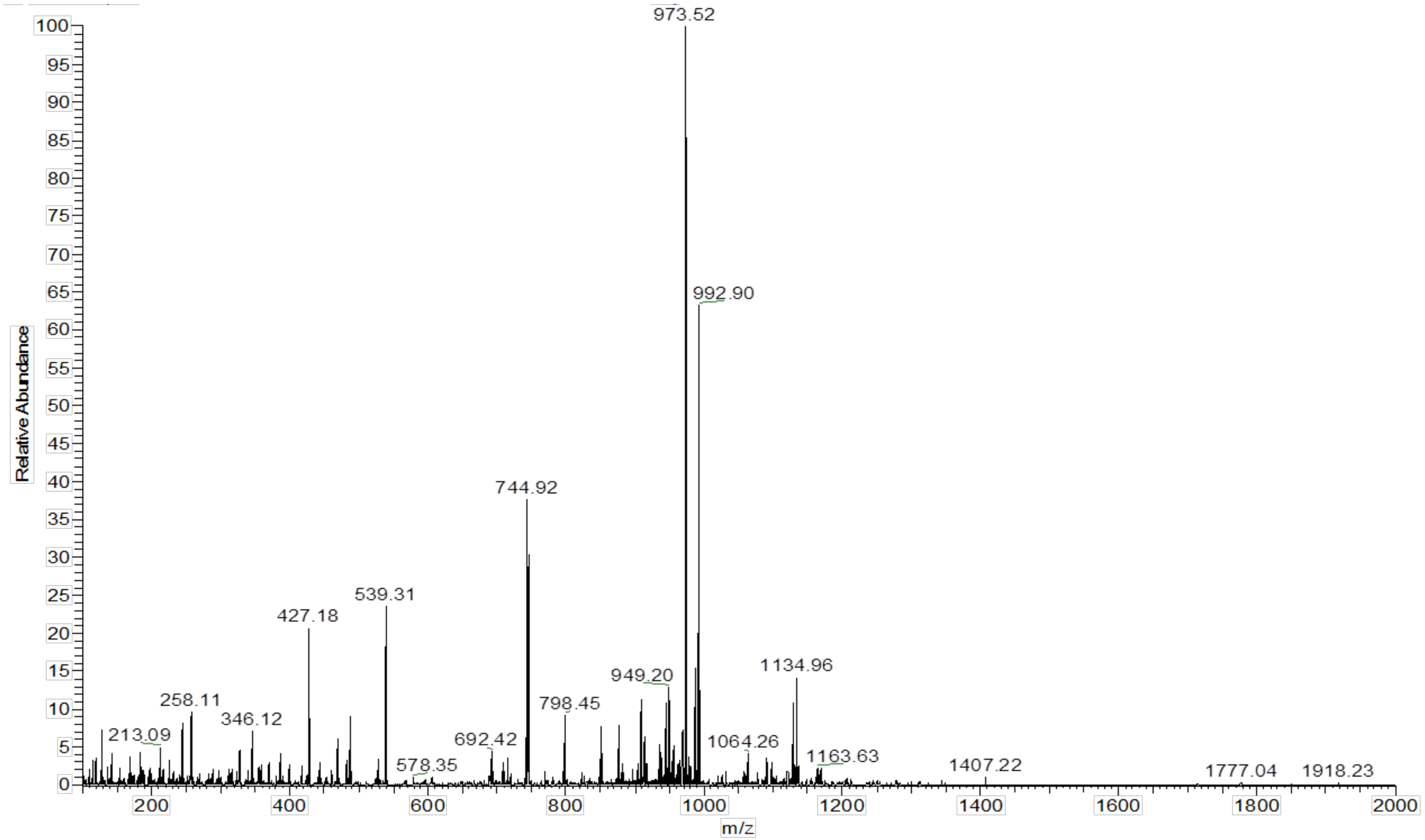

Table 1

4800	SCG2_RAT	P10362	Secretogranin-2	4796.37	4796.37	0		5.8E-72

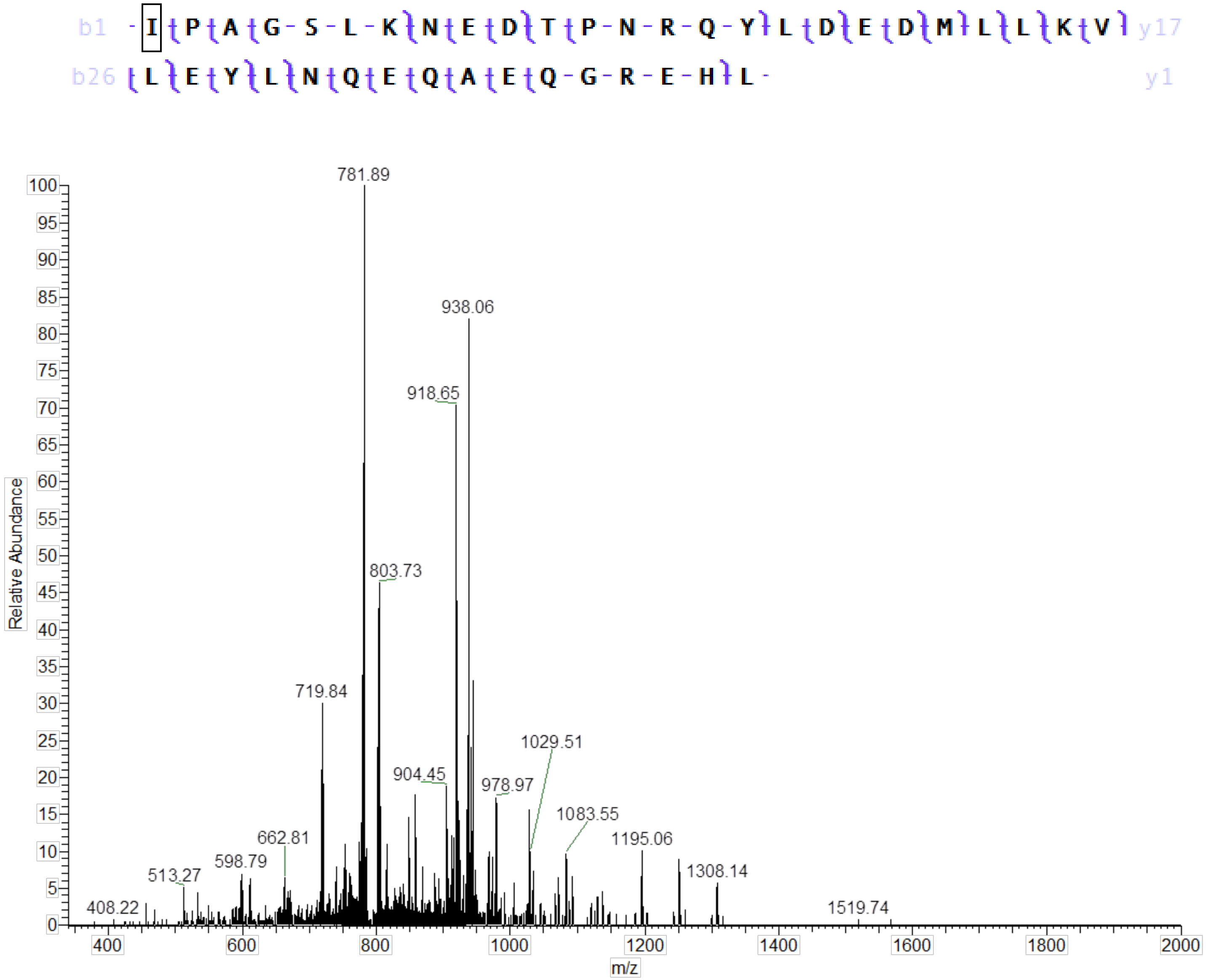

Table 1

4854	VGF_RAT	P20156	Neurosecretory protein VGF	4850.41	4850.41	0		1.4E-24

b1 - N - S - E - P - Q - D - Q - G - E - L - F - Q - G - V - D - P - R - A - L - A - A - V - L - L - Q - y23

b26 - A - L - D - R - P - A - S - P - P - A - V - P - A - G - S - Q - Q - G - T - P - E E - y1

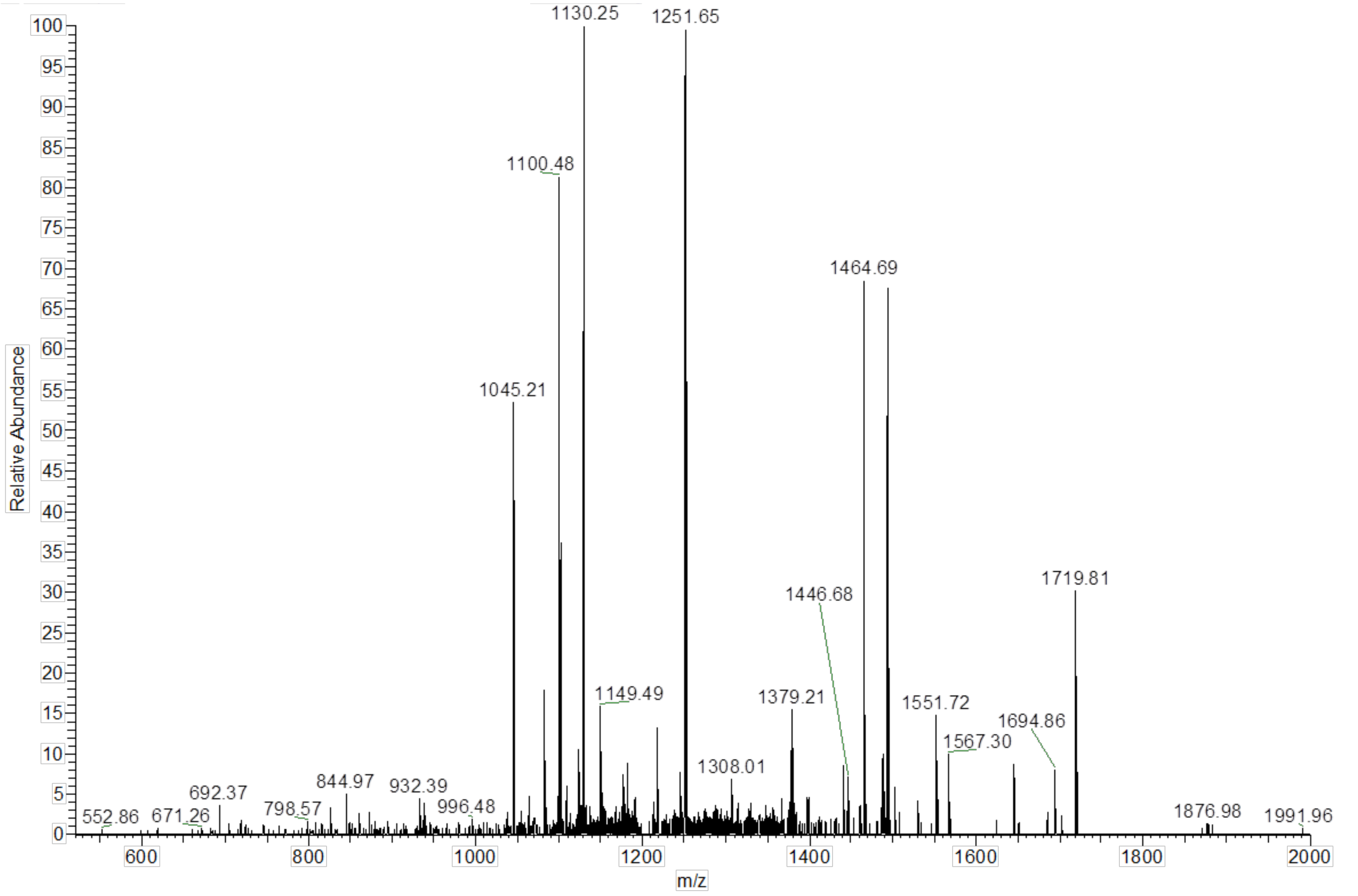

Table 1

15825	SODC_RAT	P07632	Superoxide dismutase [Cu-Zn]	15810.82	15810.75	4	N-term acetylation, disulfide bond	3.2E-21

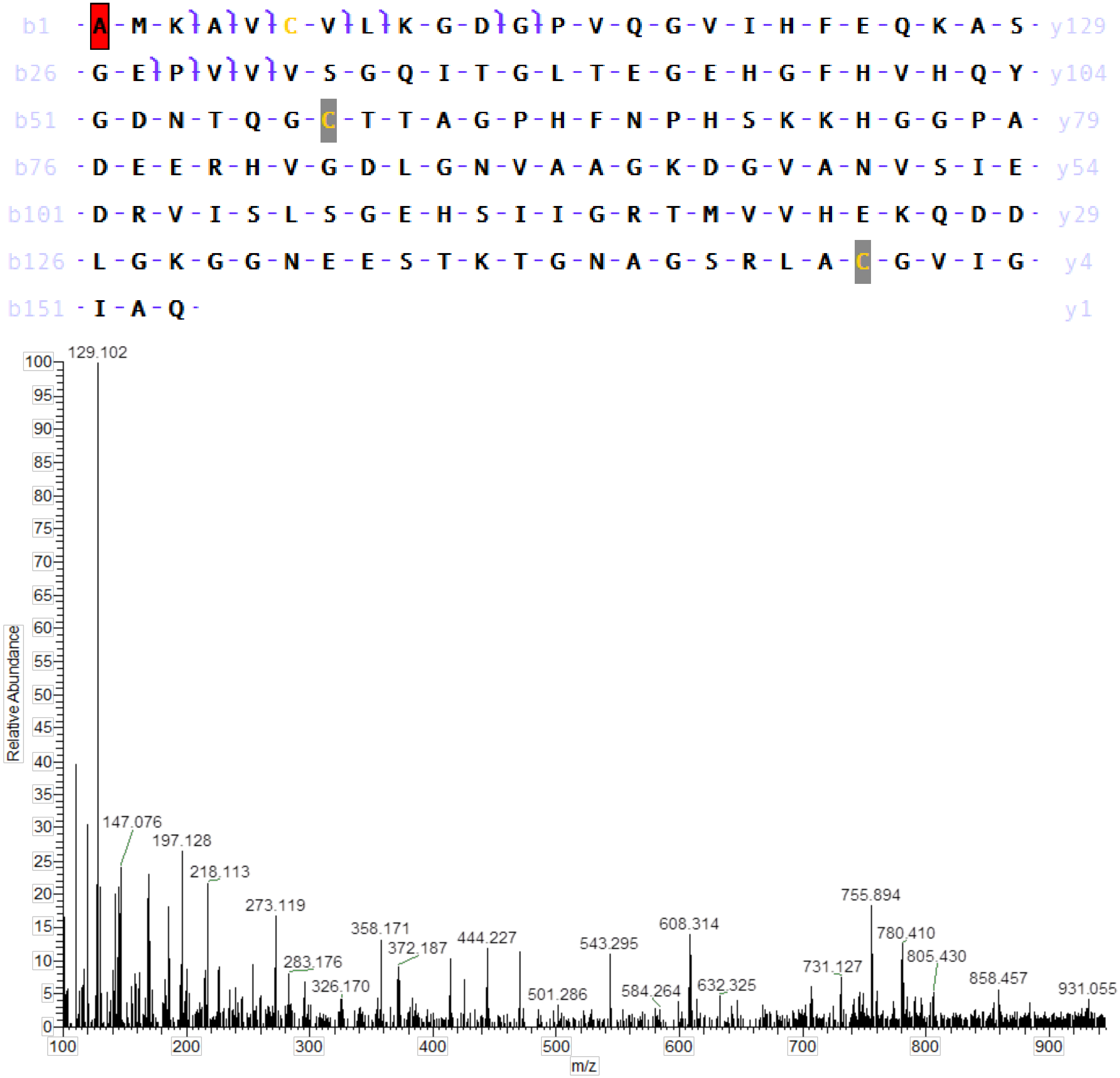

Table 2

3327	VIP_RAT	P01283	VIP peptides	3323.75	3323.76	-3	C-term amidation	8.3E-14

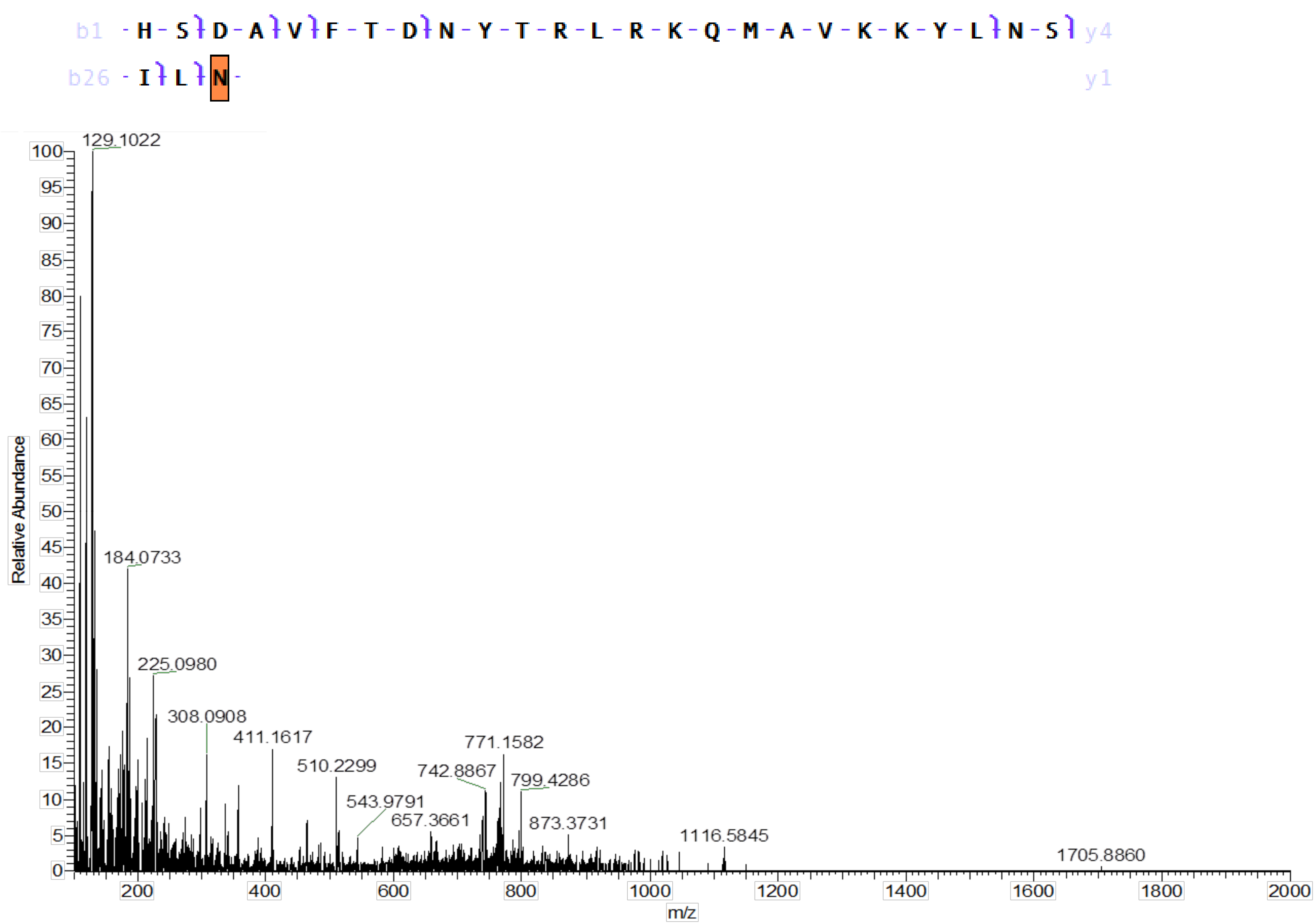

Table 2

3437	7B2_RAT	P27682	Neuroendocrine protein 7B 2	3433.78	3433.79	-3		7.2E-39

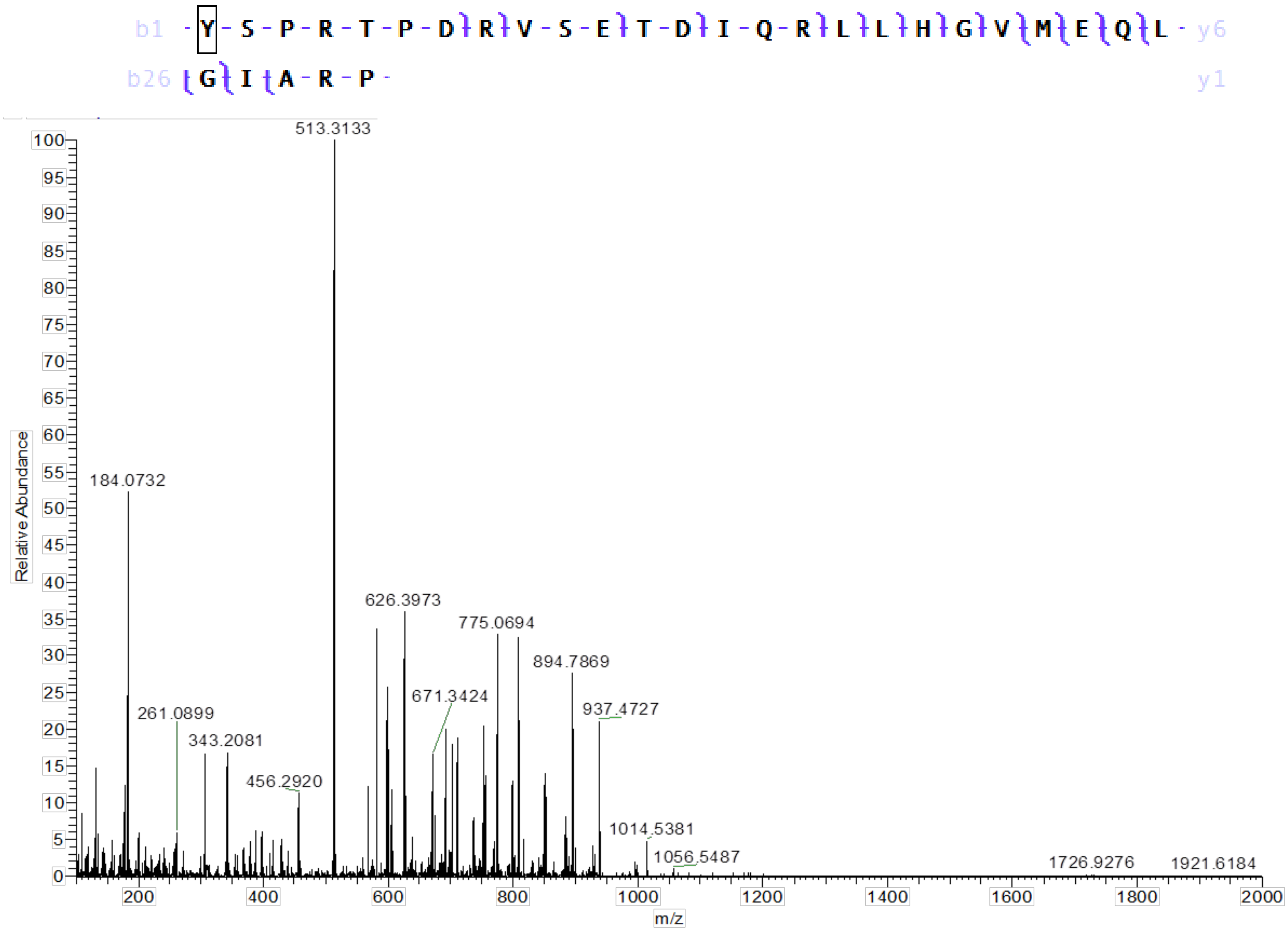

Table 2

3464	NPY_RAT	P07808	Pro-Neuropeptide Y	3460.66	3460.66	0		1.6E-18

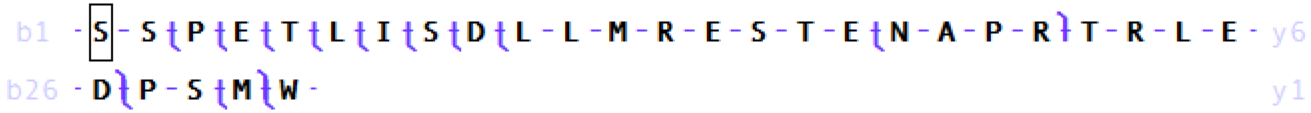

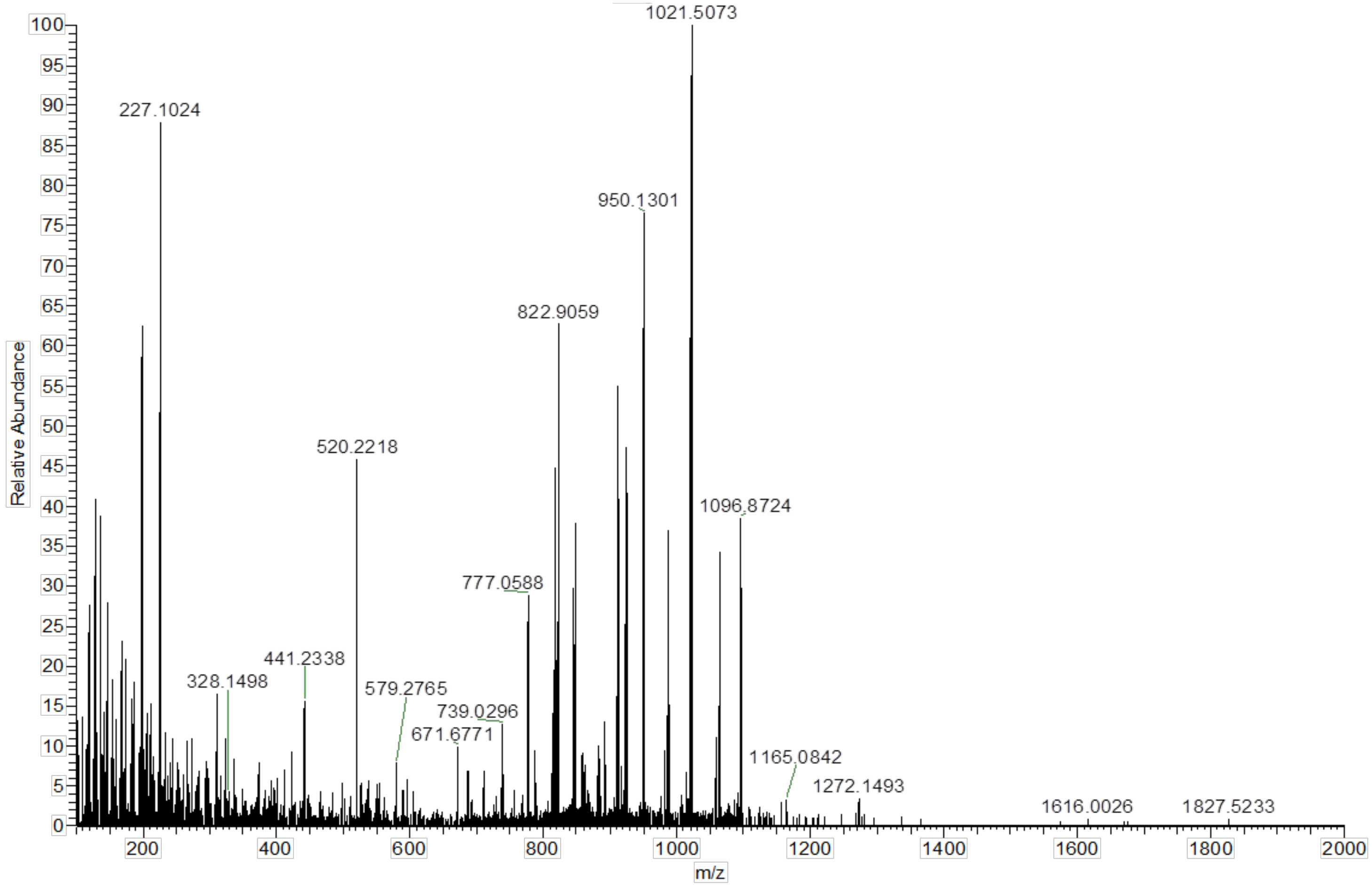

Table 2

3593	7B2_RAT	P27682	Neuroendocrine protein 7B 2	3589.89	3589.89	0		2.5E-60

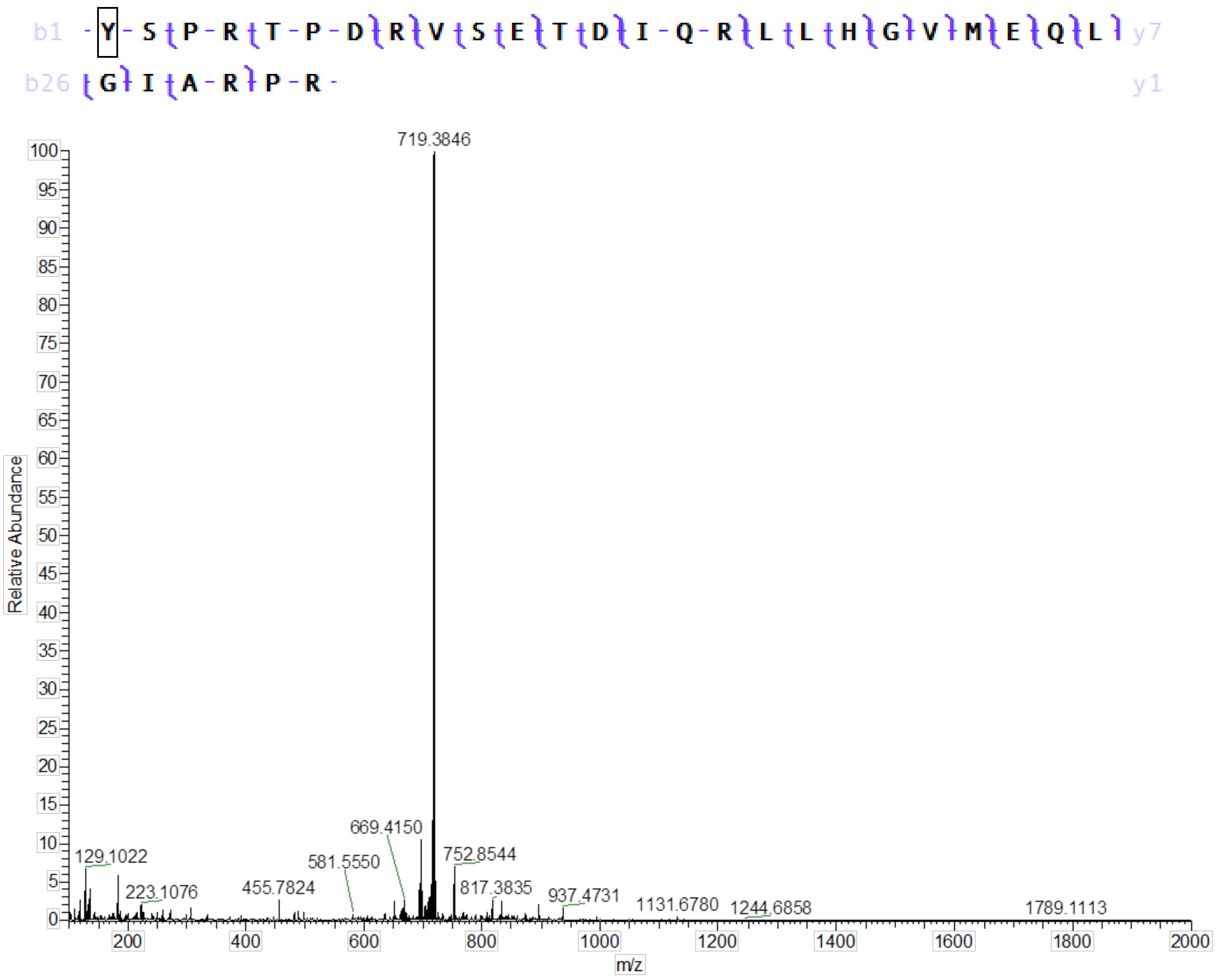

Table 2

3653	SCG2_RAT	P10362	Secretogranin 2	3649.80	3649.80	0		3.8E-67

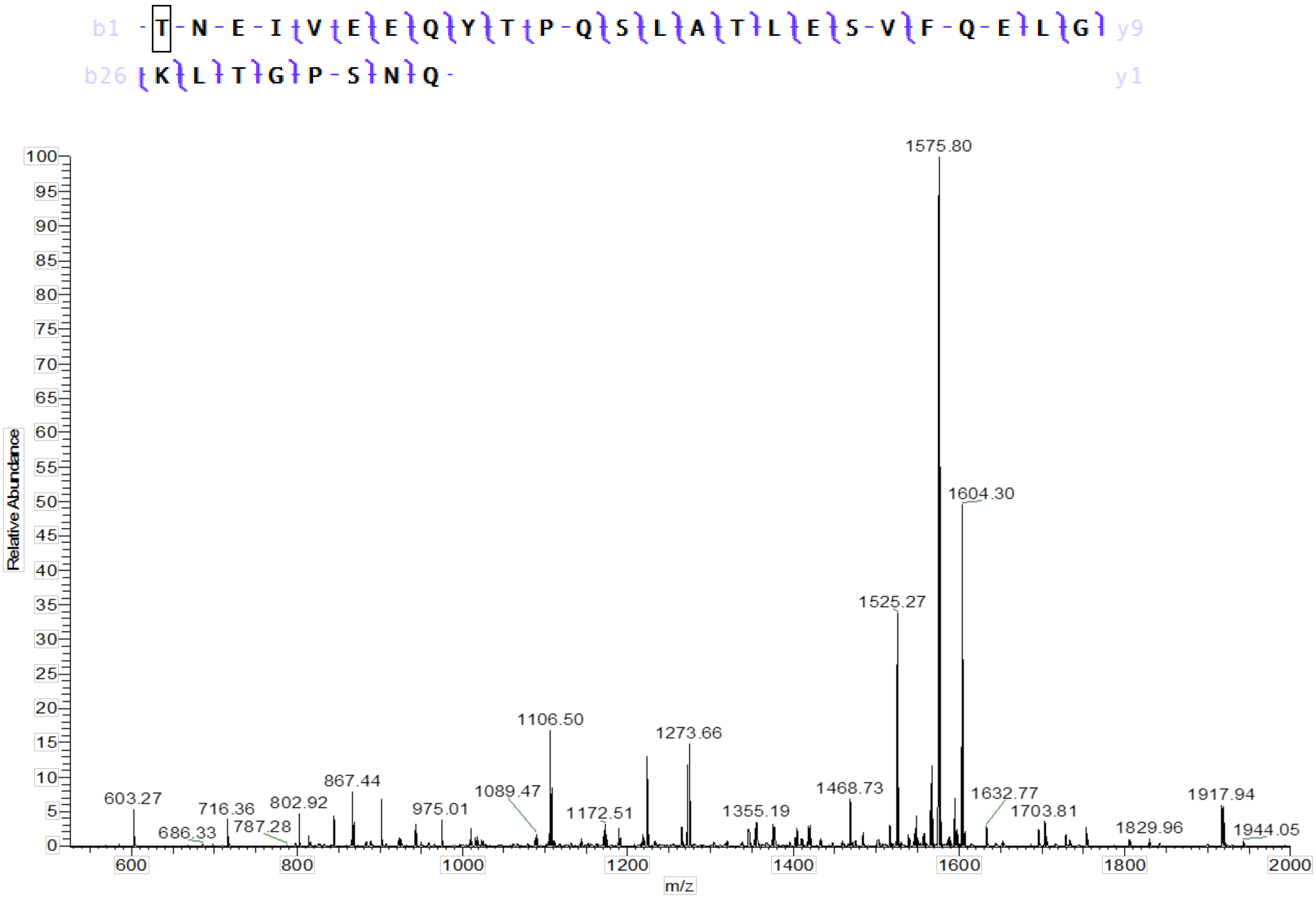

Table 2

3676	VGF_RAT	P20156	Neurosecretory protein VGF	3672.77	3672.78	-3		8.0E-08

b1 - A - Q - E - E - A - D - A - E - E - R - R - L - Q - E - Q - E - E - L - E - N - Y - I - E - H - V - y6

b26 - L - L - H - R - P - y1

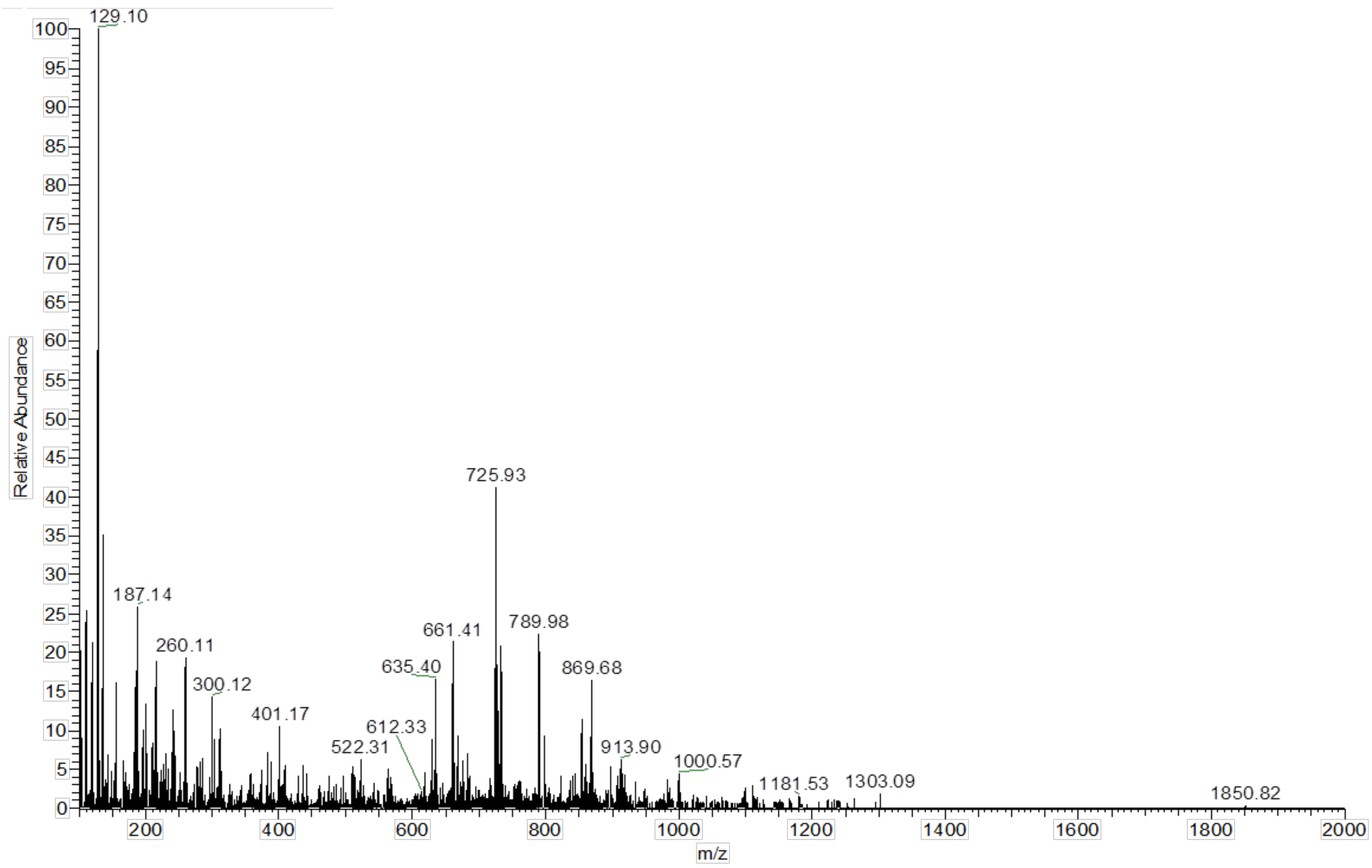

Table 2

3871	VGF_RAT	P20156	Neurosecretory protein VGF	3867.86	3867.87	-3		2.9E-37

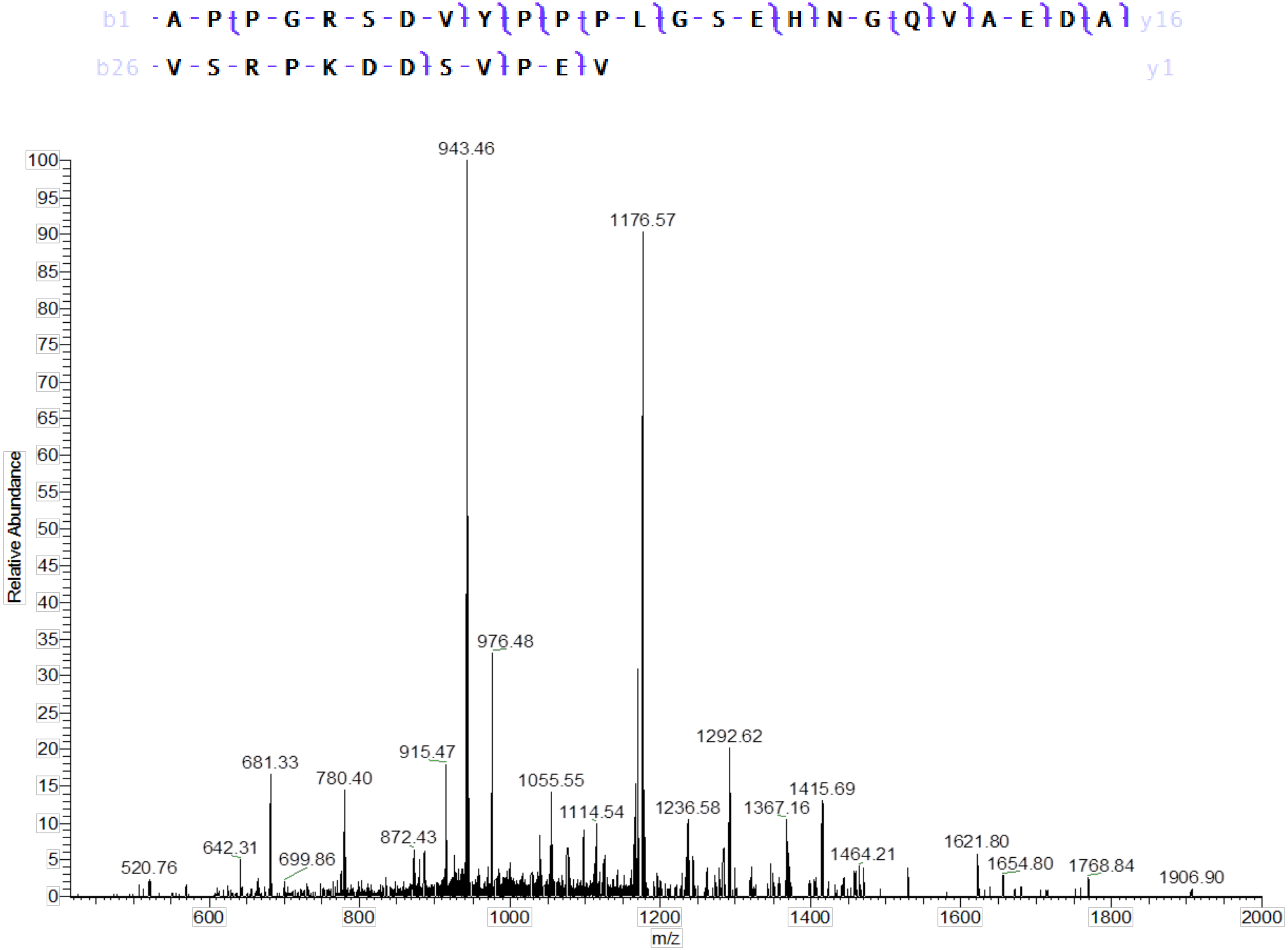

Table 2

4273	NPY_RAT	P07808	Proneuropeptide Y	4269.08	4269.08	0	C-term amidation	3.6E-42

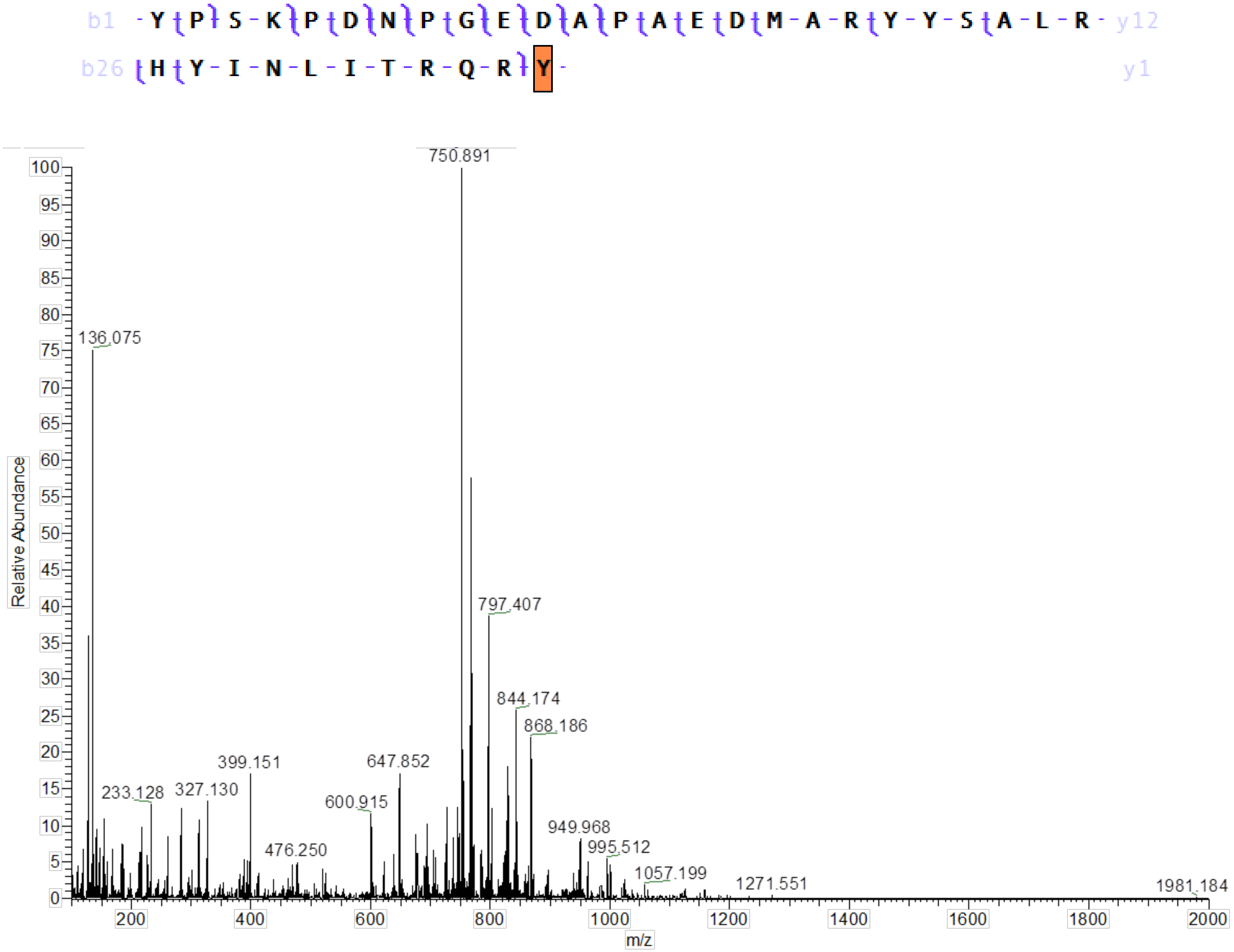

Table 2

4331	NPY_RAT	P07808	Proneuropeptide Y	4327.08	4327.09	-2		1.6E-42

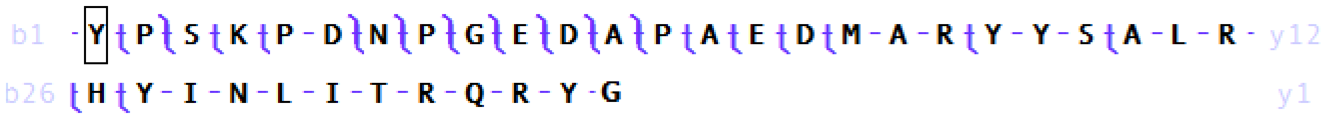

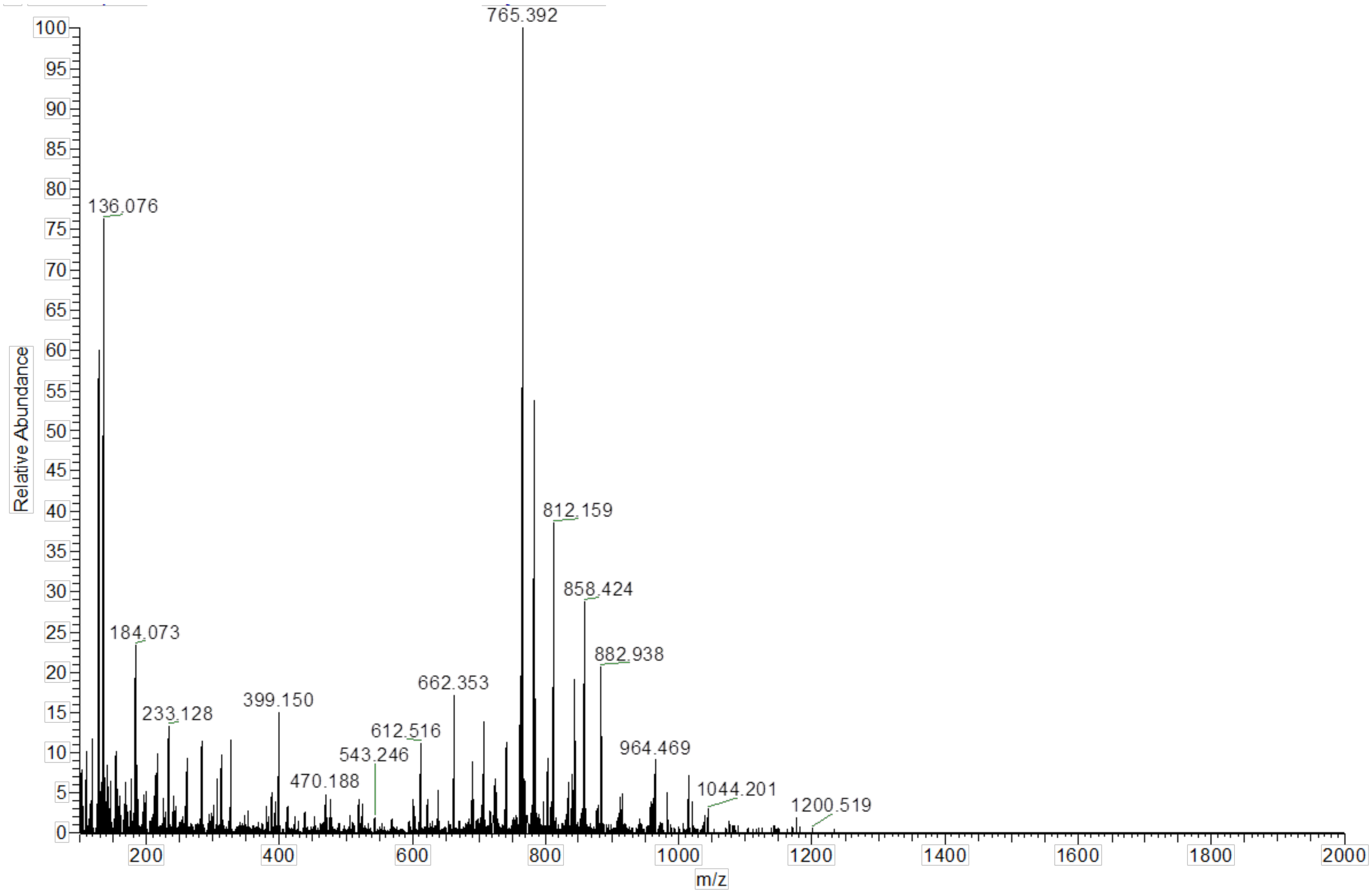

Table 2

4522	TYB10_RAT	P63312	Thymosin beta-10	4517.33	4517.33	0	N-terminal acetylation	2.2E-70

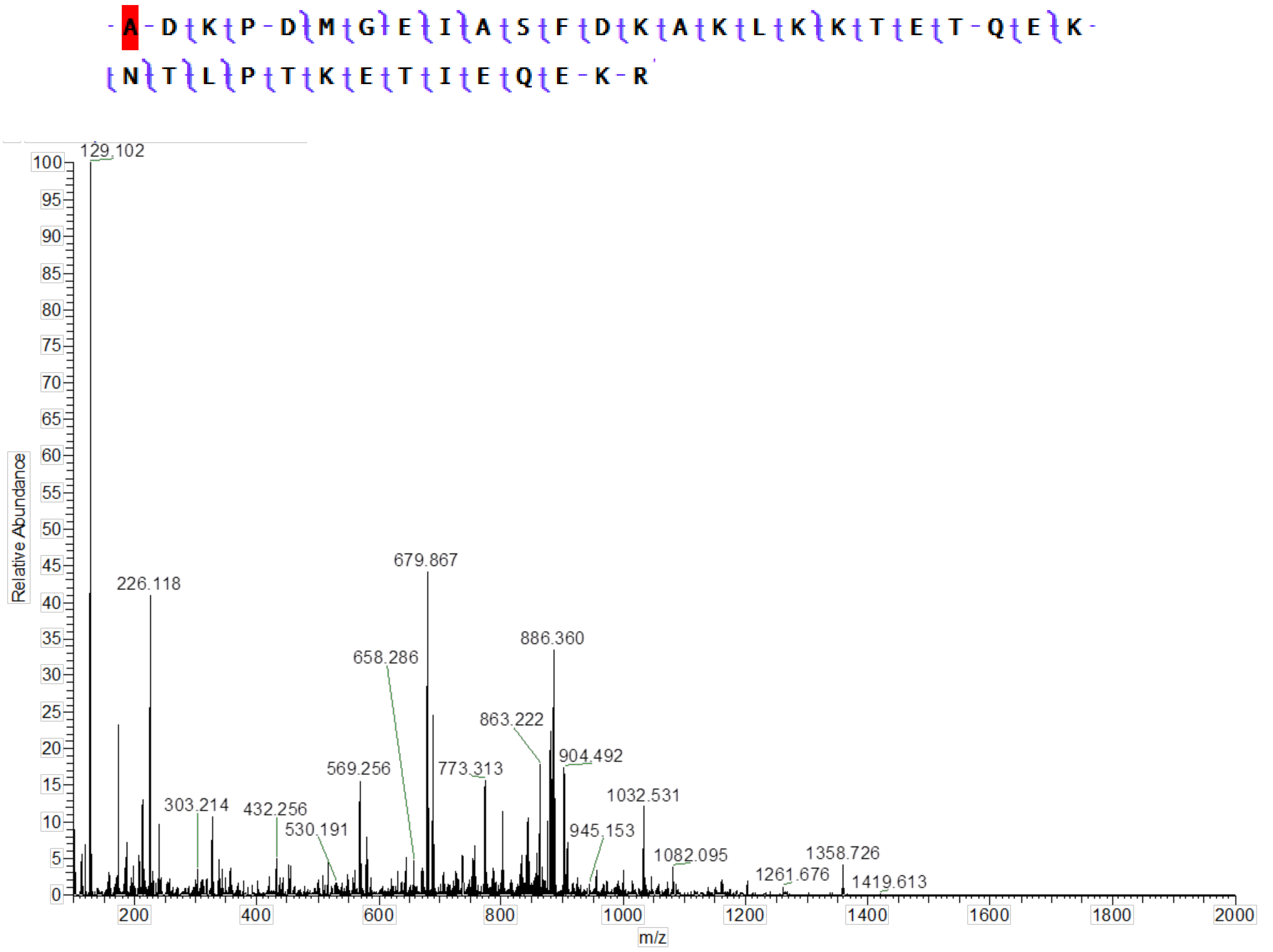

Table 2

4738	TYB10_RAT	P63312	Thymosin beta-10	4733.40	4733.41	-2	N-term acetylation	1.7E-89

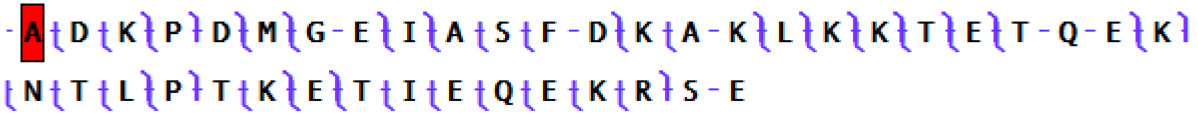

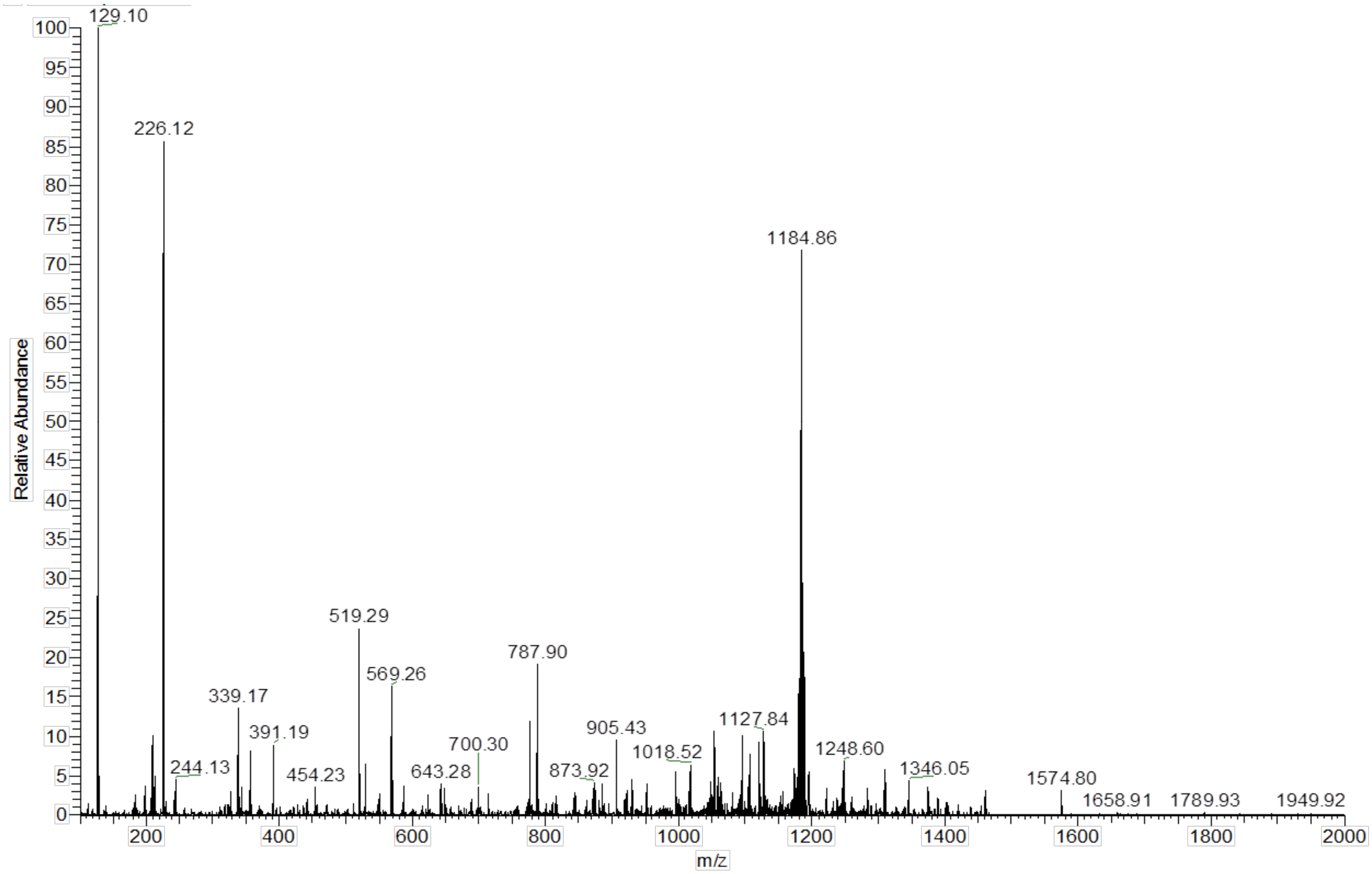

Table 2

4871	SCG2_RAT	P10362	Secretogranin-2	4867.40	4867.41	-2		1.2E-77

b1 - I P A G - S L - K N E D T P - N - R - Q - Y L D E D M L L K V y18
b26 L E Y L N Q E Q A - E Q G - R - E H L - A - y1

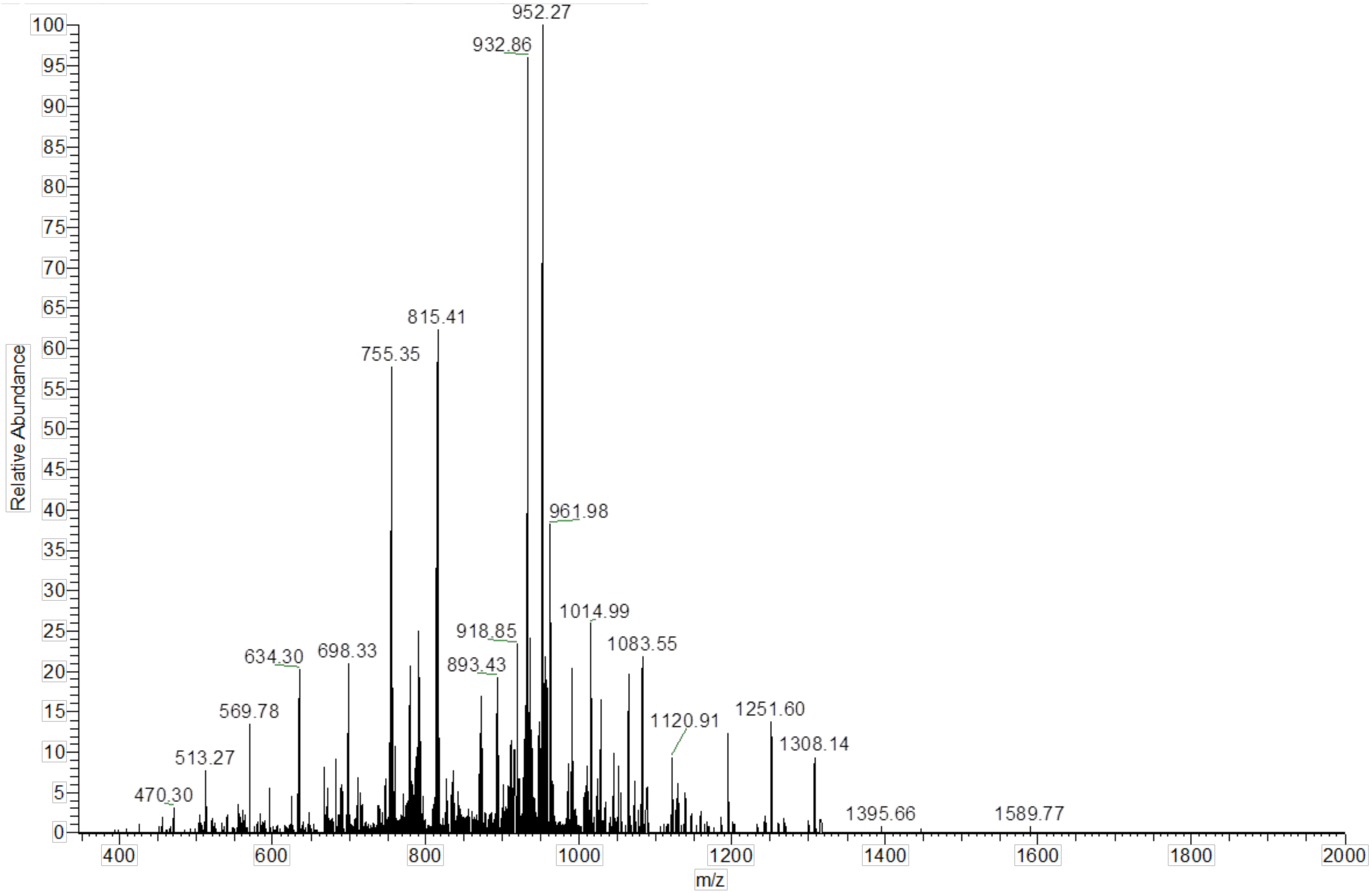

Table 2

7114	FILUV9_RAT	FILUV9	Uncharacterized	7108.52	7108.51	1		4.9E-42

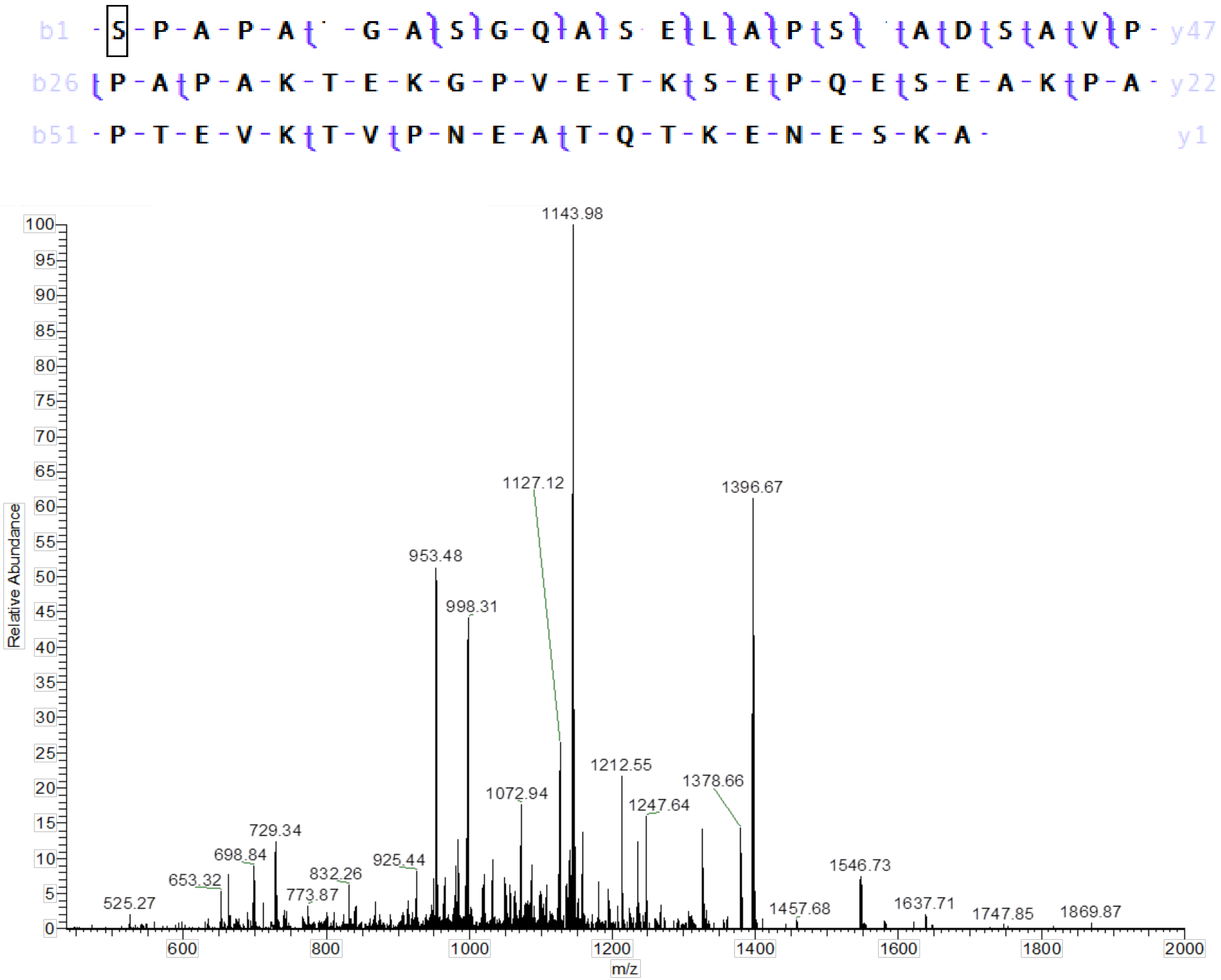

Table 2

8468	UBB_RAT	P0CG51	Polyubiquitin	8461.56	8461.57	-1	Oxidation	2.9E-35

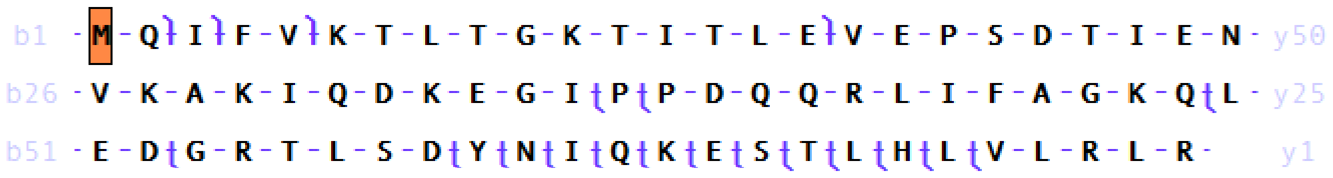

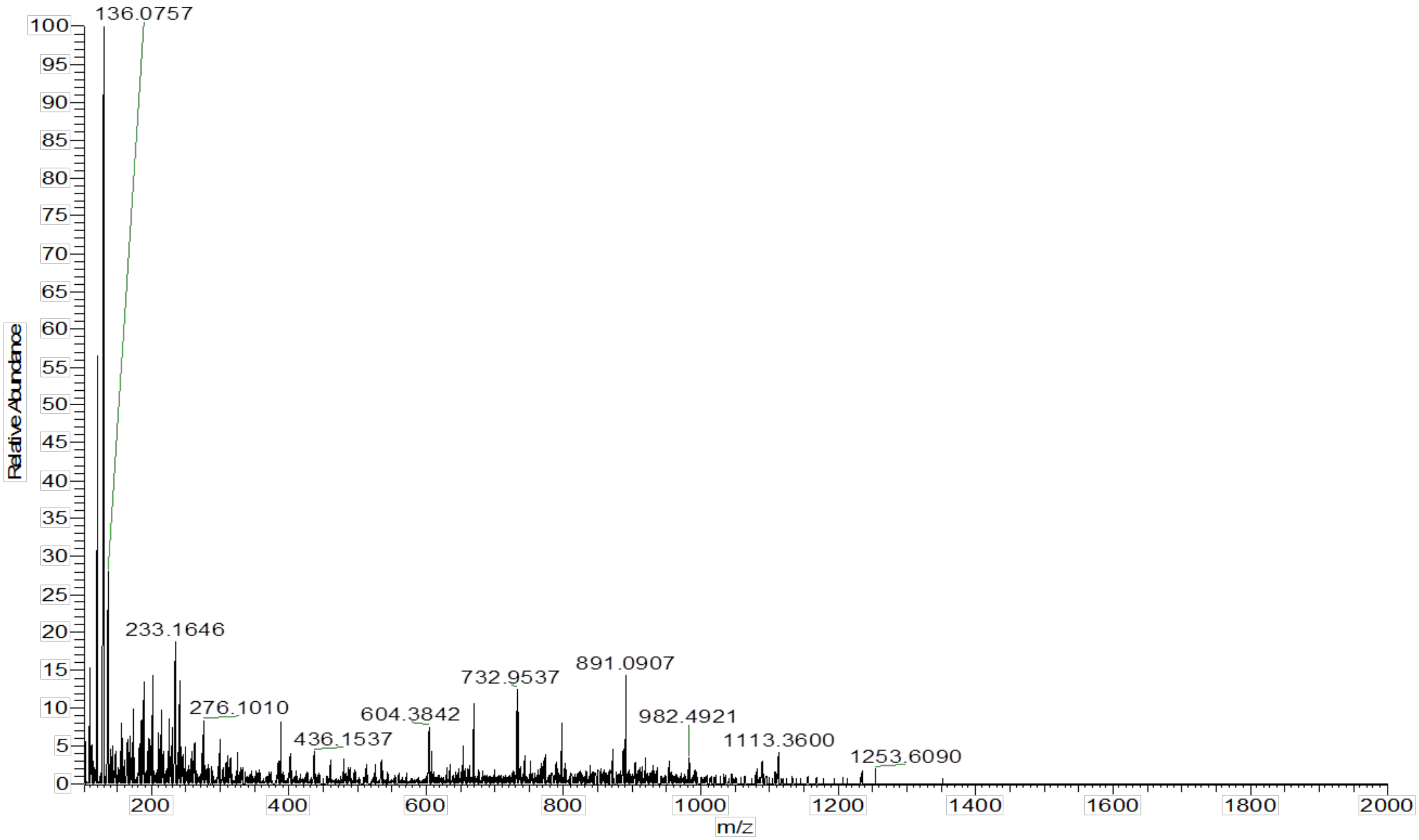

Table 2

8928	ATP5J_RAT	P21571	ATP synthase coupling factor 6	8921.55	8921.55	0		1.7E-52

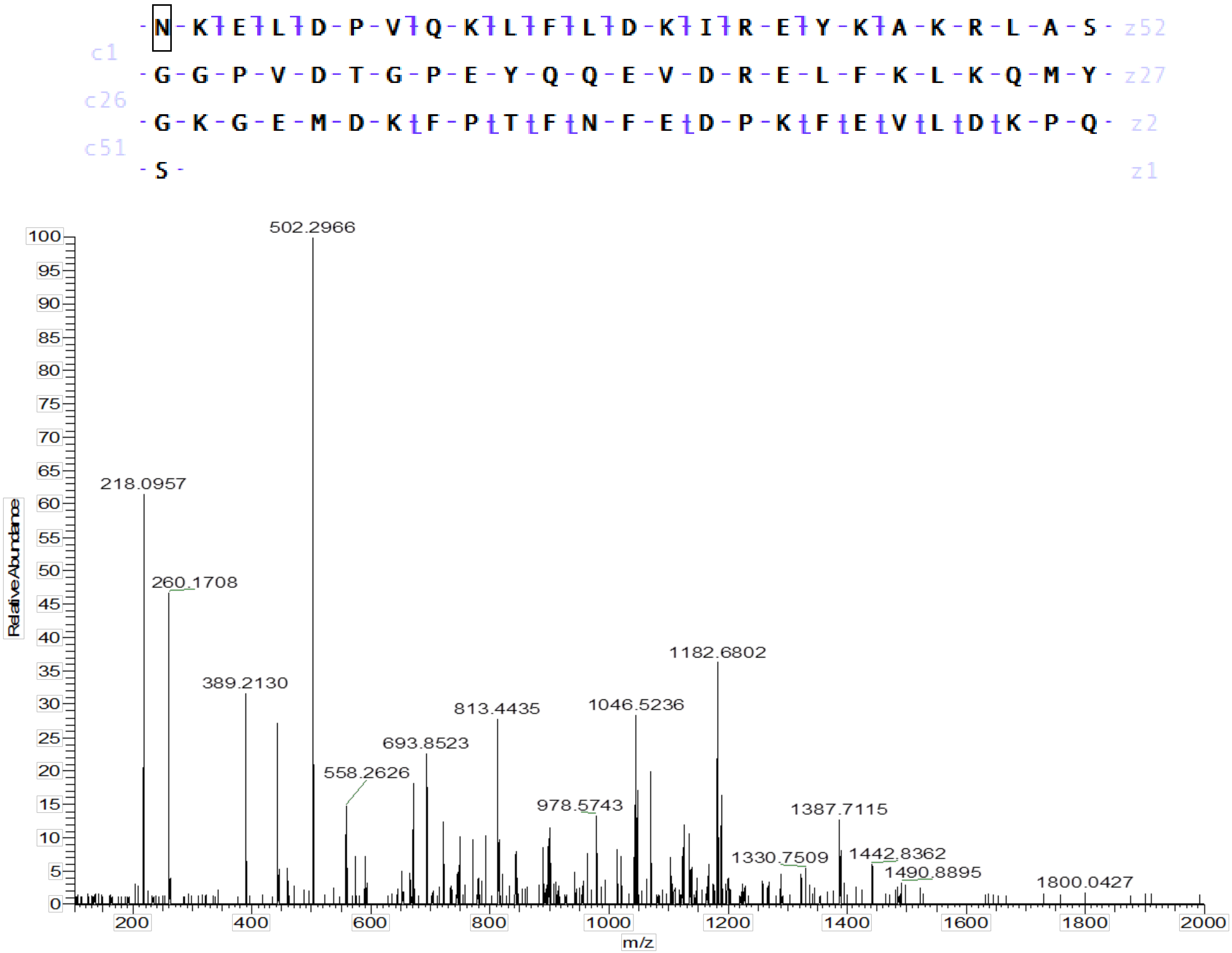

Table 2

10918	DLRB1_RAT	P62628	Dynein light chain roadblock-type 1	10909.71	10909.72	-1	N-term acetylation, unfixed oxidation	6.2E-36

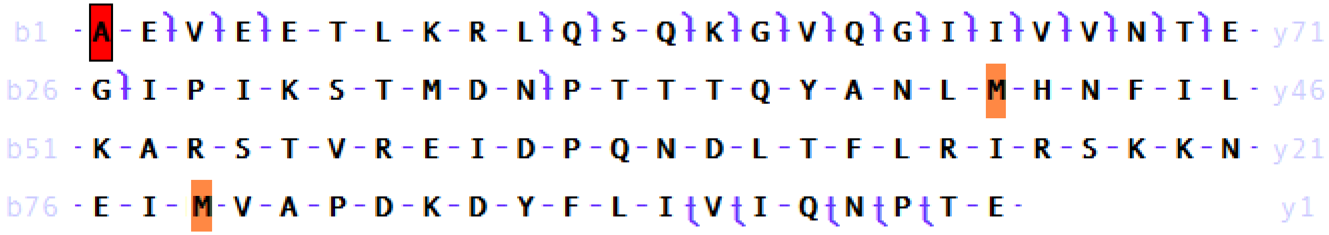

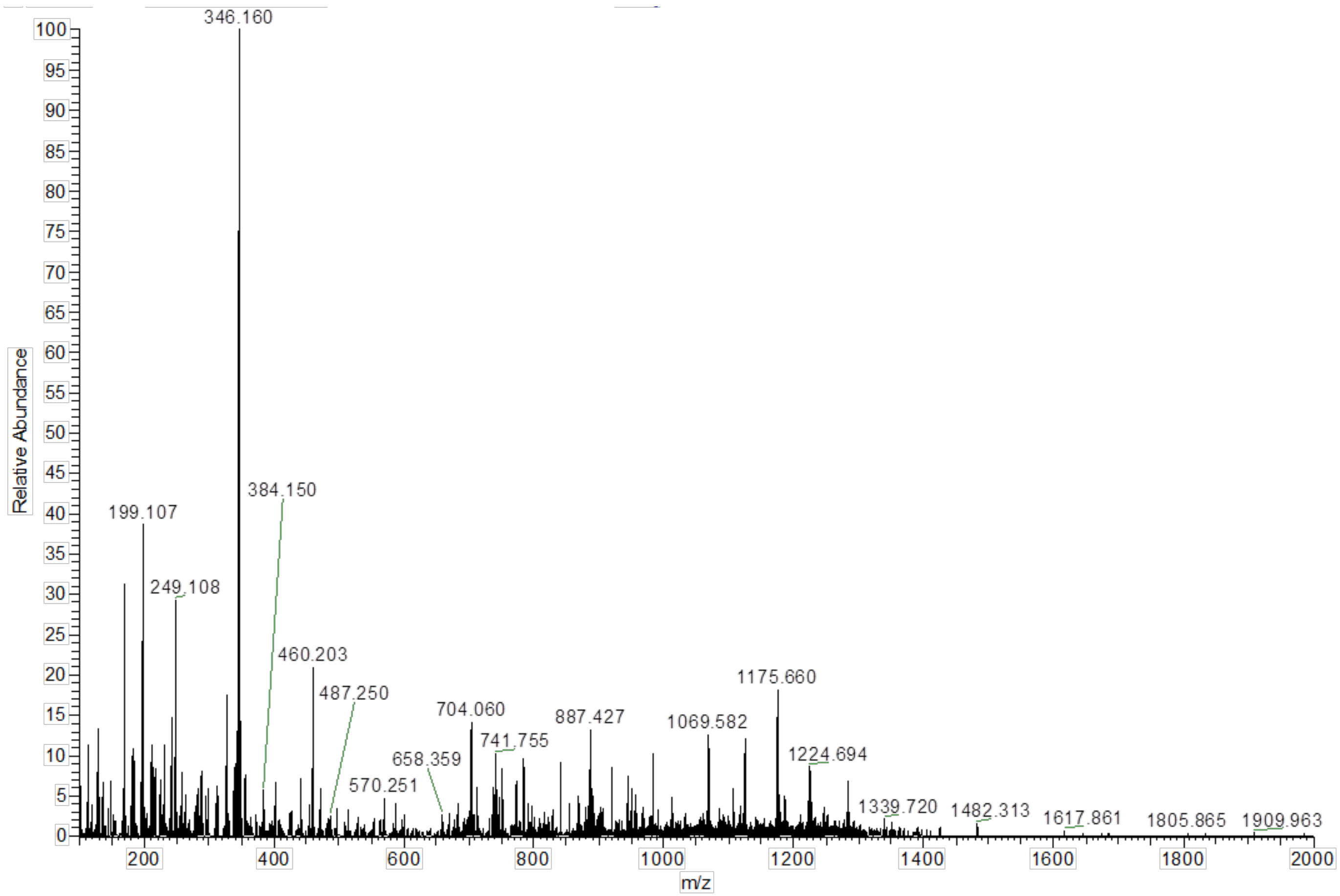

Table 2

12204	ARP19_RAT	Q712U5	cAMP-regulated phosphorylation 19	12196.24	12196.26	-2	N-term acetylation	2.3E-13

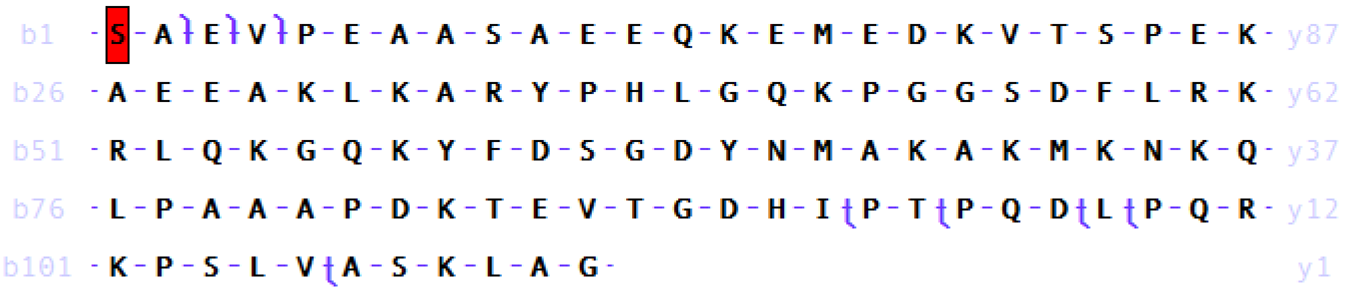

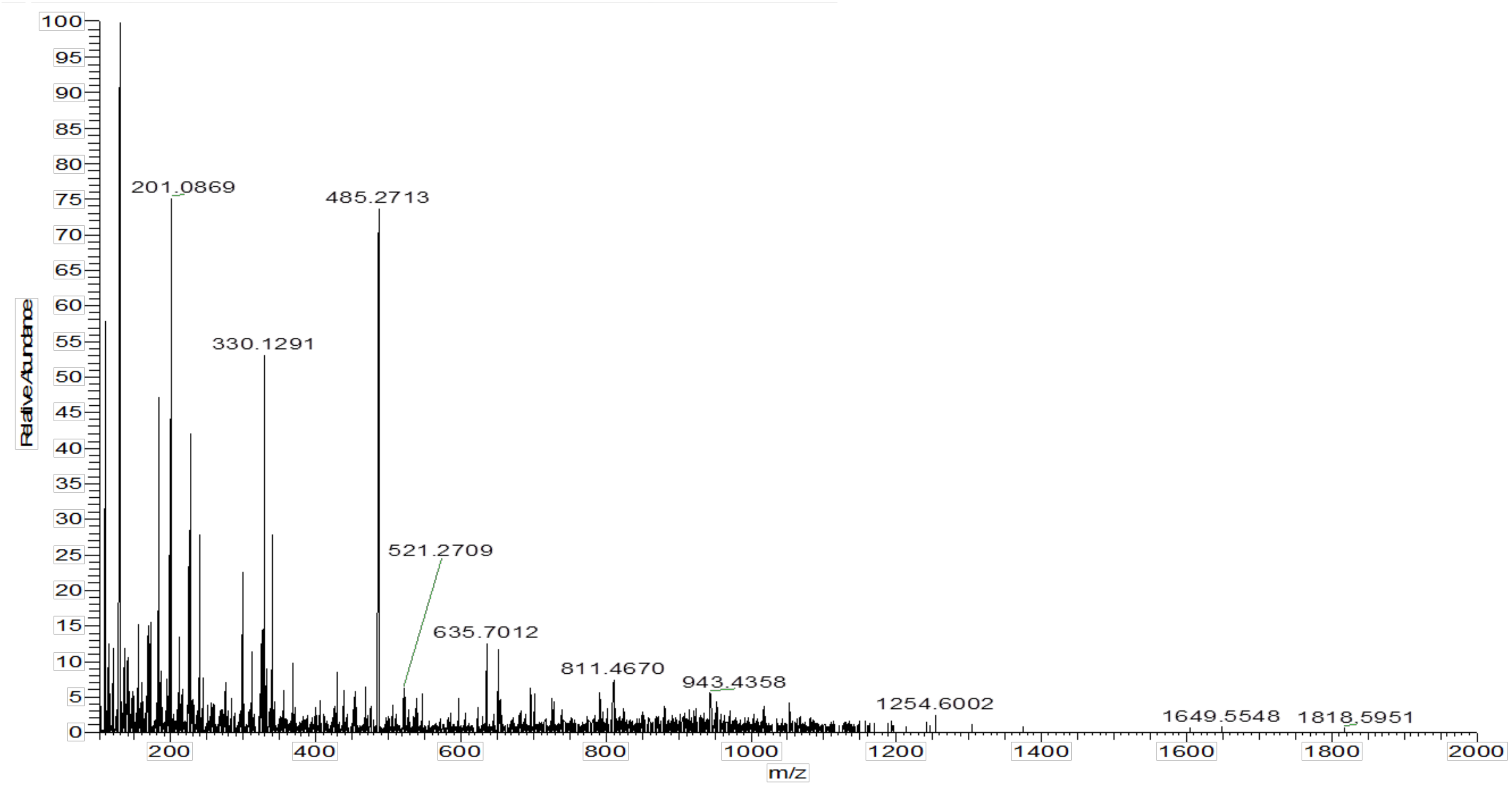

Table 2

14428	Q6MGC4_ RAT	Q6MGC4	H2-K region expressed gene 2, rat orthologue	14417.76	14417.73	2	N-term acetylation, H_2O adduct	3.1E-24

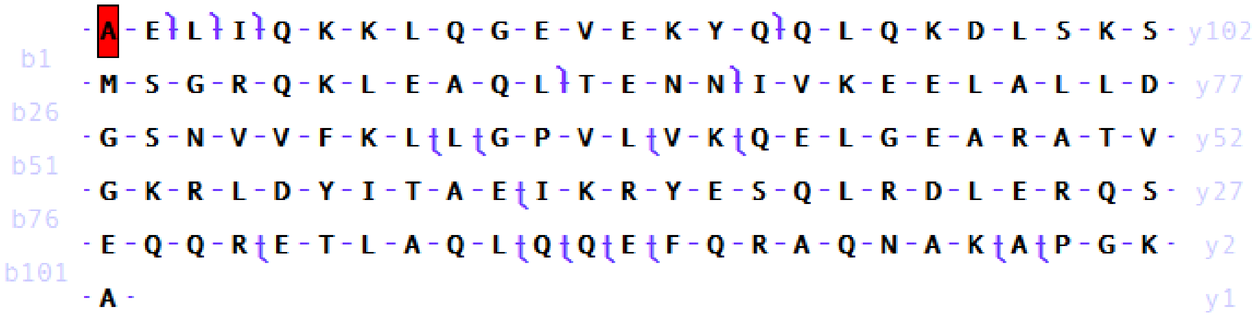

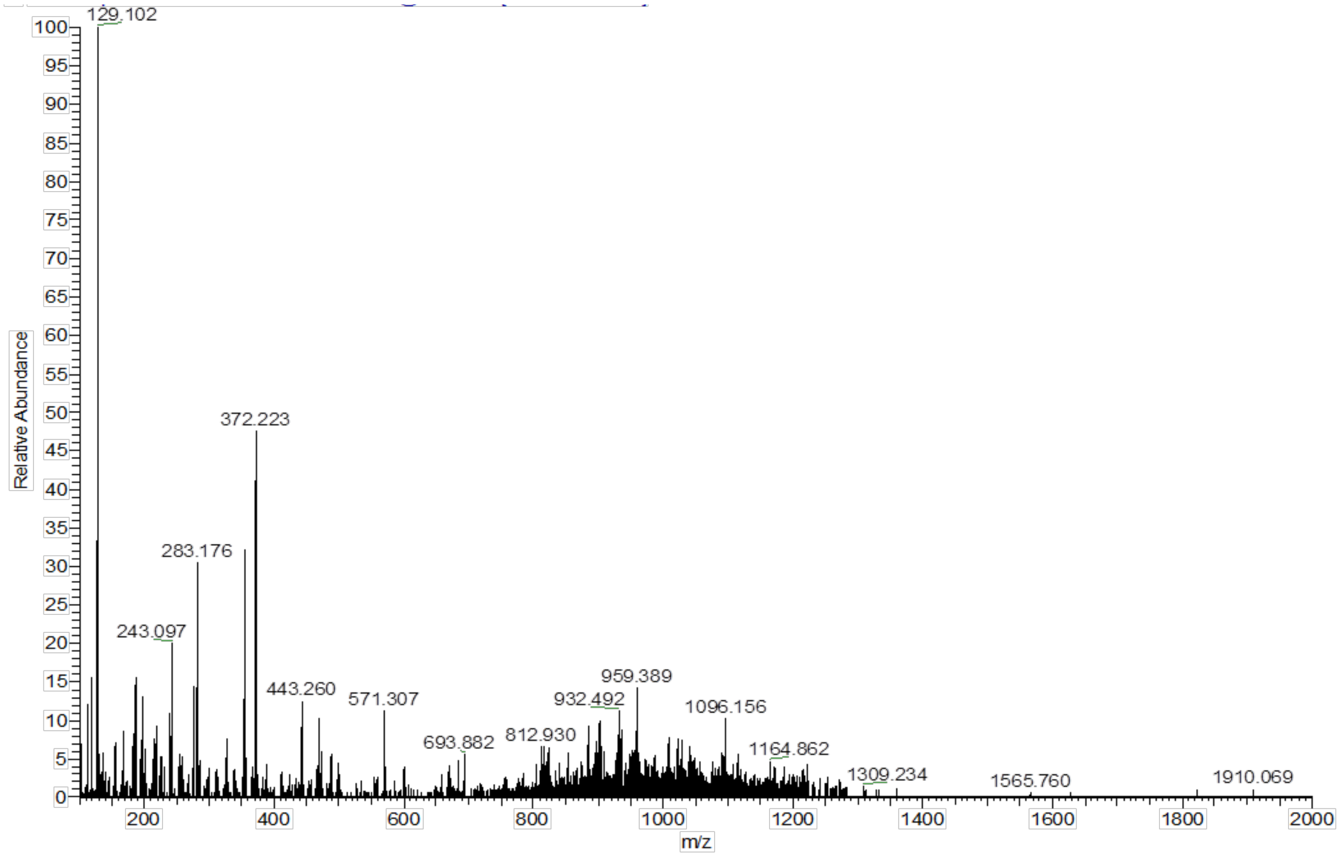

Supplemental information of the proteins identified from nanoLC-ESI-LTQ-Orbitrap ELITE in **Table S2**. The protein identities are sorted in the order of their monoisotopic mass. The protein information and resulting MS/MS spectra are shown in the fashion as below:

Entry	Accession	Protein description	Calc'd mass (Da)	Exp'd mass (Da)	Mass difference (ppm)	PTM	E-value

Protein sequence, coverage and assigned PTMs

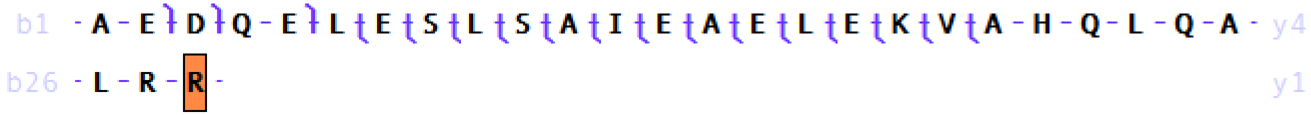

The MS/MS spectrum that corresponds to the sequence assignment is shown. The resulting fragment ions are listed in the excel sheets.

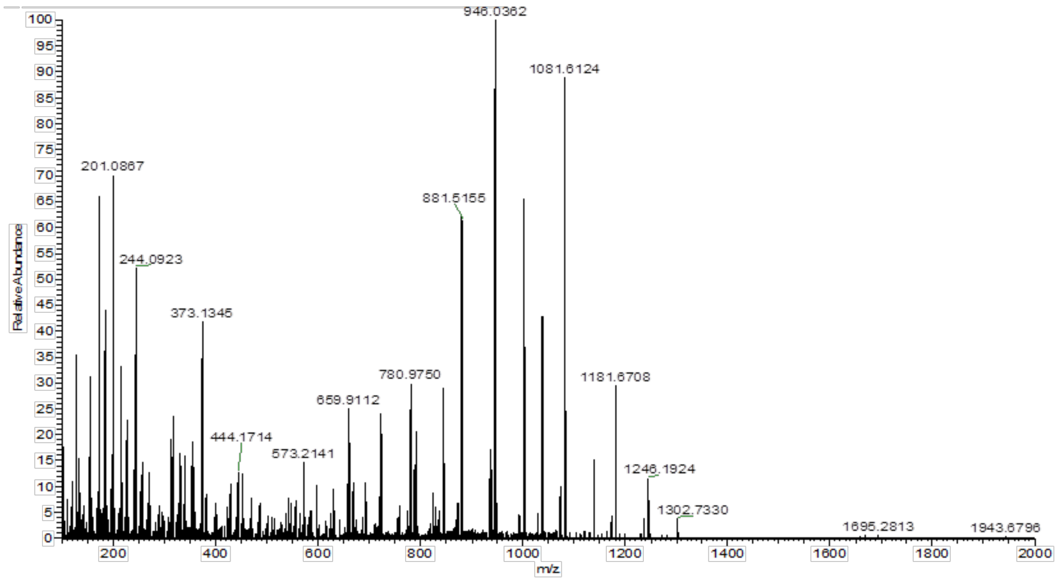

Table S2

CMGA_RAT	P10354	Chromagranin-A	3174.66	3174.66	0	C-term amidation	6.8E-34

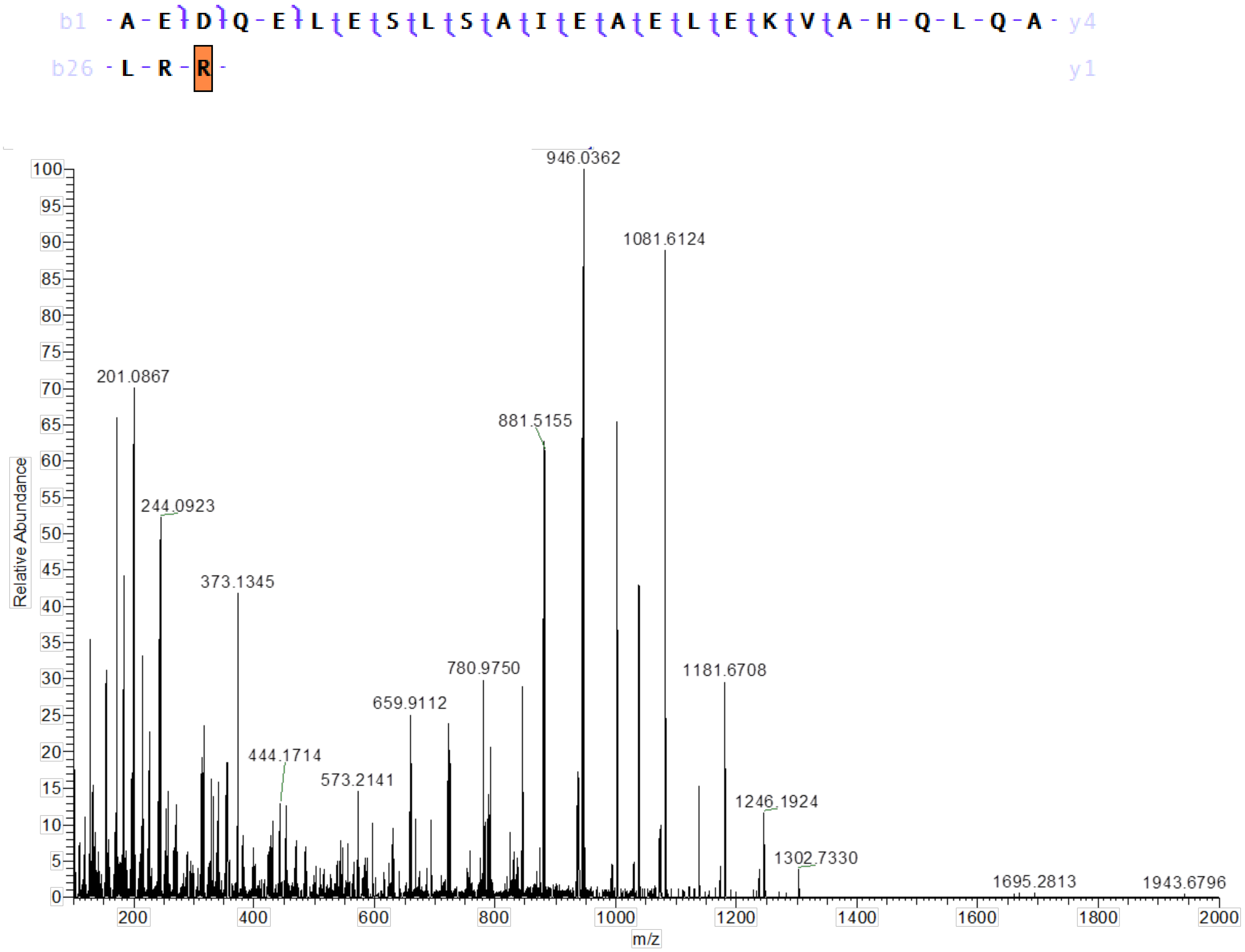

Table S2

CCKN_RAT	P01355	Cholecysokinin	3392.80	3392.81	-3		2.5E-13

b1 -Y I Q Q-V-R-K-A P-S-G-R-M-S-V-L K N-L-Q G-L D P S- y15

b26 H R-I-S-D y1

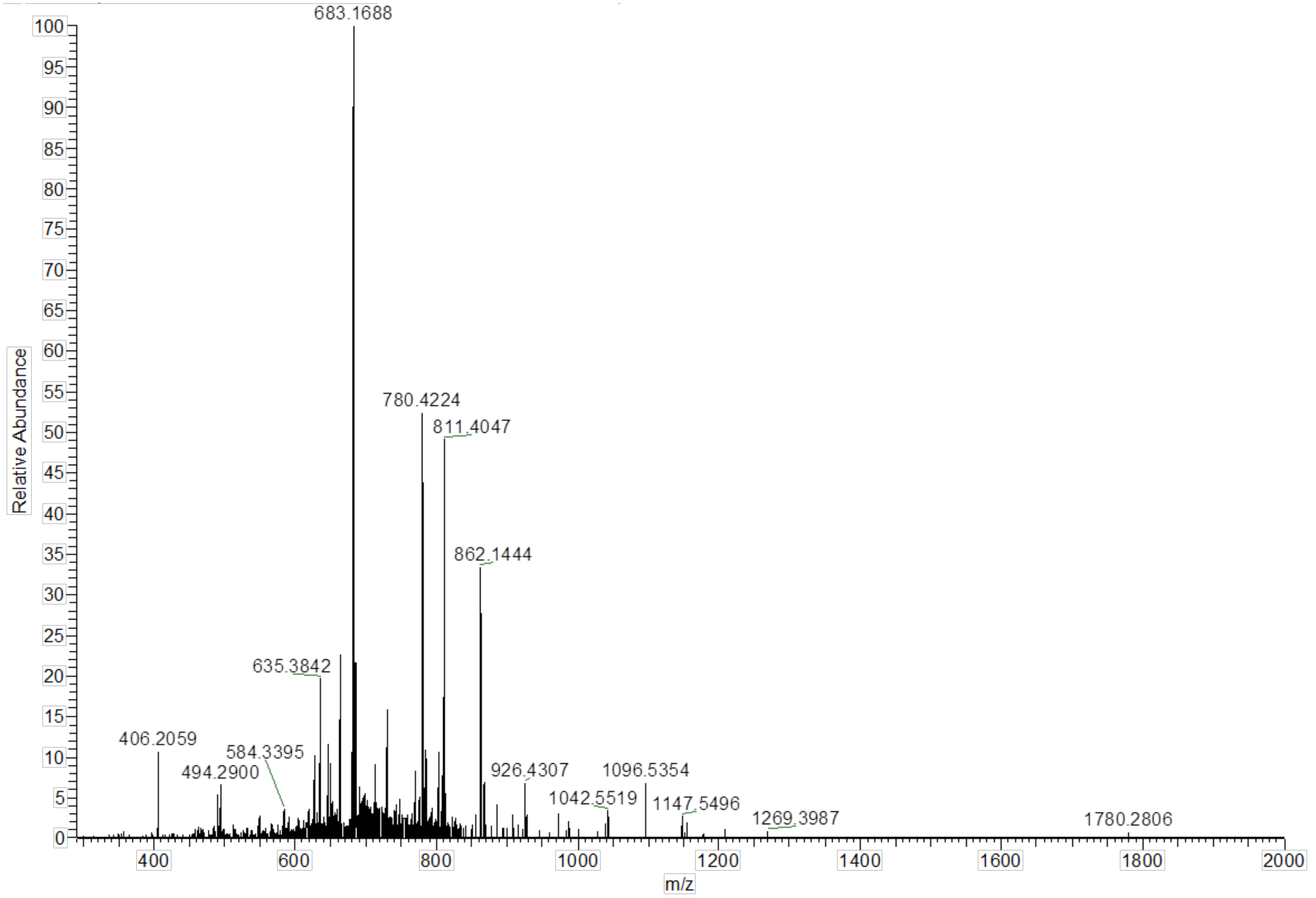

Table S2

F1LP33_RAT	F1LP33	Oxysterol-binding protein	3679.89	3679.87	5		1.2E-06

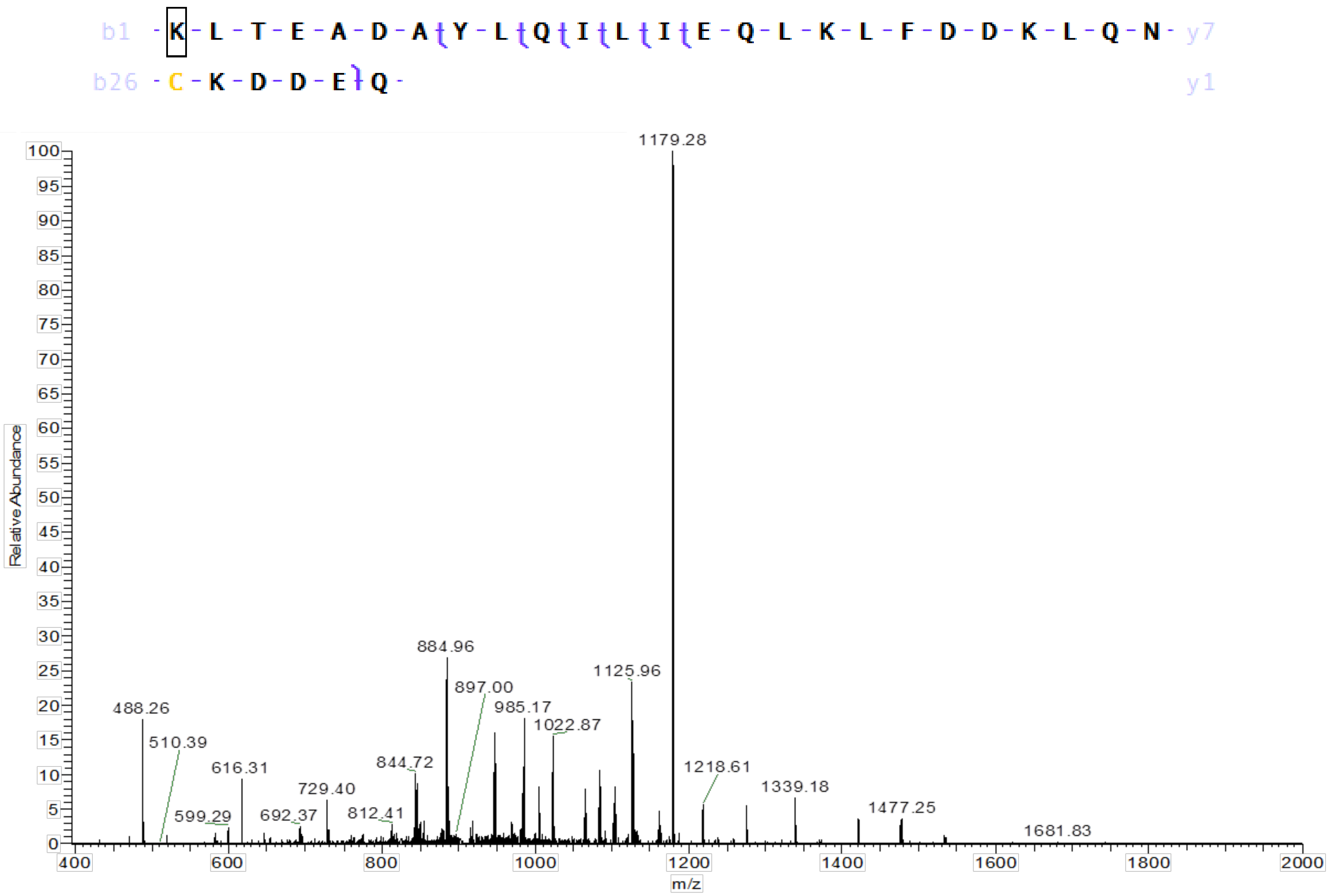

Table S2

KCRU_RAT	P25809	Creatine kinase U-type	3811.23	3811.23	0	N-term acetylation	4.2E-57

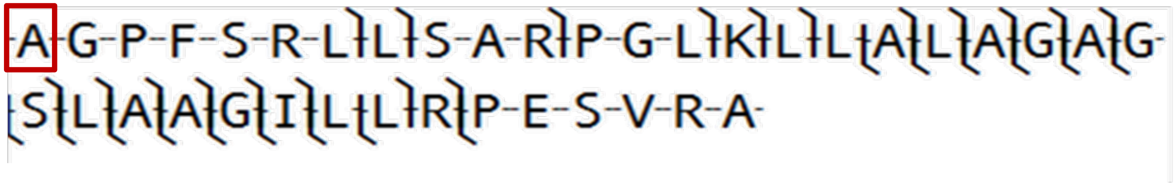

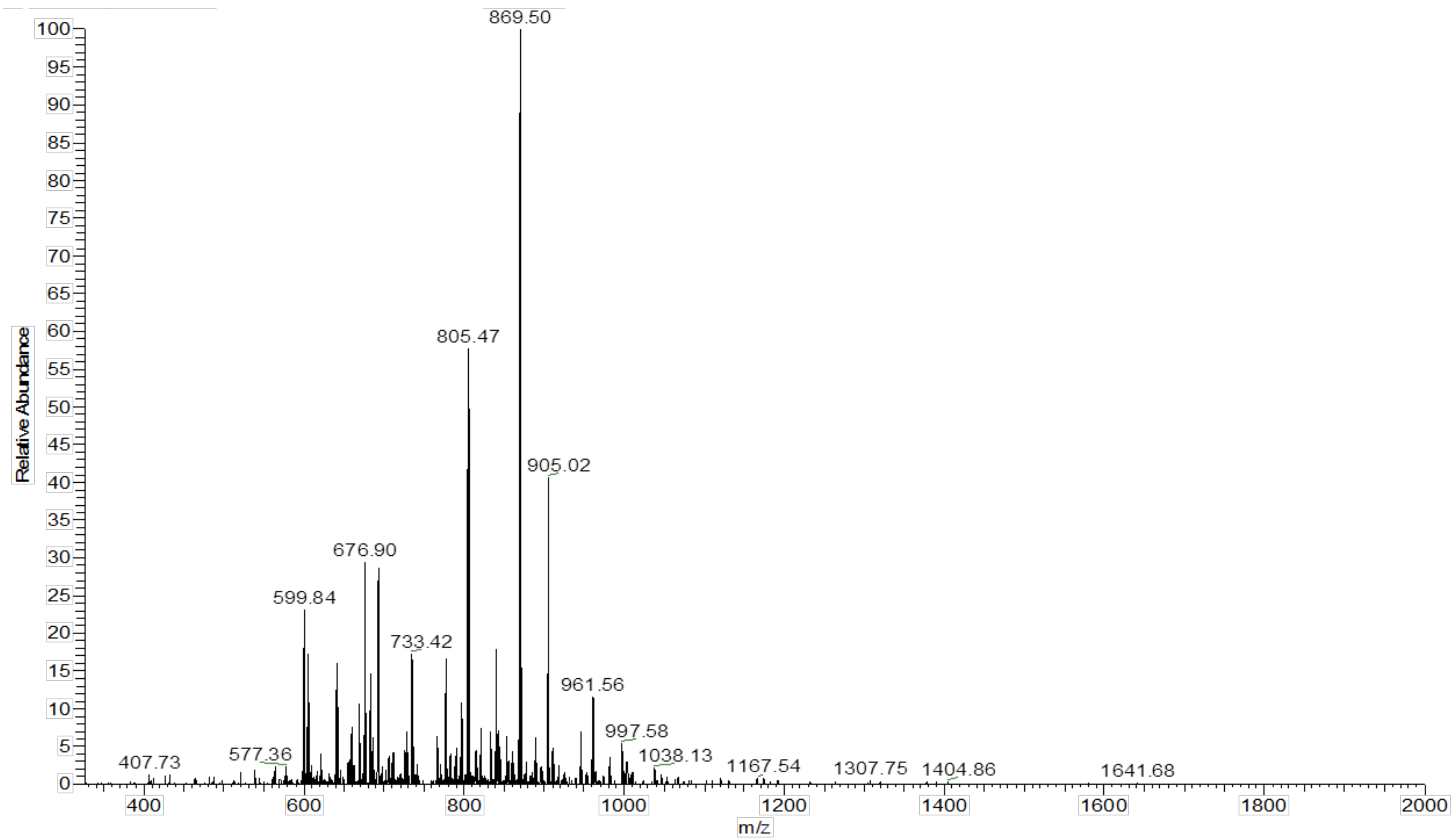

Table S2

MDHM_RAT	P04636	Malate dehydrogenase	3832.09	3832.09	0		7.0E-50

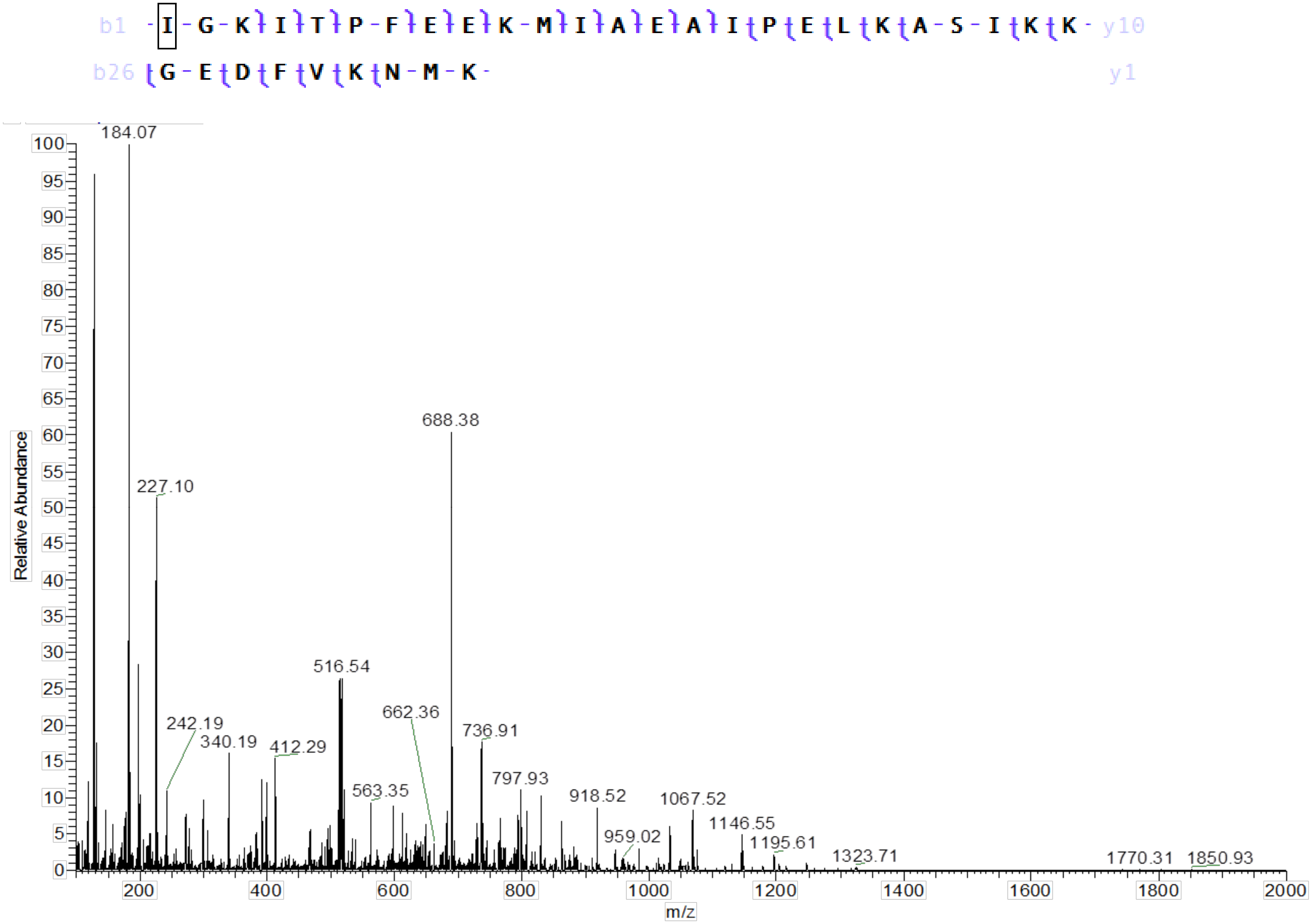

Table S2

D4AAI2_RAT	D4AAI2	Histocompatibility (minor) HA-1, isoform CRA_b	3975.99	3975.99	0		8.2E-05

b1 - F - Y - H - E - L - V - G - L - A - K - D - S - L - K - A - E - A - E - A - K - A - A - S - R - G - y14

b26 - R - Q - D - G - S - E - S - E - A - A - T - L - A - y1

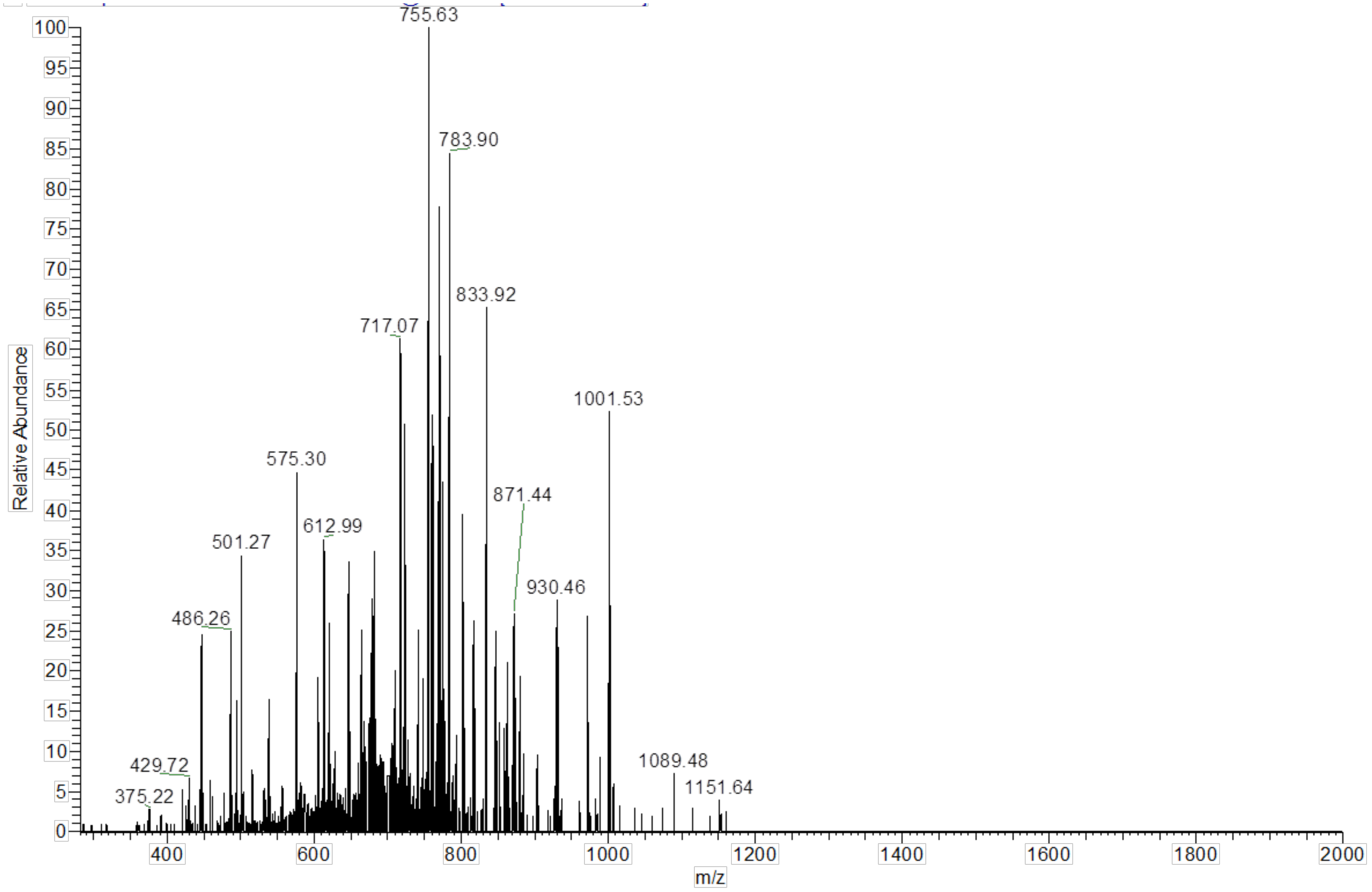

Table S2

D3ZMX3_RAT	D3ZMX3	Neutralized-like (drosophila), isoform CRA_b	4008.92	4008.91	2		3.3E-07

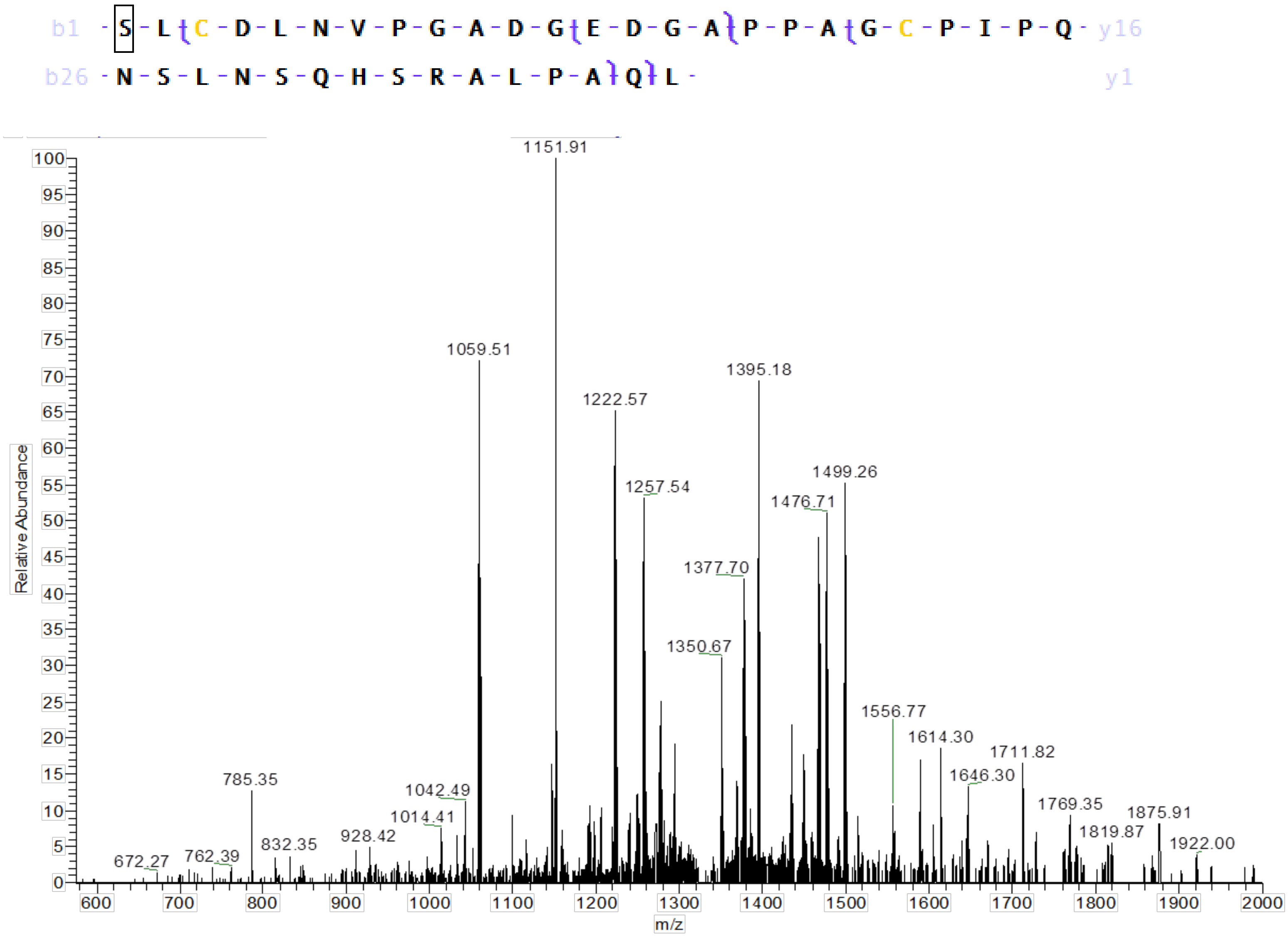

Table S2

TMM35_RAT	Q6JAM9	Transmembrane protein 35	4036.15	4036.16	-2		1.2E-36

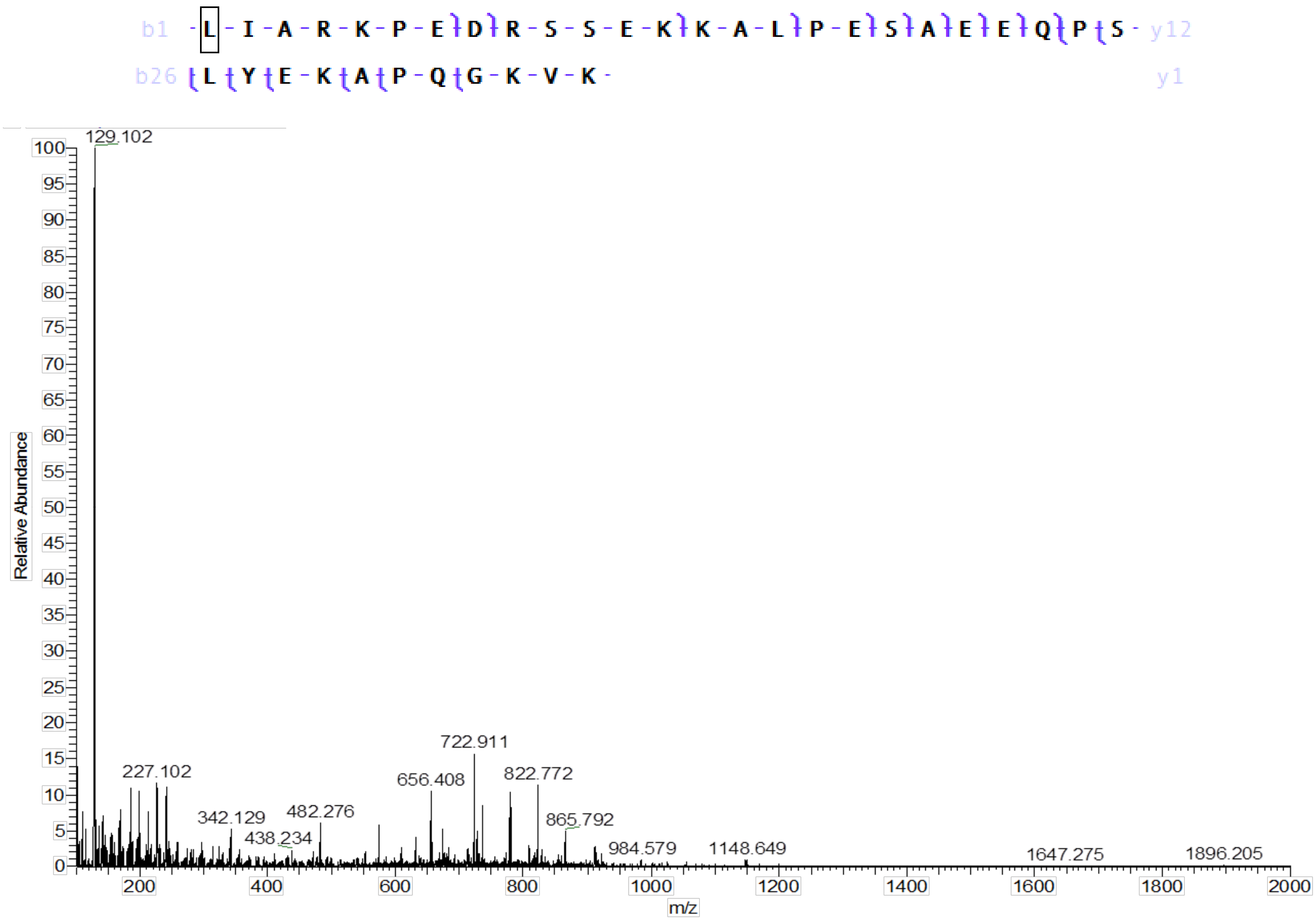

Table S2

NPY_RAT	P07808	Proneuropeptide Y	4285.07	4285.08	-2	Oxidation, C-term amidation	6.2E-34

b1 Y P S K P D N P G E D A P A E D M A R Y Y S A L R y12
b26 H Y I N L I T R Q R Y y1

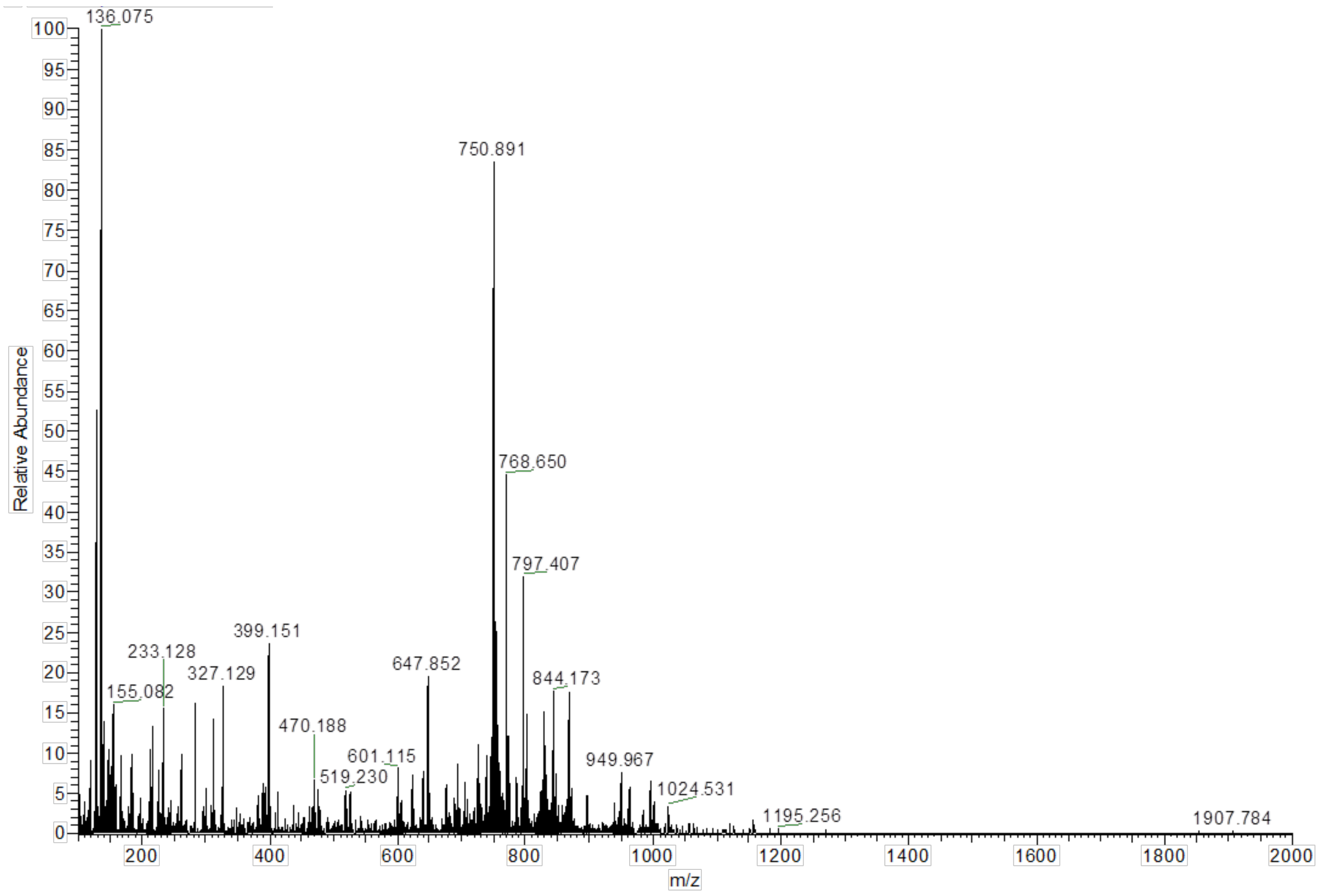

Table S2

CART_RAT	P49192	Cocaine- and amphetamine- regulated transcript protein	4384.02	4384.03	-2	3*Disulfide bonds	1.3E-11

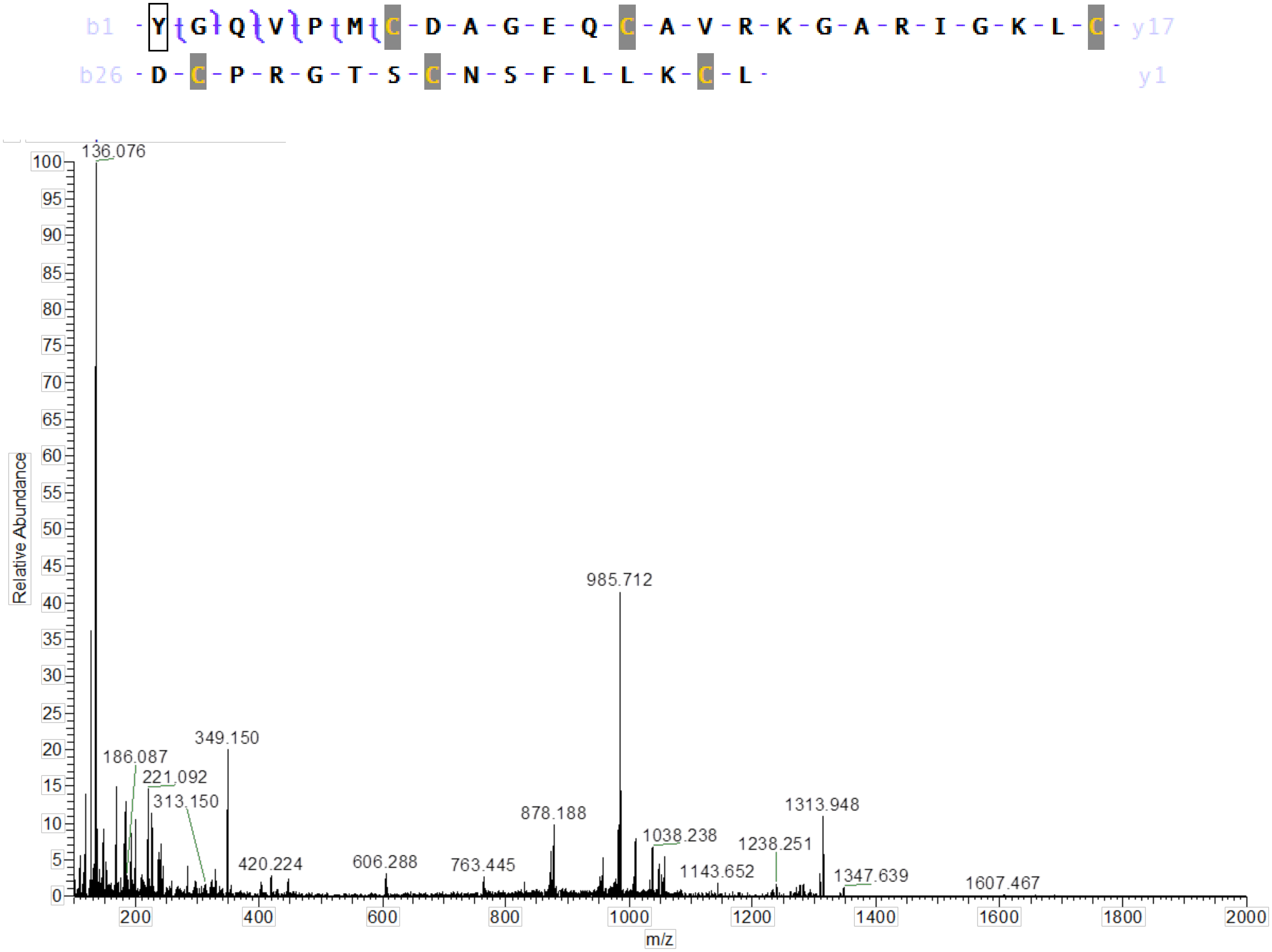

Table S2

TYB10_RAT	P63312	Thymosin beta-10	4531.34	4531.35	-2	N-term acetylation, C-term methylation	1.7E-69

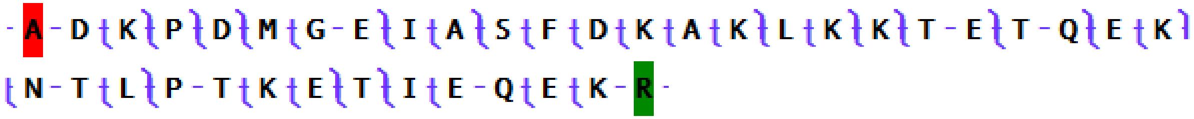

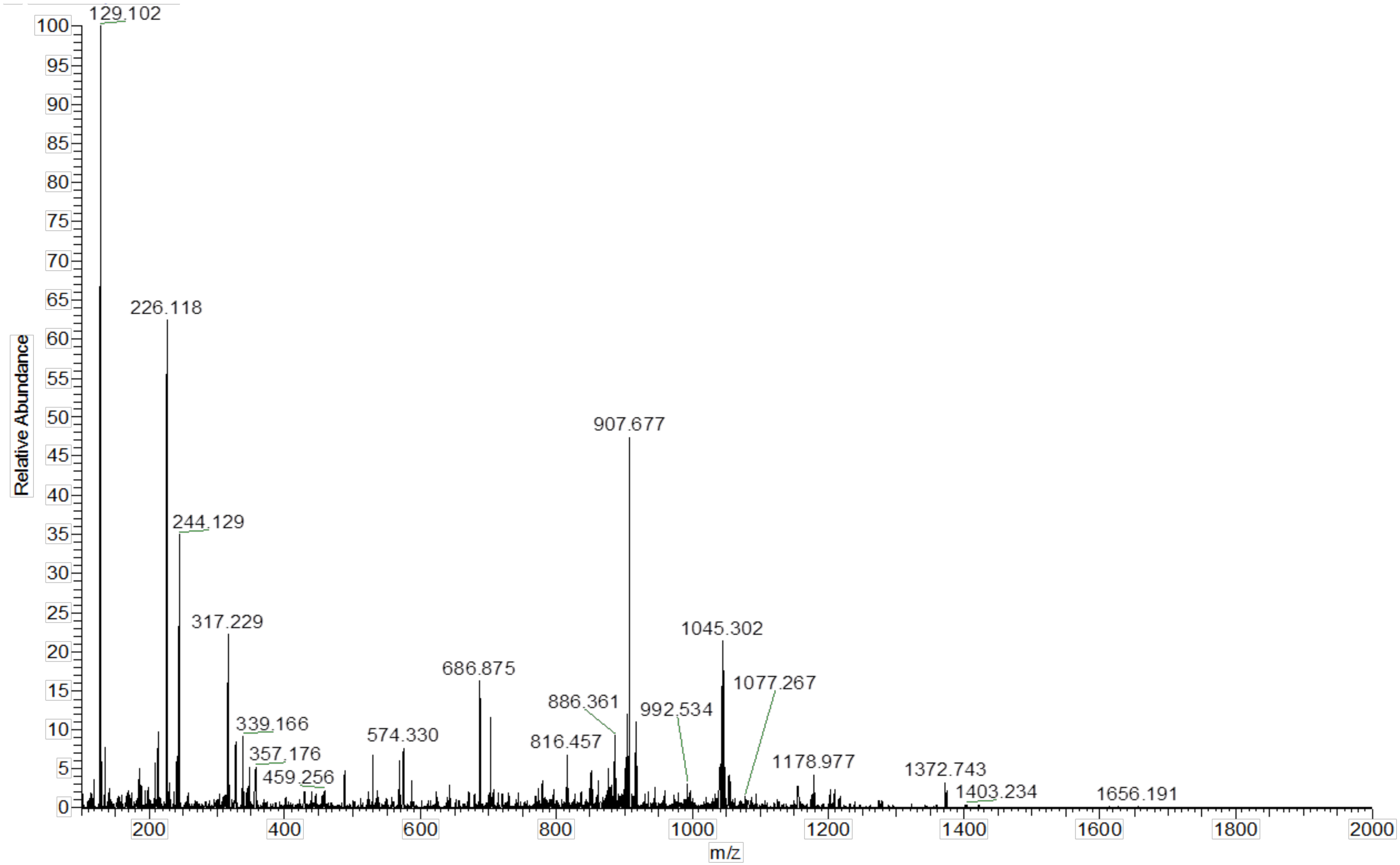

Table S2

NCAM1_RAT	P13596	Neural cell adhesion molecule 1	4548.23	4548.23	0		7.5E-41

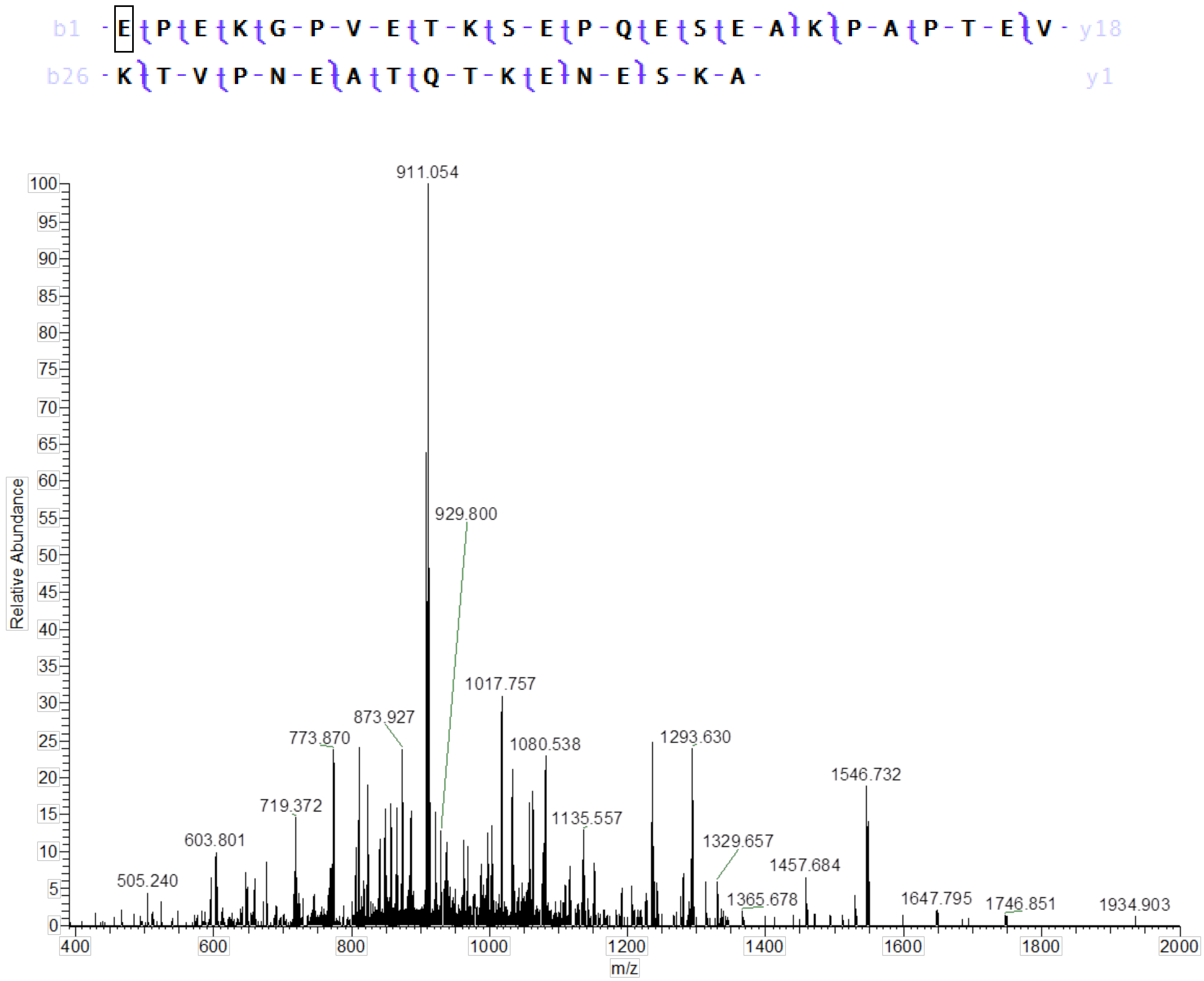

Table S2

TYB4_RAT	P62329	Thymosin beta-4	4616.35	4616.35	0	N-term acetylation	5.0E-62

-S-D K P-D M-A-E I E K F-D K-S-K L K K T-E T-Q E K N P L P-S-K E T I E Q-E K Q

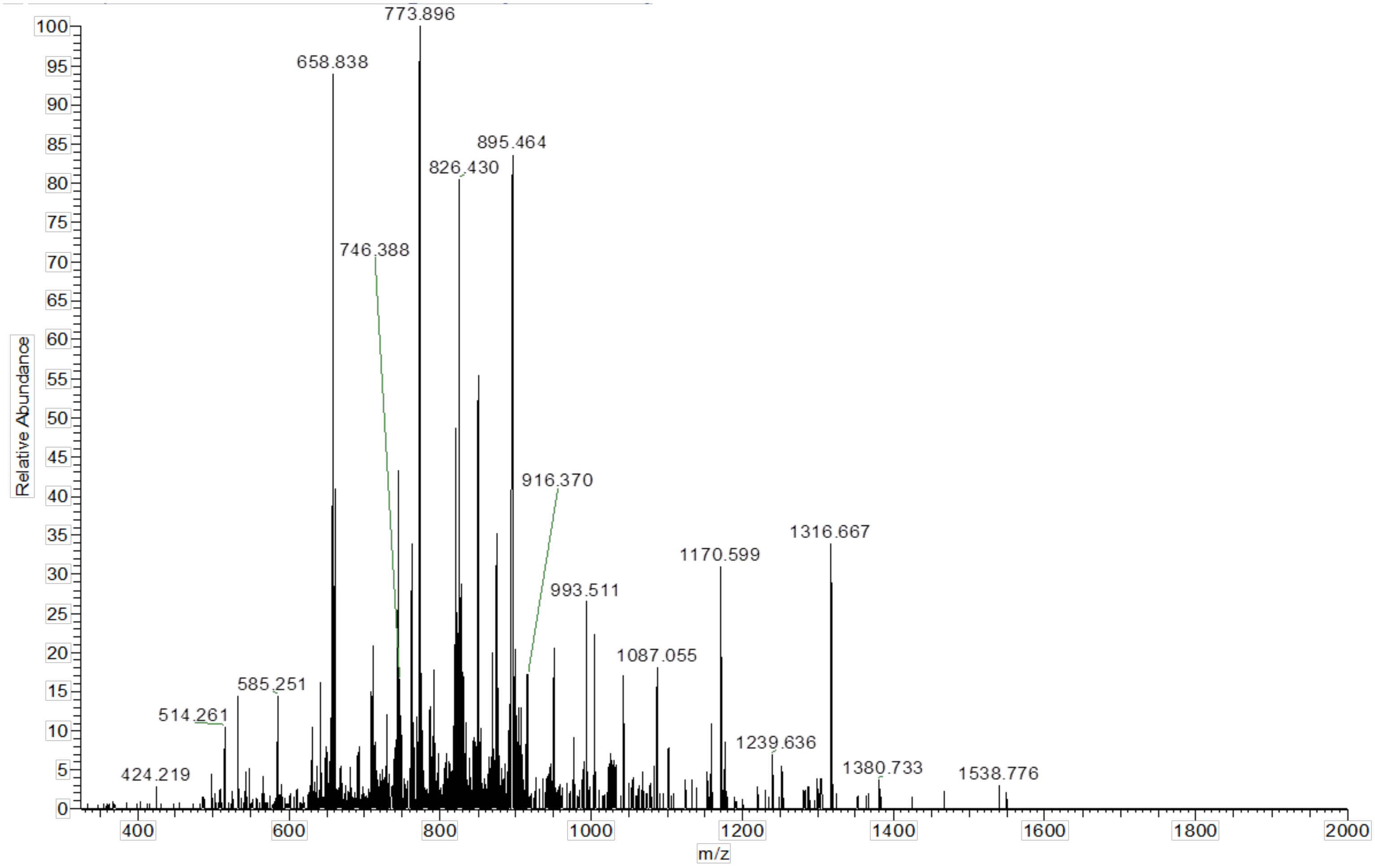

Table S2

TYB10_RAT	P63312	Thymosin beta-10	4620.36	4620.36	0		9.4E-13

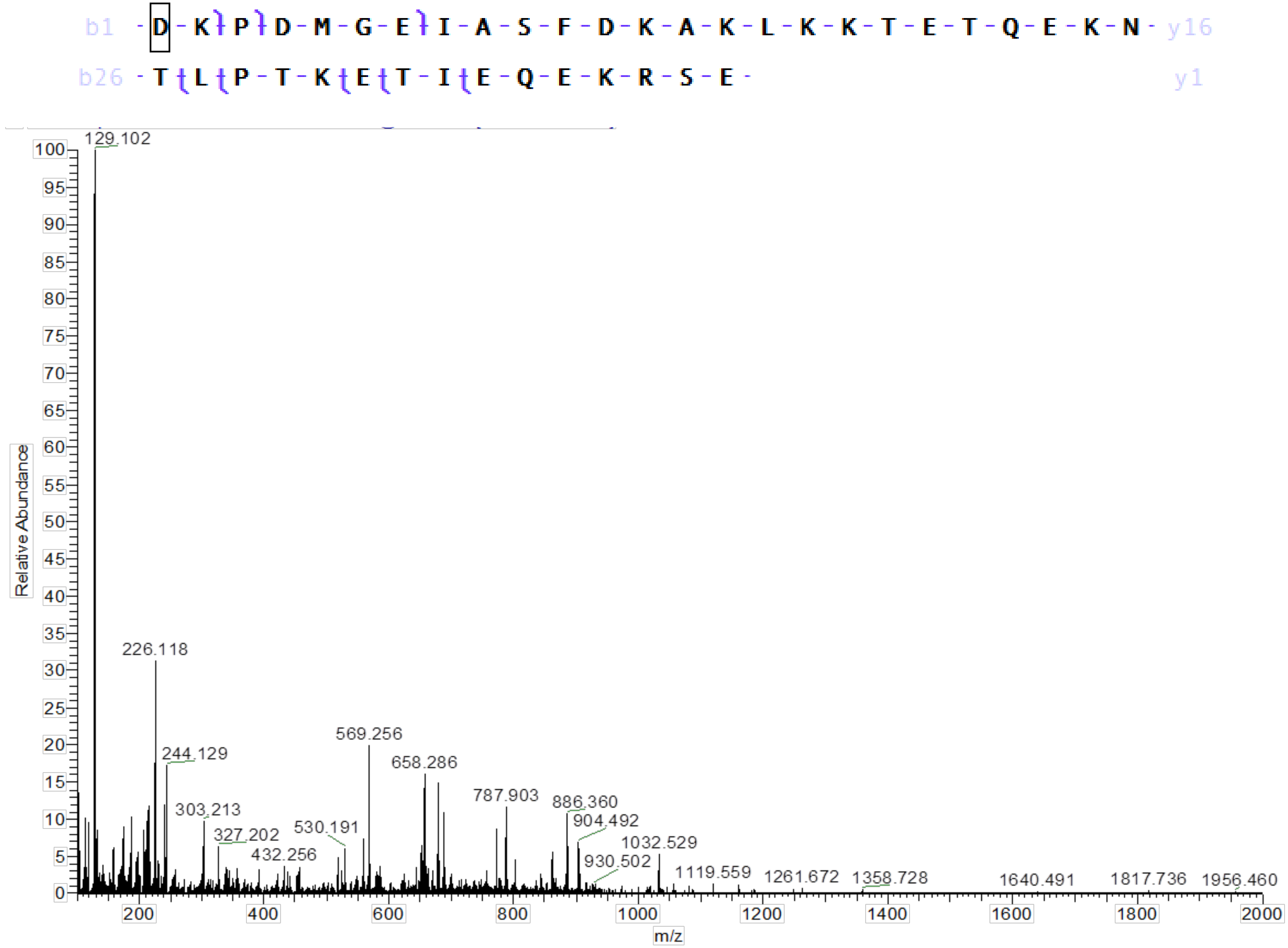

Table S2

SCG2_RAT	P10362	Secretogranin-2	4683.28	4683.29	-2		2.1E-70

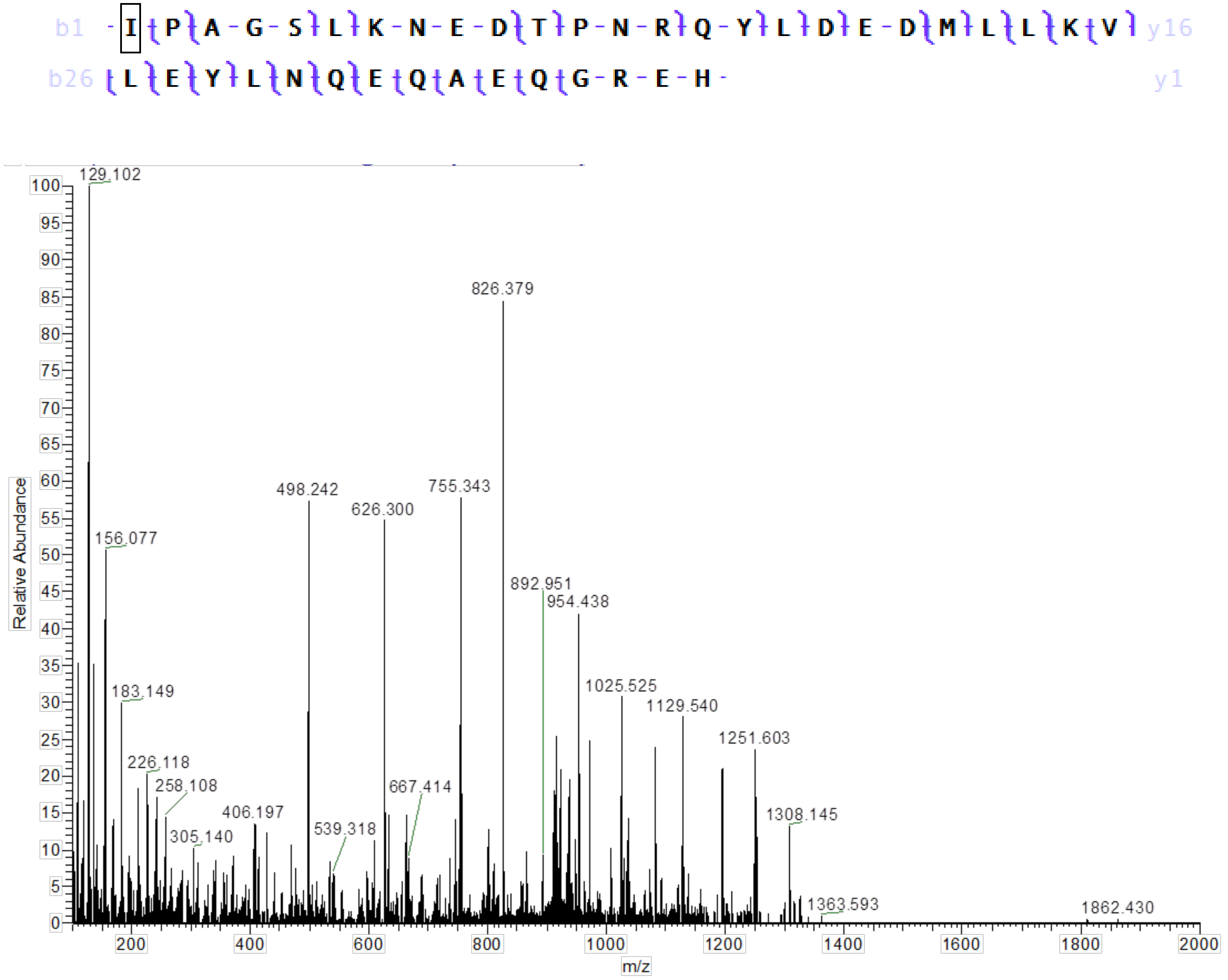

Table S2

TYB4_RAT	P62329	Thymosin beta-4	4744.40	4744.41	-2	N-term acetylation	3.1E-68

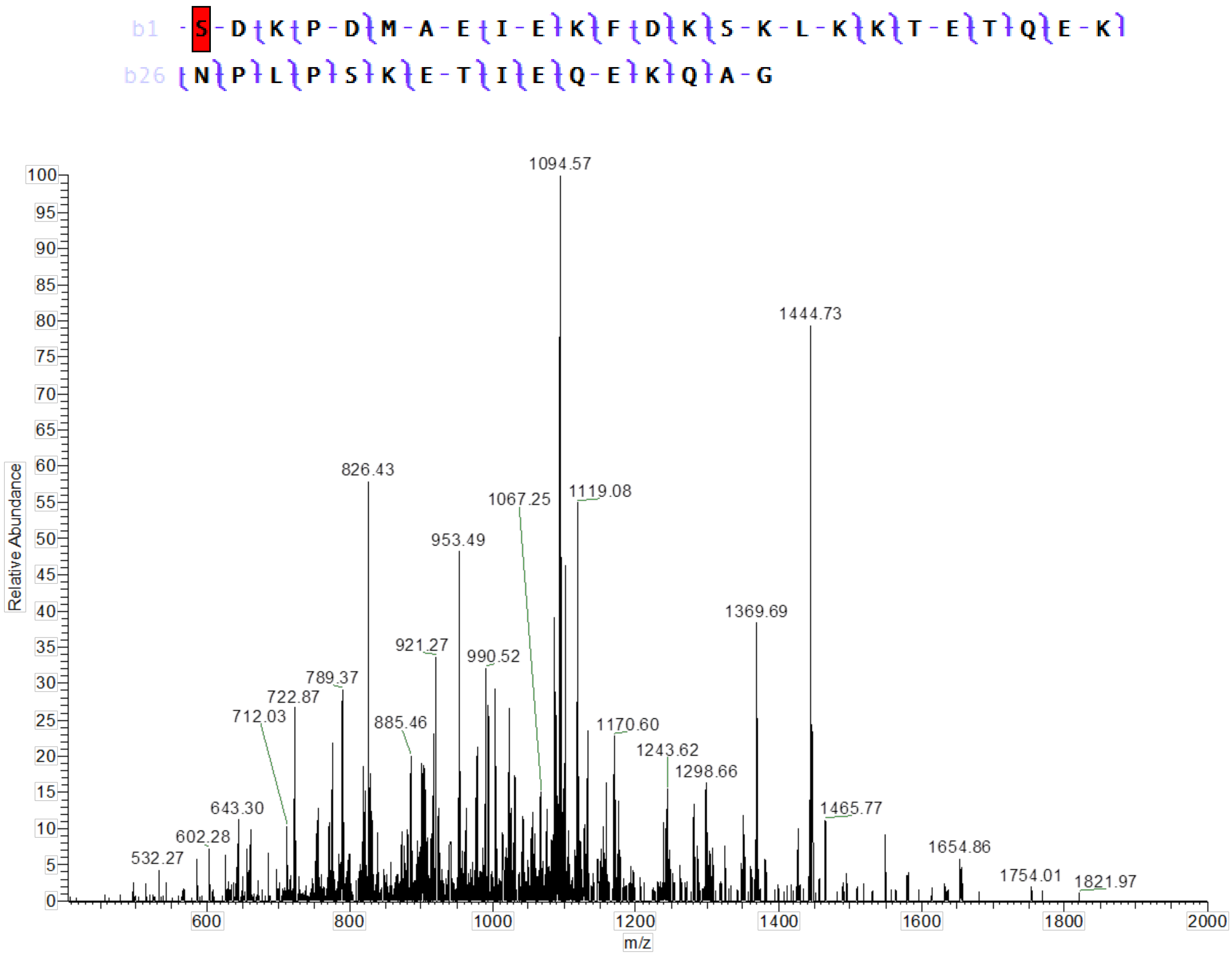

Table S2

TYB10_RAT	P63312	Thymosin beta-10	4747.42	4747.42	0	N-term acetylation, C-term/R methylation	6.9E-72

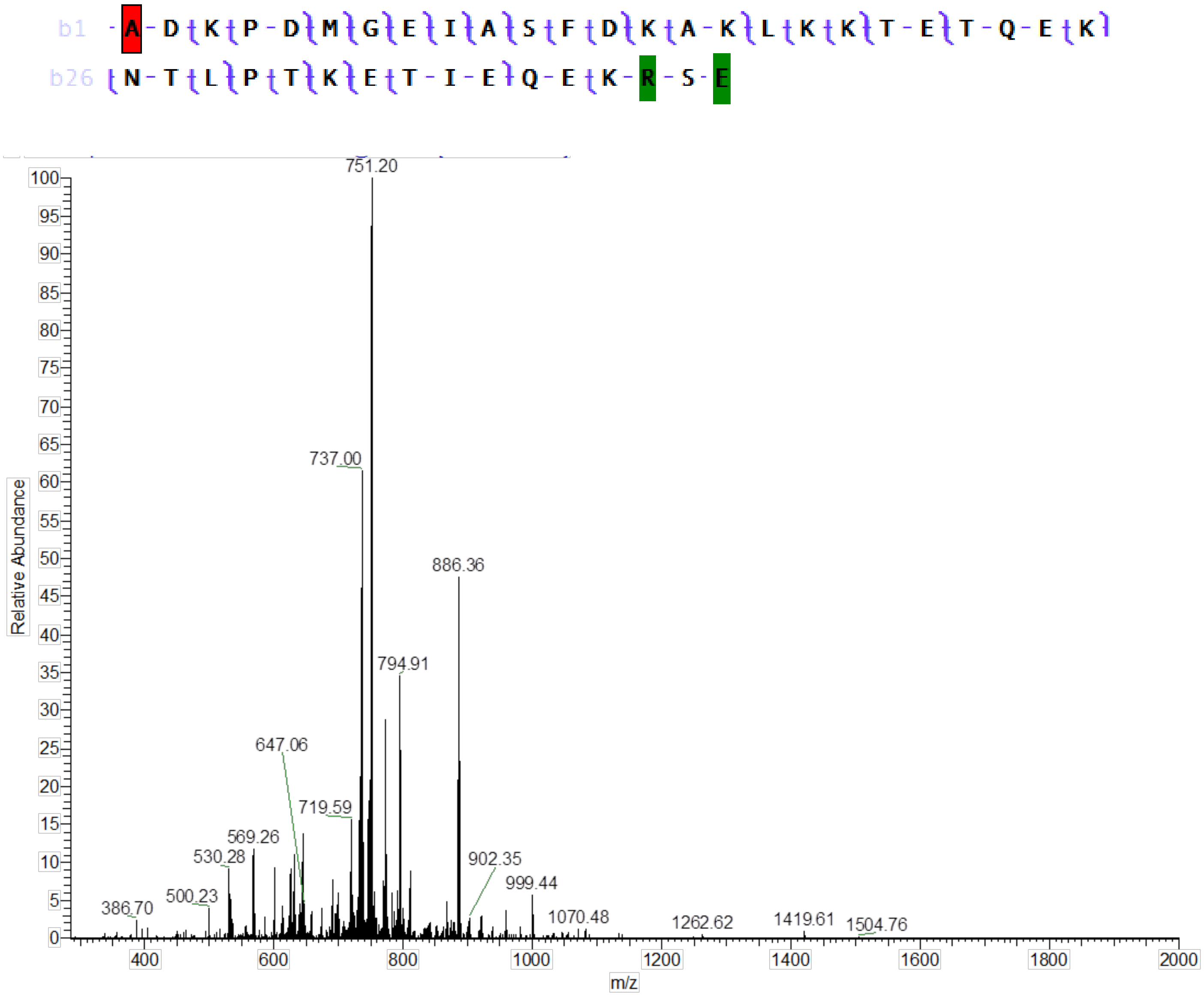

Table S2

TYB10_RAT	P63312	Thymosin beta-4	4758.42	4758.43	-2	N-term acetylation, C-term methylation	1.2E-81

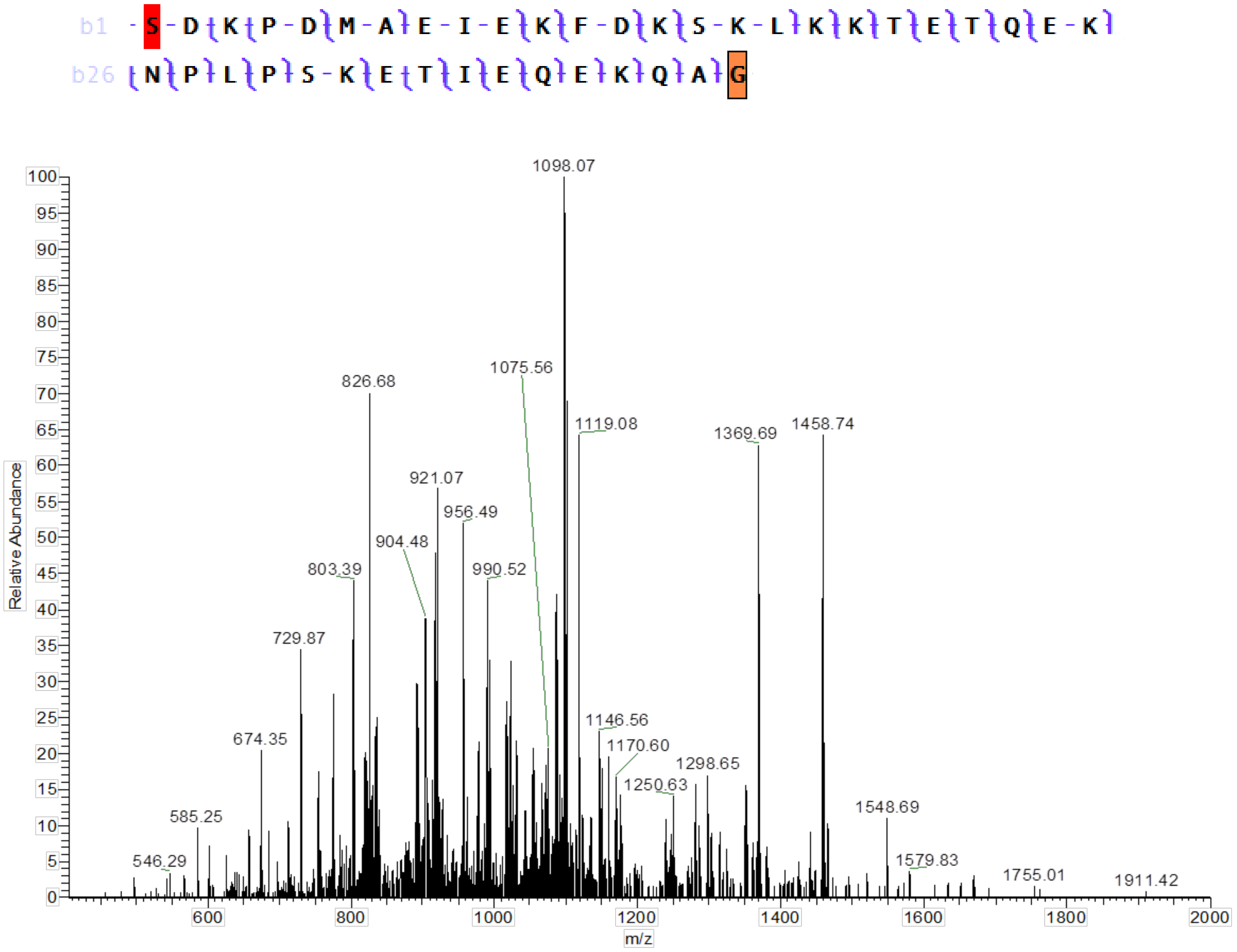

Table S2

D3ZWN0_RAT	D3ZWN0	Protein Plekha6	4774.41	4774.42	-2		1.6E-14

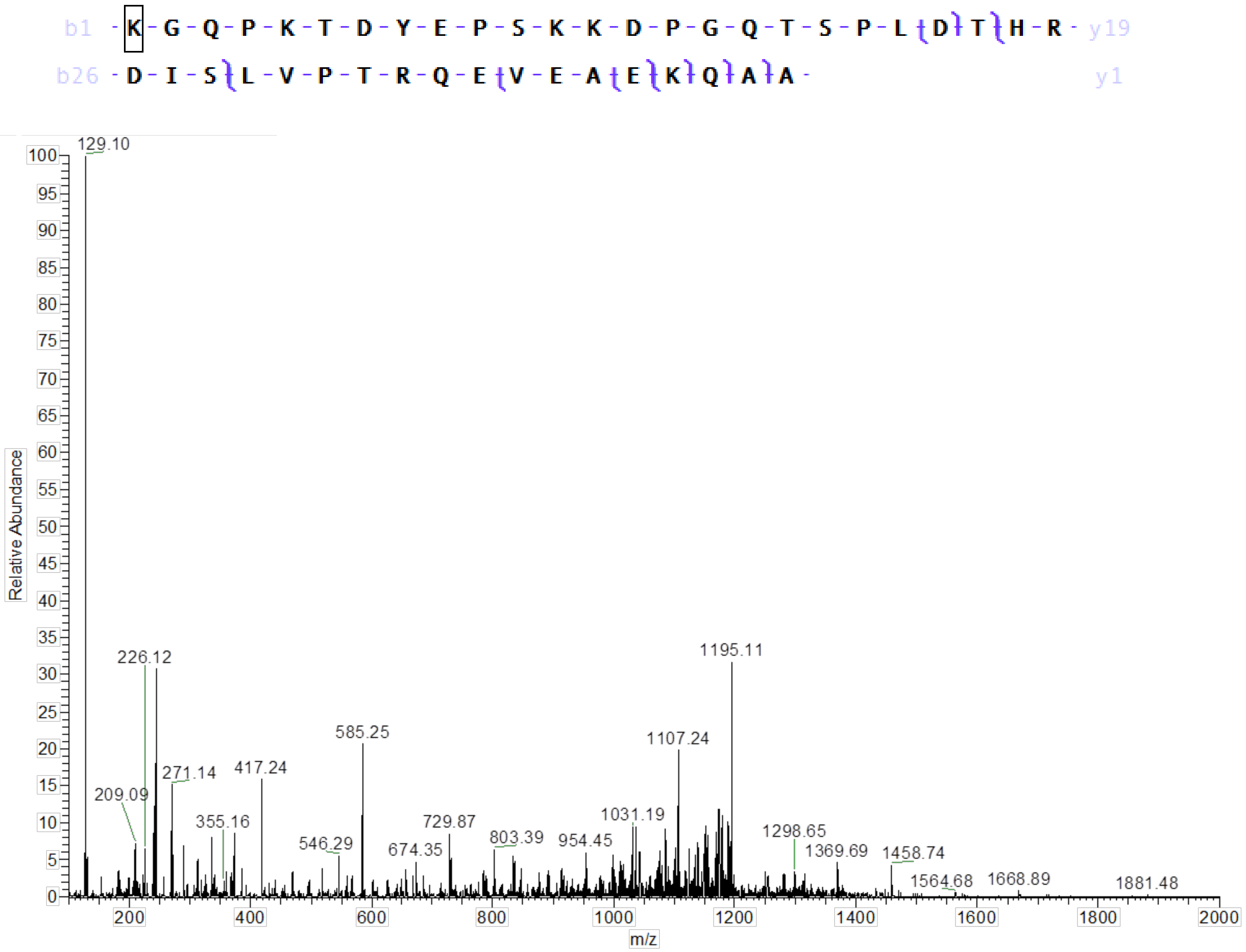

Table S2

TYB10_RAT	P63312	Thymosin beta-4	4976.47	4976.48	-2	N-term acetylation, oxidation	7.7E-86

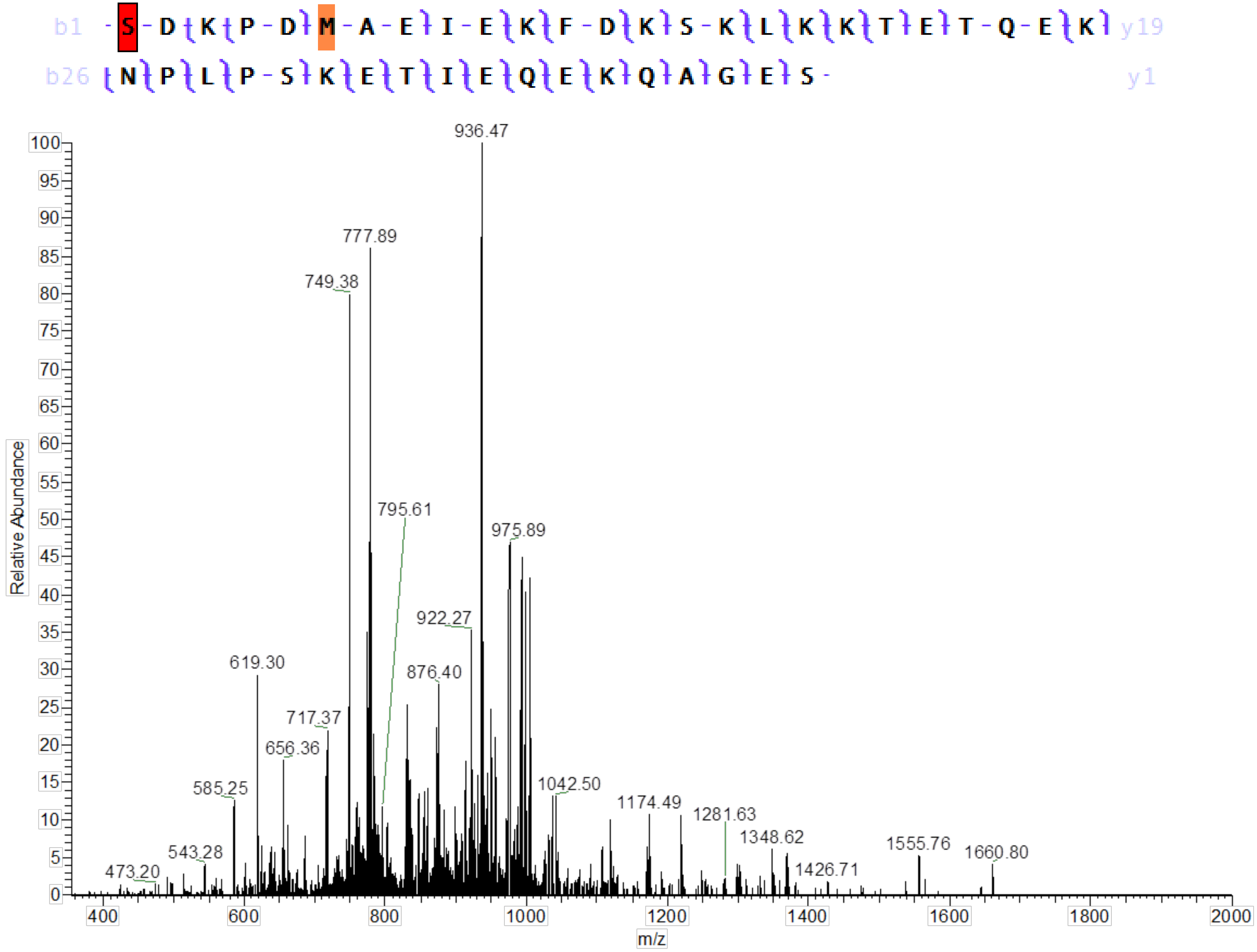

Table S2

F1LUV9_RAT	F1LUV9	Uncharacterized	5241.65	5241.65	0		3.0E-39

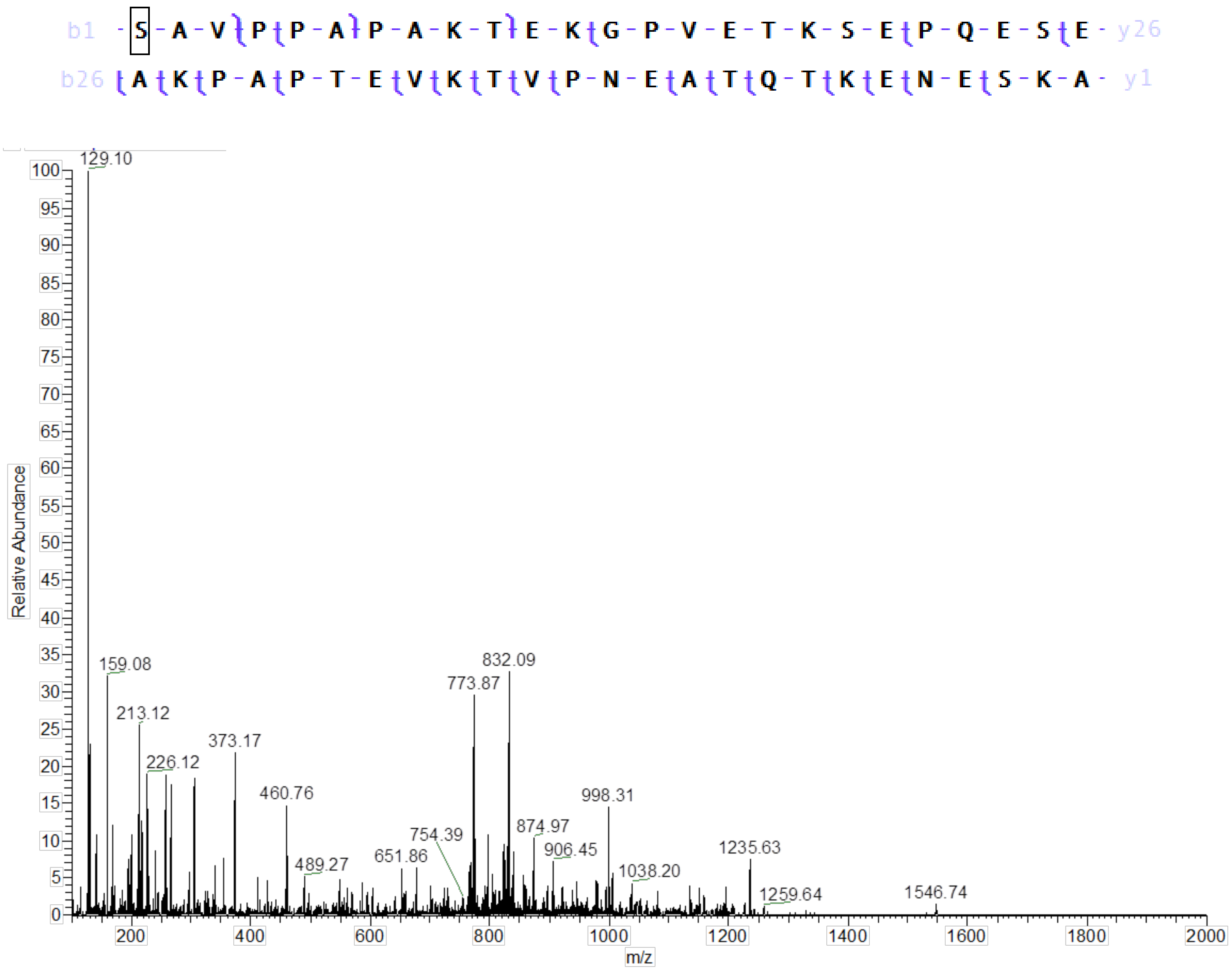

Table S2

NEUM_RAT	P07936	Neuromodulin	5261.39	5261.40	-2		5.7E-86

b1 A-A K A A-Q P P-T-E T-A E S S Q A E E E K E A-V D y25

b26 E A K P-K-E S-A-R-Q-D E G-K E D P-E A-D-Q-E H-A- y1

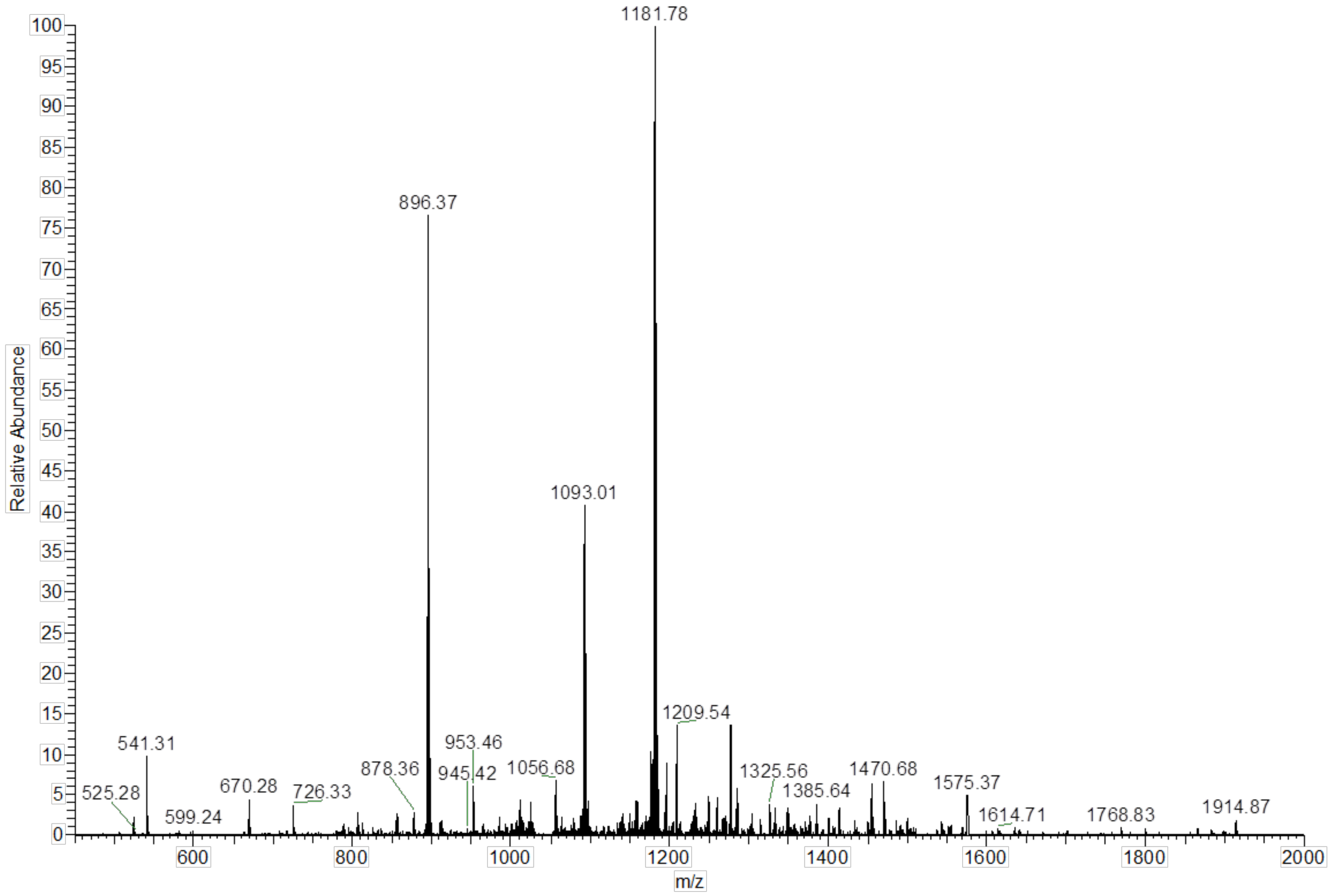

Table S2

CCKN_RAT	P01355	CCK	5434.99	5435.00	-2		7.7E-44

c1 A-V-L-R-P-D-S-E-P-R-A-R-L-G-A-L-L-A-R-Y-I-Q-Q-V-R-

c26 K-A-P-S-G-R-M-S-V-L-K-N-L-Q-G-L-D-P-S-H-R-I-S-D

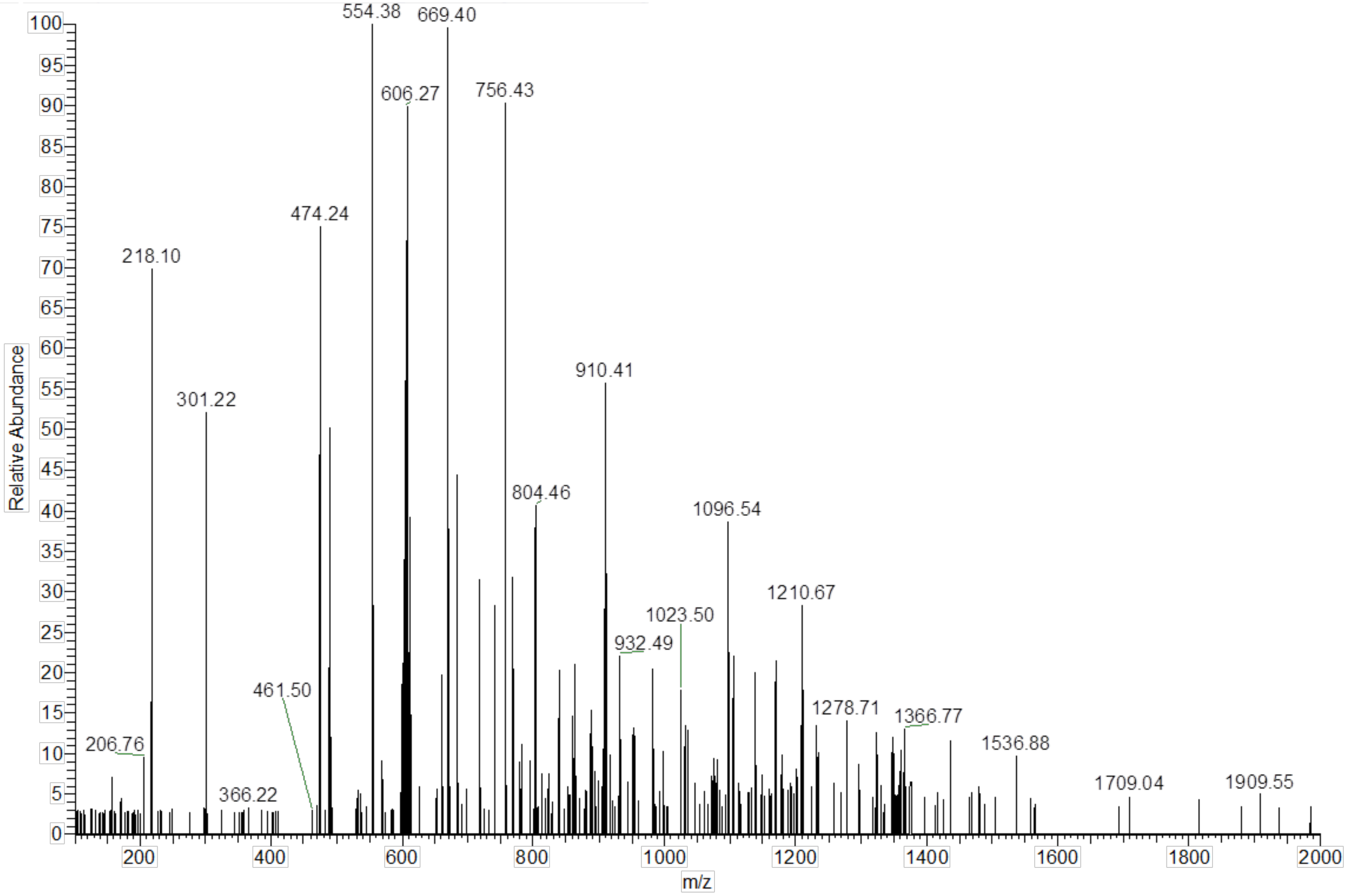

Table S2

D4AA63_RAT	D4AA63	Ubiquitin	5895.01	5895.01	0		1.0E-55

b1 - G - S - P - P - Q - P - P - N - P - E - V - R - F - Q - Q - Q - L - E - Q - L - N - A - M - G - F - y31
b26 - L - N - R - E - A - N - L - Q - A - L - I - A - T - G - G - D - I - N - A - A - I - E - R - L - L - y6
b51 - G - S - Q - P - S - y1

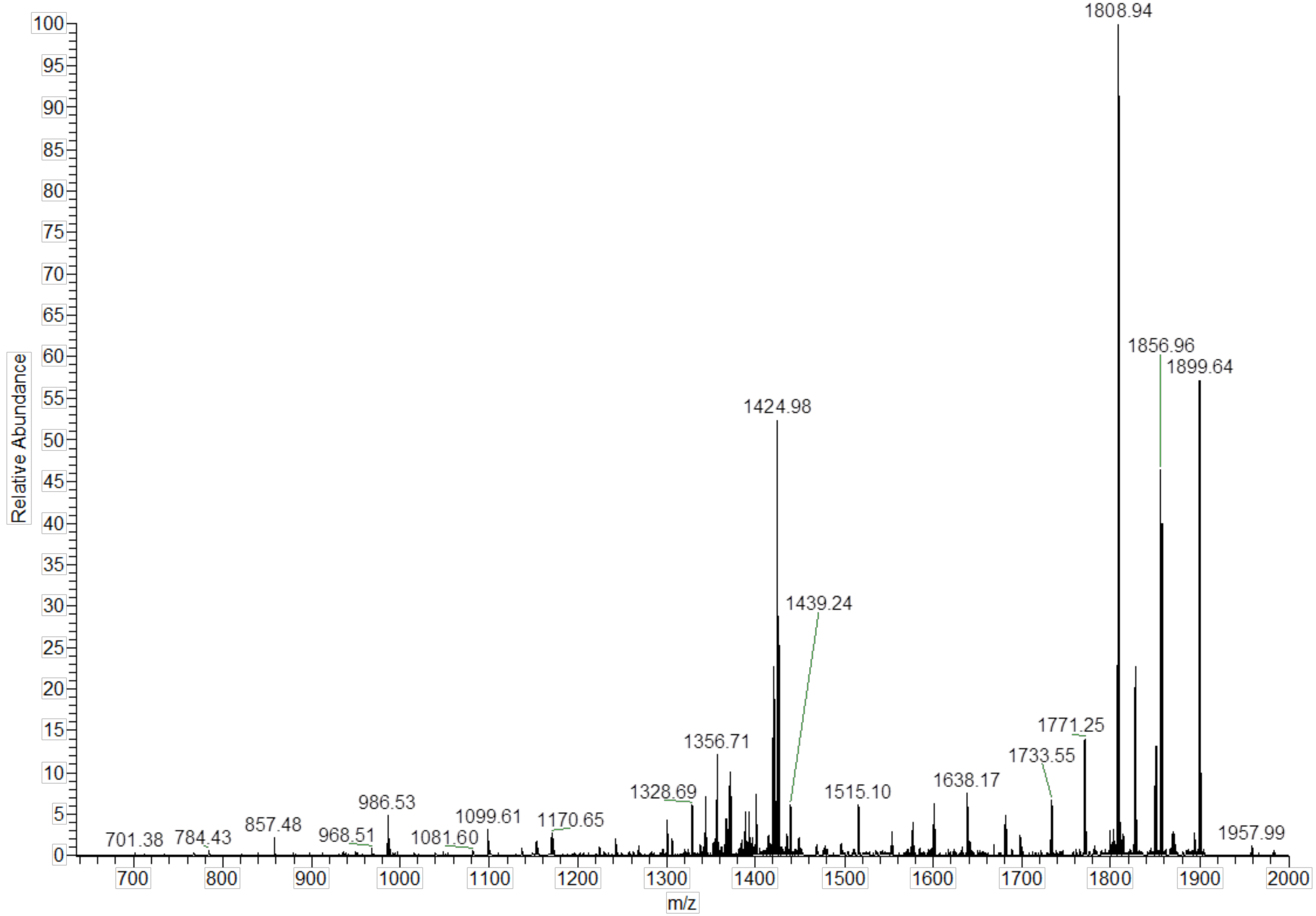

Table S2

ATP5J_RAT	P21571	ATP synthase-coupling factor 6	6024.88	6024.89	-2		4.0E-15

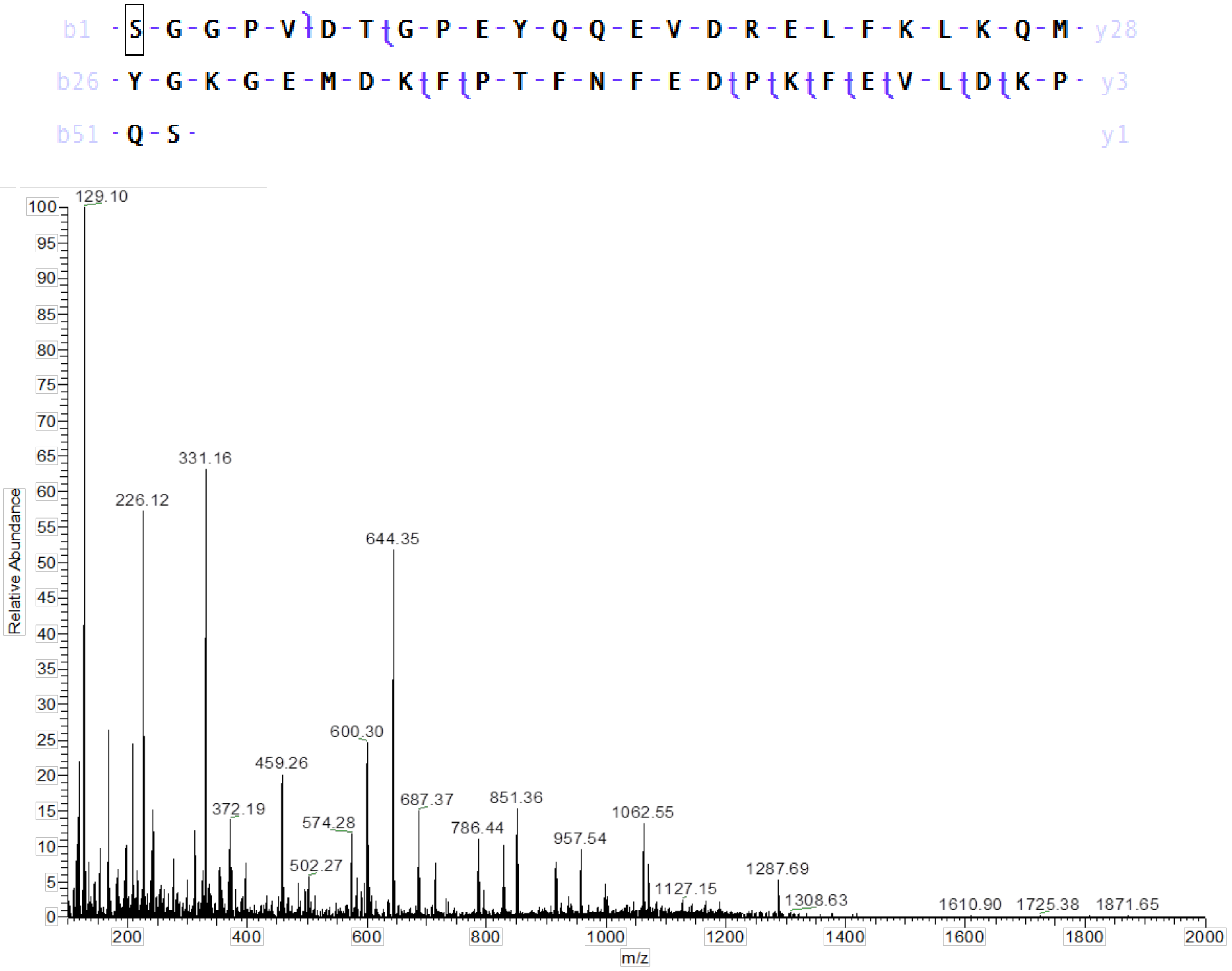

Table S2

PCP4_RAT	P63055	PEP-19	6730.24	6730.25	-1	N-term acetylation, oxidation	4.4E-32

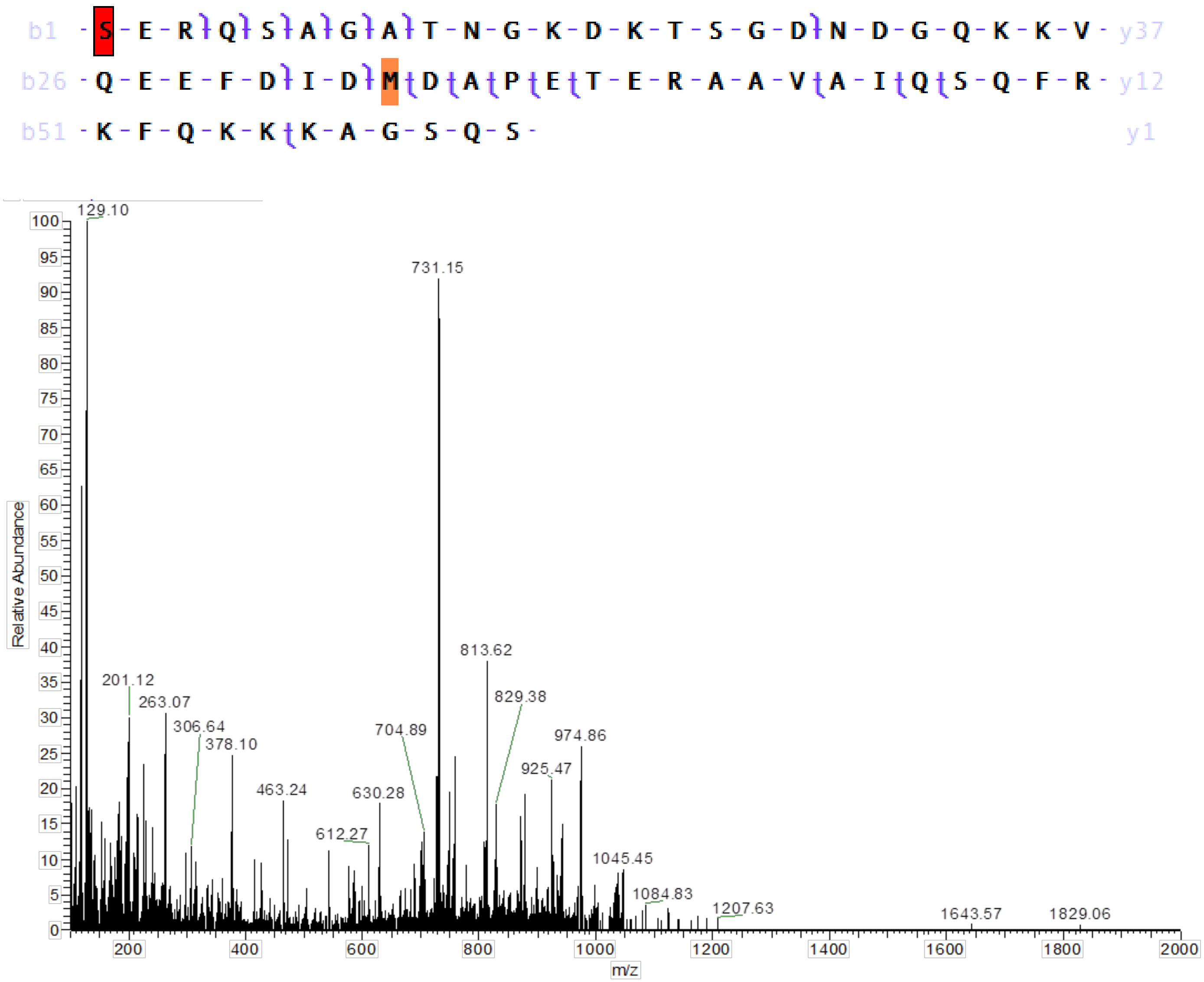

Table S2

NEUM_RAT	P07936	Neuromodulin	7963.66	7963.70	-5		1.7E-25

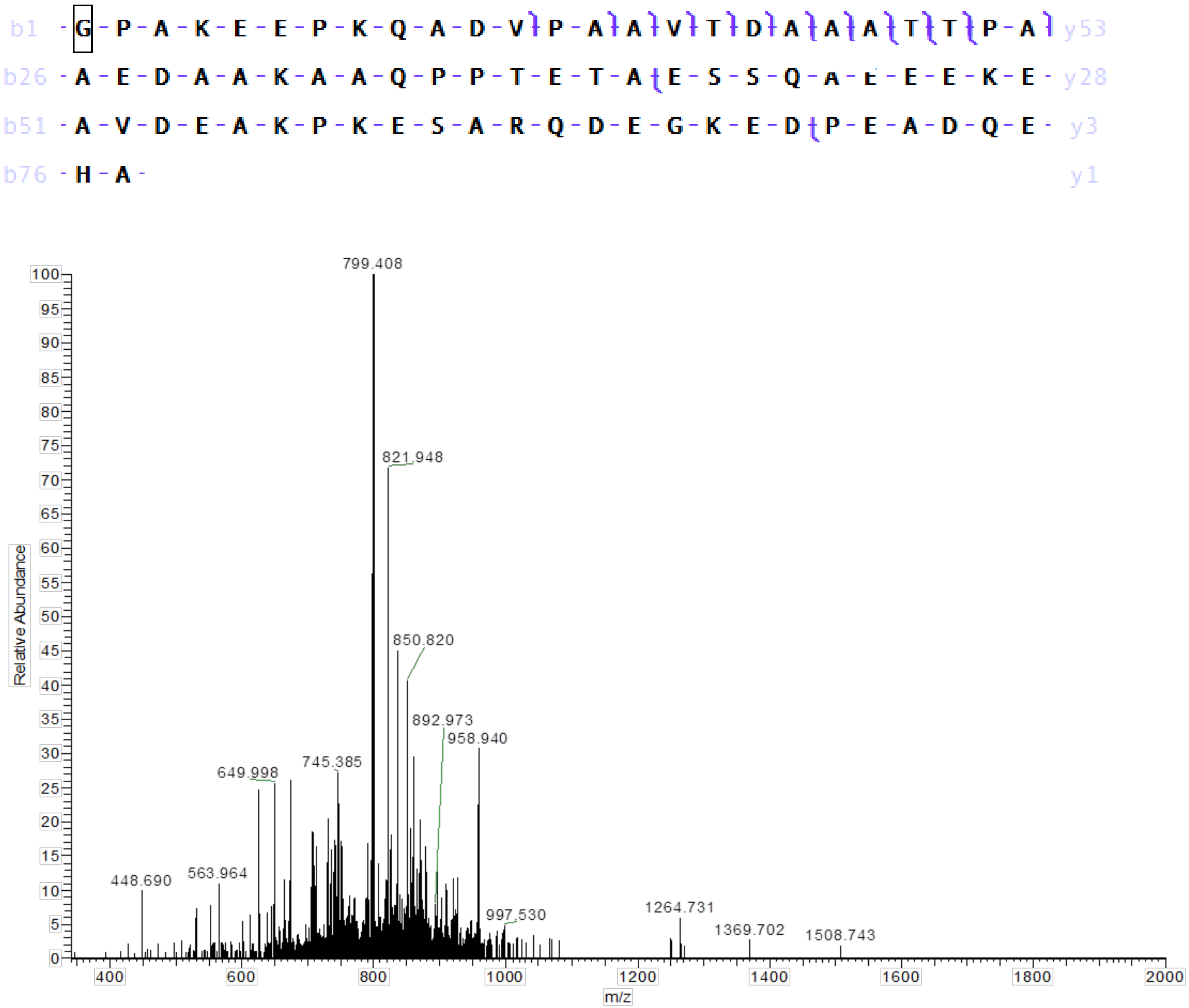

Table S2

F1LNN9_RAT	F1LNN9	Uncharacterized protein	8155.96	8155.94	2		3.9E-28

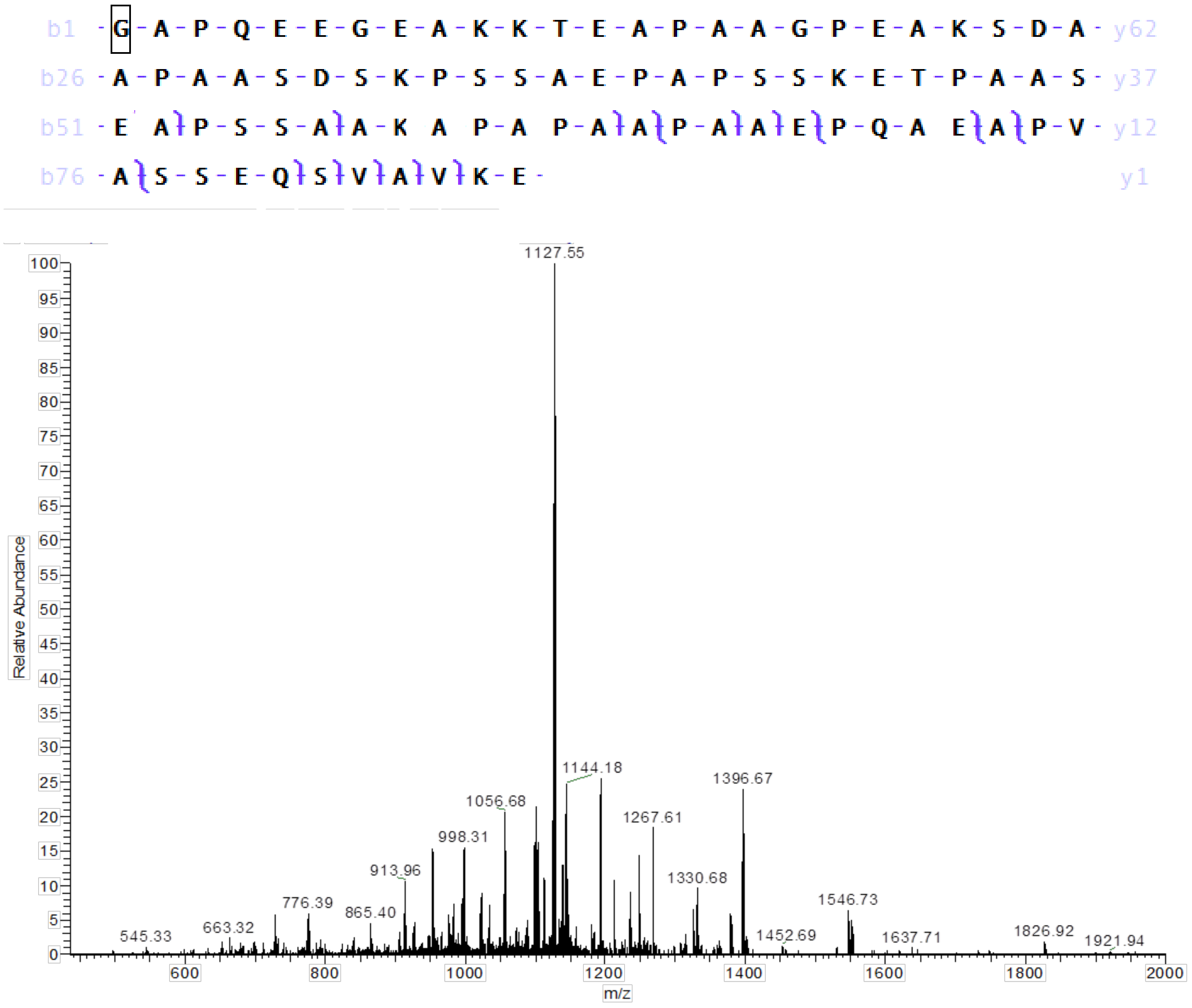

Table S2

RS27A_RAT	P62982	Ubiquintin-40S ribosomal protein S27a	8445.57	8445.57	0		2.1E-43

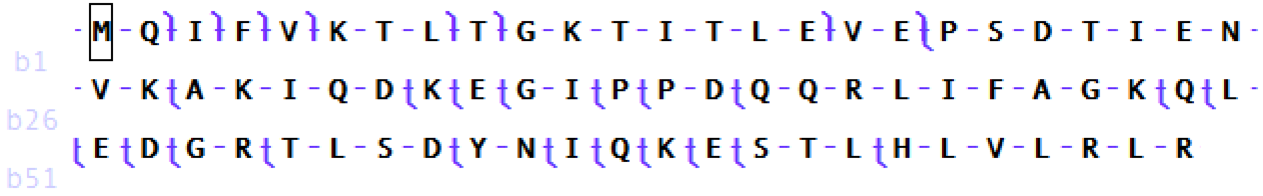

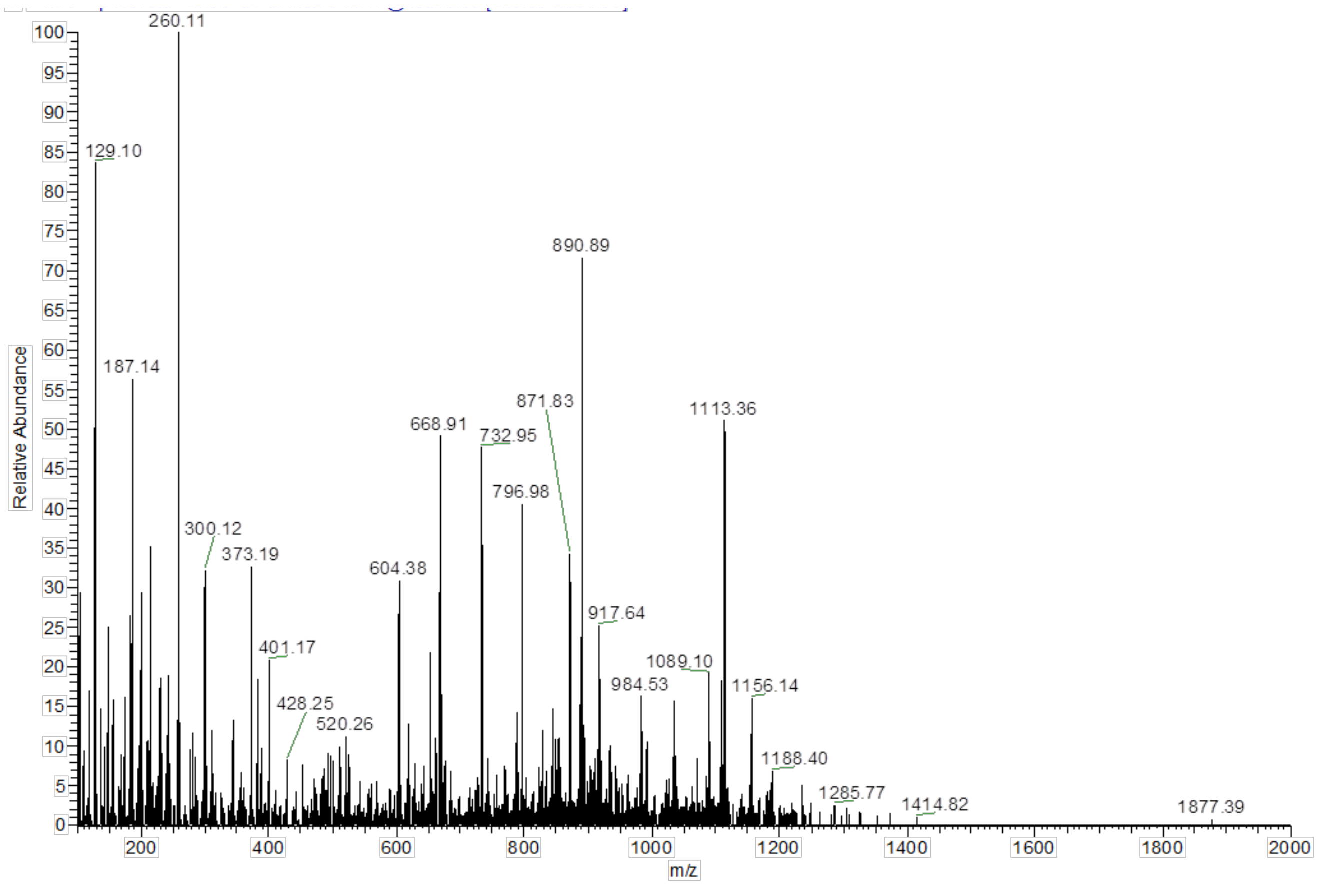

Table S2

RS27A_RAT	P62982	Ubiquintin-40S ribosomal protein S27a	8459.59	8459.59	0	C-terminal/R methylation	2.7E-32

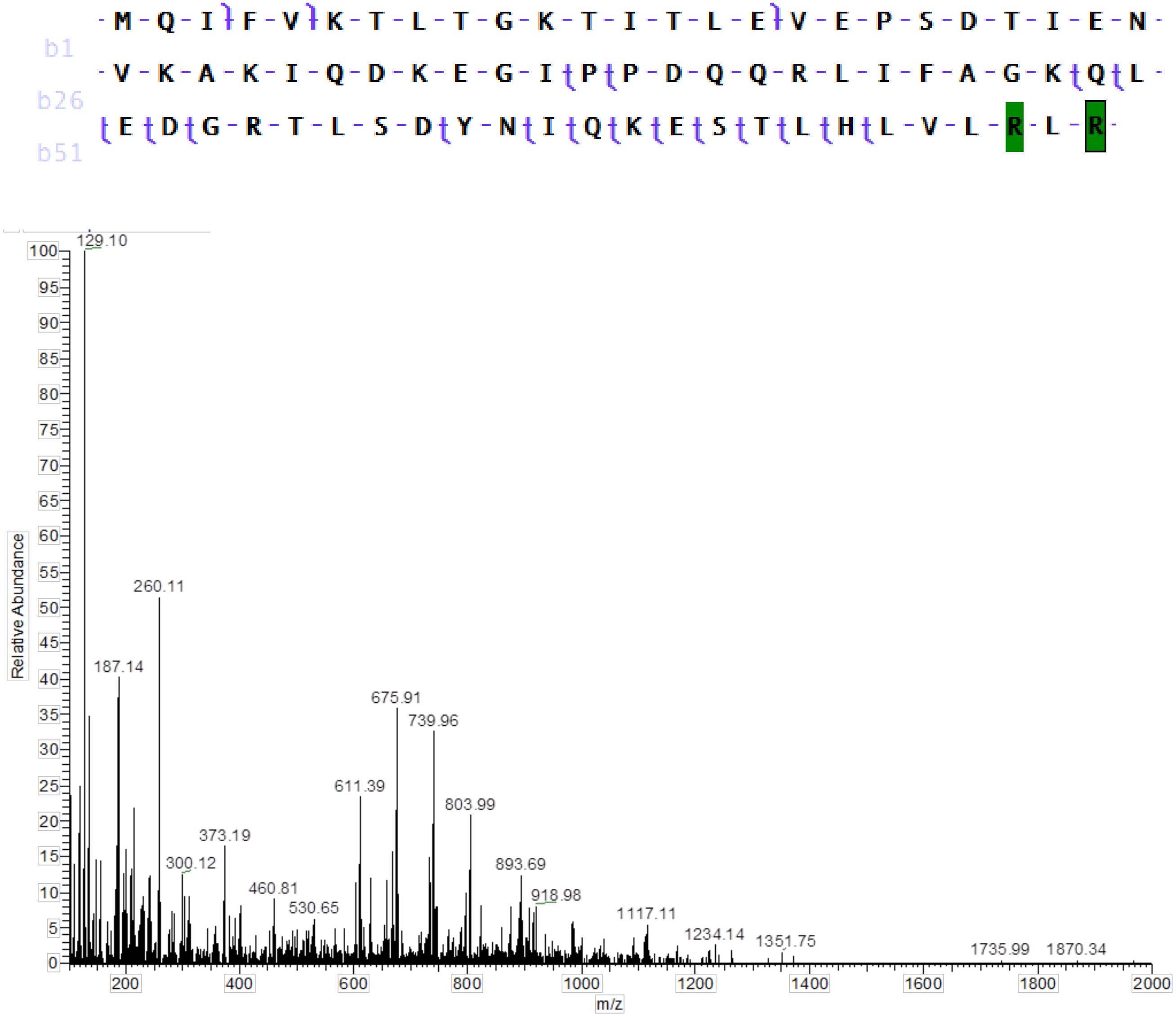

Table S2

NEDD8_RAT	Q71UE8	NEDD8	8554.67	8554.67	0.00		2.1E-34

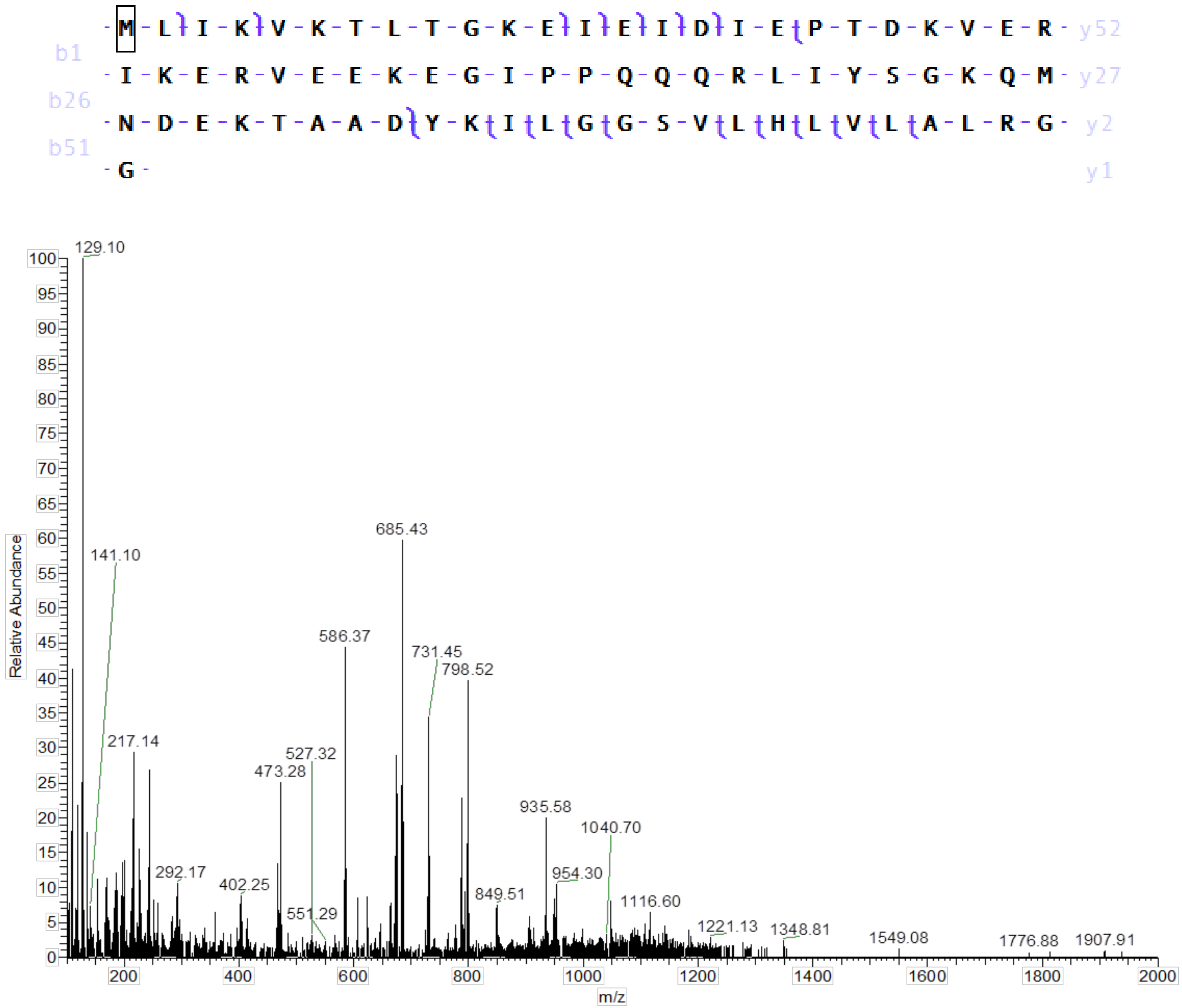

Table S2

RS27A_RAT	P62982	Ubiquintin-40S ribosomal protein S27a	8575.60	8575.61	-1	Oxidation	2.0E-48

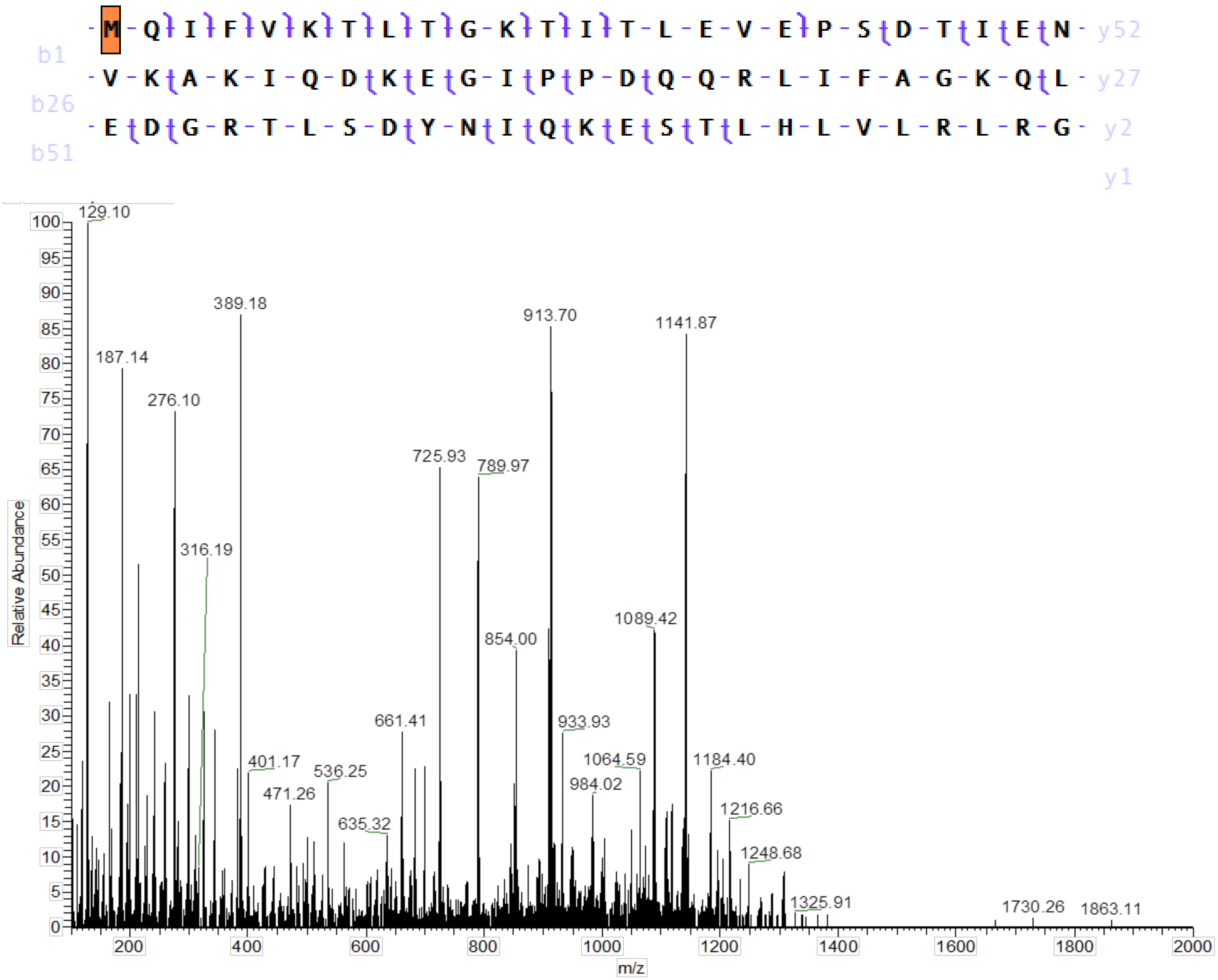

Table S2

UFM1_RAT	Q5BJP3	Ubiquitin-fold modifier 1	8832.80	8832.8	0	N-terminal acetylation	1.1E-41

b1 - S - K - V - S - F - K - I - T - L - T - S - D - P - R - L - P - Y - K - V - L - S - V - P - E - S - y58
b26 - T - P - F - T - A - V - L - K - F - A - A - E - E - F - K - V - P - A - A - T - S - A - I - I - T - y33
b51 - N - D - G - I - G - I - N - P - A - Q - T - A - G - N - V - F - L - K - H - G - S - E - L - R - L - y8
b76 - I - P - R - D - R - V - G - y1

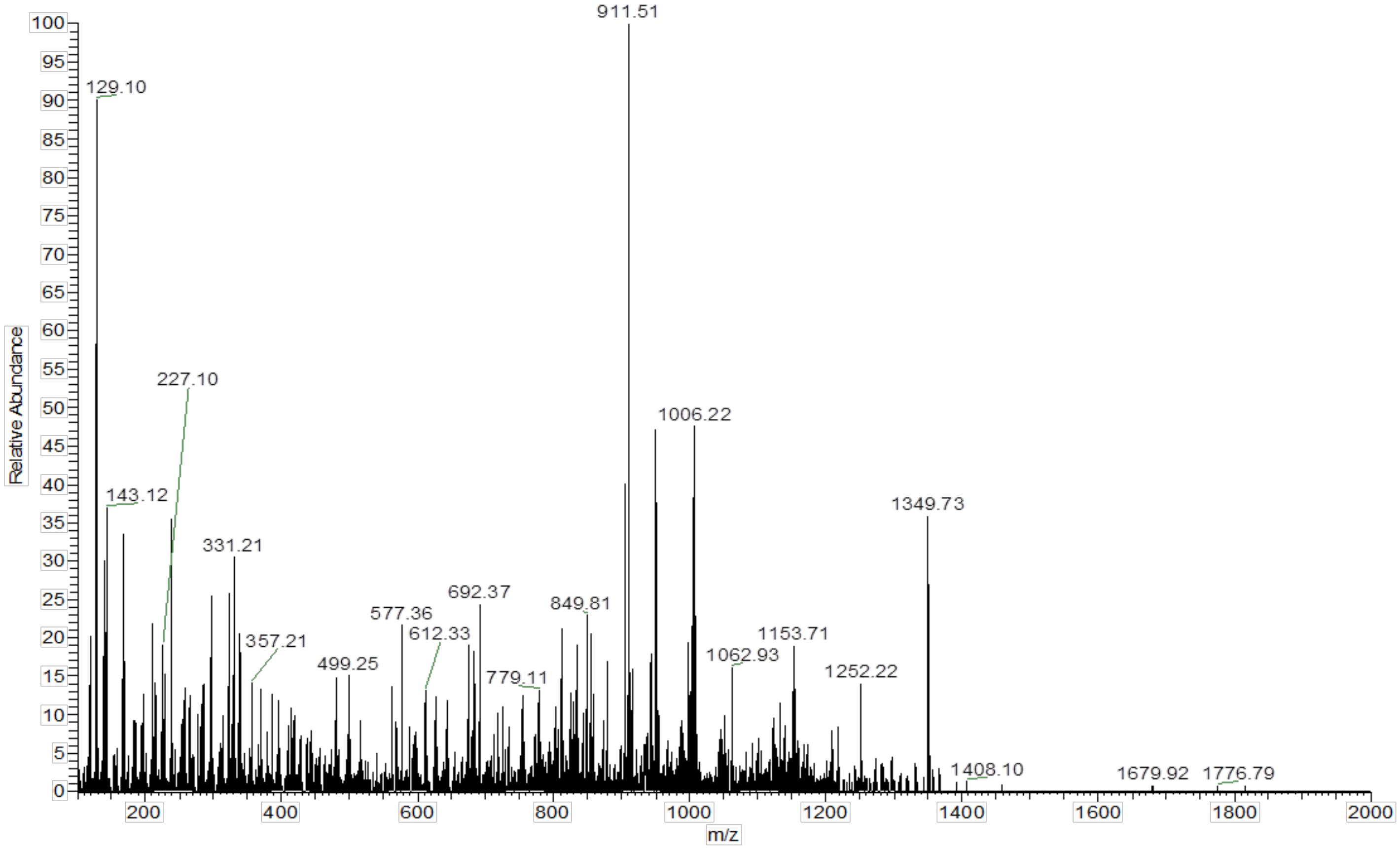

Table S2

ATP5J_RAT	P21571	ATP synthase-coupling factor 6	8937.52	8937.55	-3	Oxidation	1.5E-37

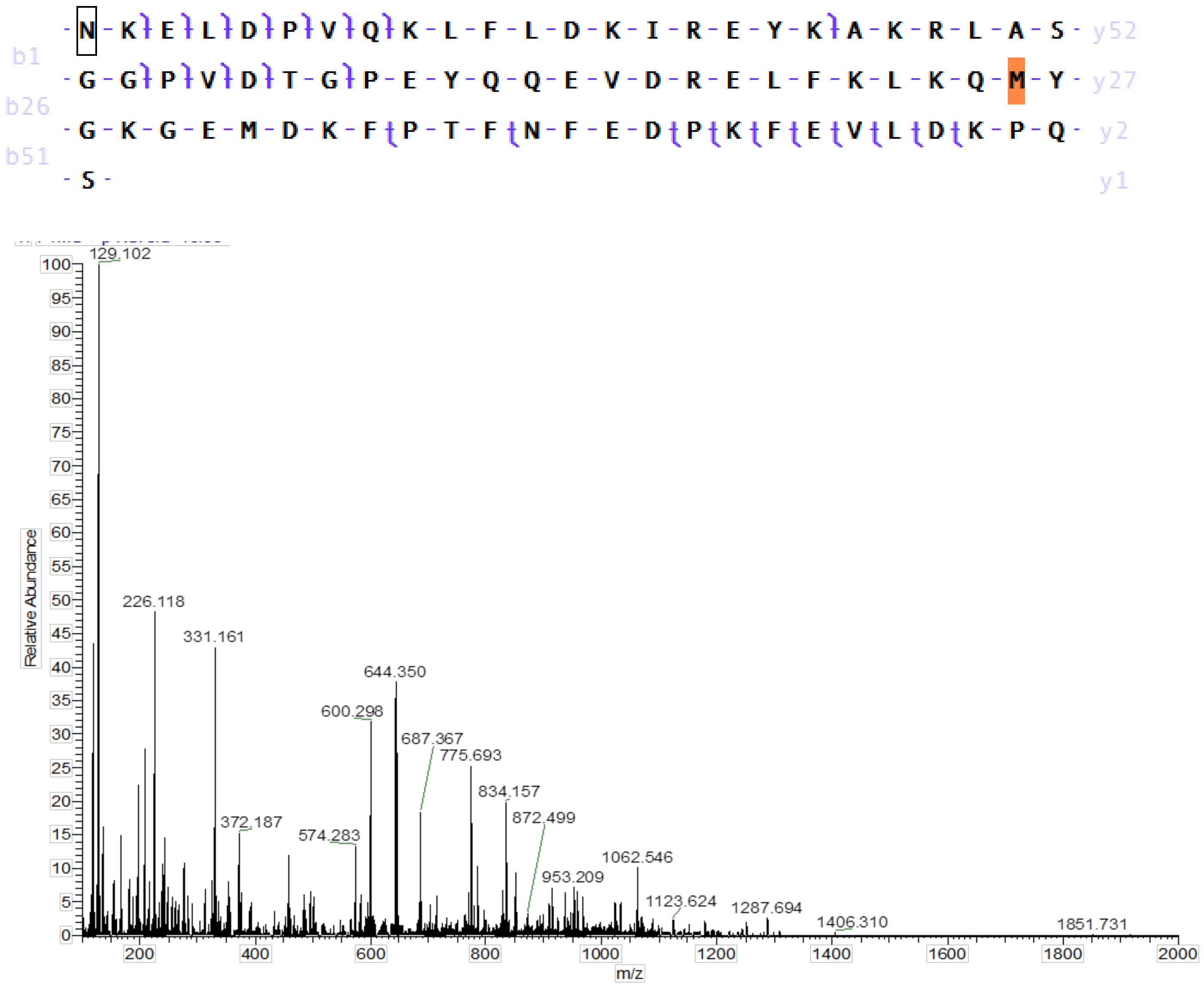

Table S2

ACBP_RAT	P11030	Acyl-CoA-binding protein	9762.01	9762.02	-1	N-term acetylation	9.4E-13

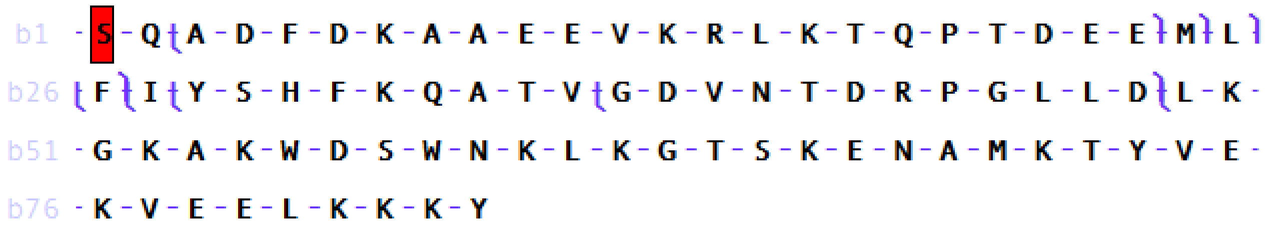

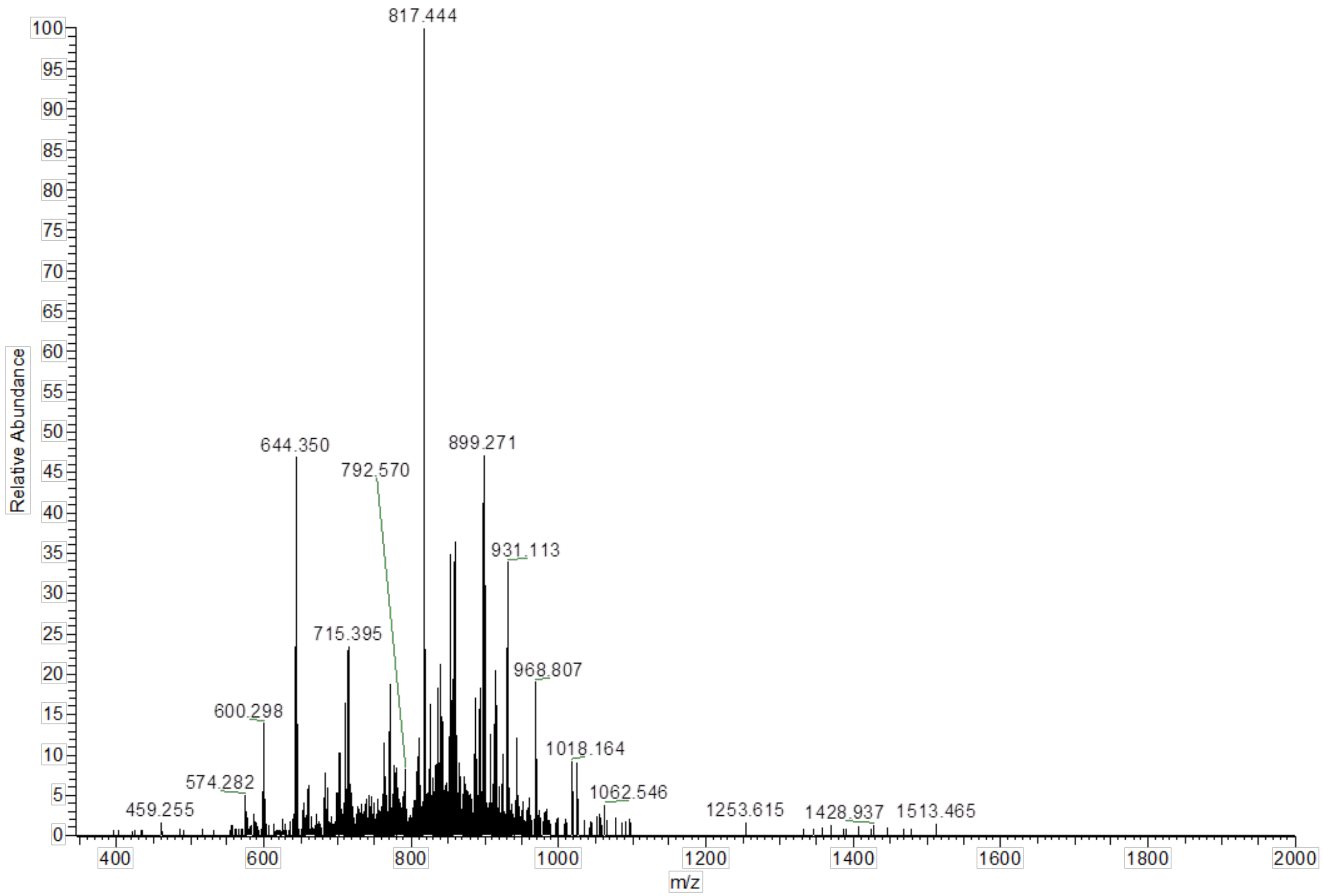

Table S2

DLRB1_RAT	P62628	Dynein light chain roadblock-type 1	10893.73	10893.73	0	N-term acetylation	2.0E-53

b1 - A - E - V - E - E - T L - K - R - L Q S - Q - K G - V Q G I I - V V N T - E y71
b26 - G I P - I - K S - T - M - D N P - T - T T - Q Y - A - N - L - M - H - N - F - I - L - y46
b51 - K - A - R - S - T - V - R - E - I - D P - Q - N - D L - T - F - L - R - I - R - S - K - K - N - y21
b76 - E I - M - V A P - D K - D Y - F - L - I V I Q - N P - T - E - y1

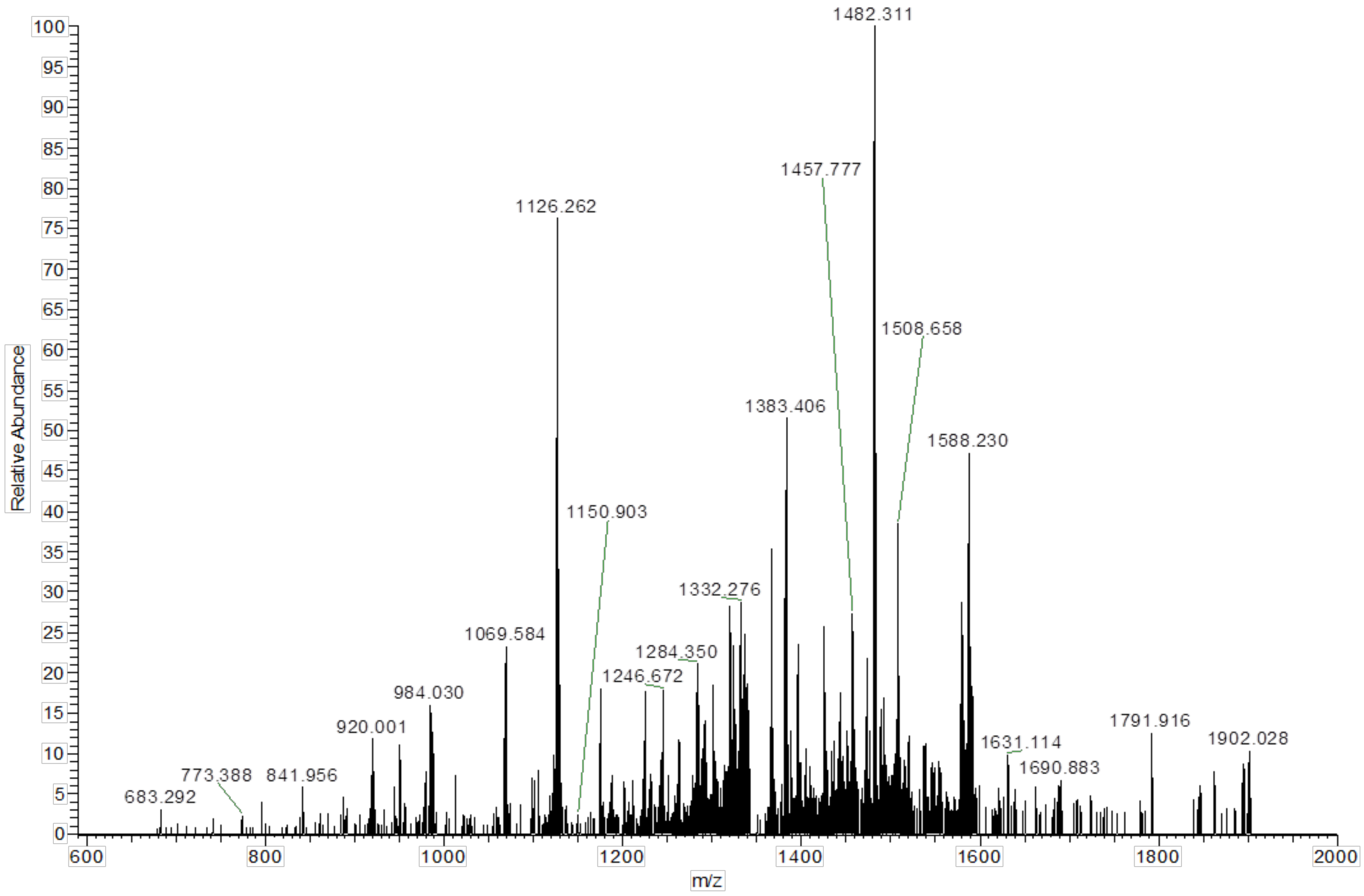

Supplement: File S3 — Top-down MSMS spectra. Raw top-down MS/MS spectra and fragmentation maps of all the proteins identified on nanoLC-ESI-LTQ-Orbitrap Elite listed in the order as they are shown in Table 1 /Table S1, Table 2 and Table S2. (PDF) [file pone.0092831.s003.pdf]
